# Lung‐delivered IL‐10 mitigates Lung inflammation induced by repeated endotoxin exposures in male mice

**DOI:** 10.14814/phy2.70253

**Published:** 2025-02-20

**Authors:** Aaron D. Schwab, Todd A. Wyatt, Oliver W. Schanze, Amy J. Nelson, Angela M. Gleason, Michael J. Duryee, Deanna D. Mosley, Geoffrey M. Thiele, Ted R. Mikuls, Jill A. Poole

**Affiliations:** ^1^ Division of Allergy & Immunology University of Nebraska Medical Center Omaha Nebraska USA; ^2^ Division of Pulmonary, Critical Care & Sleep University of Nebraska Medical Center Omaha Nebraska USA; ^3^ Veterans Affairs Nebraska‐Western Iowa Health Care System Research Service Omaha Nebraska USA; ^4^ Department of Environmental, Agricultural and Occupational Health, College of Public Health University of Nebraska Medical Center Omaha Nebraska USA; ^5^ Division of Rheumatology & Immunology, Department of Internal Medicine, College of Medicine University of Nebraska Medical Center Omaha Nebraska USA

**Keywords:** endotoxin, environmental lung disease, inflammation, macrophages, occupational

## Abstract

Therapies capable of resolving inflammatory lung disease resulting from high‐consequence occupational/environmental hazards are lacking. This study seeks to determine the therapeutic potential of direct lung‐delivered interleukin (IL)‐10 following repeated lipopolysaccharide exposures. C57BL/6 mice were intratracheally instilled with LPS (10 μg) and treated with IL‐10 (1 μg) or vehicle control for 3 days. Lung cell infiltrates were enumerated by flow cytometry. Lung sections were stained for myeloperoxidase (MPO), CCR2, vimentin, and post‐translational protein citrullination (CIT) and malondialdehyde‐acetaldehyde (MAA) modifications. Lung function testing and longitudinal in vivo micro‐CT imaging were performed. Whole lungs were profiled using bulk RNA sequencing. IL‐10 treatment reduced LPS‐induced weight loss, pentraxin‐2, and IL‐6 serum levels. LPS‐induced lung proinflammatory and wound repair mediators (i.e., TNF‐α, IL‐6, CXCL1, CCL2, MMP‐8, MMP‐9, TIMP‐1, fibronectin) were decreased with IL‐10. IL‐10 reduced LPS‐induced influx of lung neutrophils, CD8^+^ T cells, NK cells, recruited monocyte‐macrophages, monocytes, and tissue expression of CCR2^+^ monocytes‐macrophages, MPO^+^ neutrophils, vimentin, CIT, and MAA. IL‐10 reduced LPS‐induced airway hyperresponsiveness and improved lung compliance. Micro‐CT imaging confirmed the reduction in LPS‐induced lung density by IL‐10. Lung‐delivered IL‐10 therapy administered after daily repeated endotoxin exposures strikingly reduces lung inflammatory and wound repair processes to decrease lung pathologic changes and mitigate airway dysfunction.

## Graphical abstract

A graphical summary of the experimental murine experimental design, magnitude of transcriptomic and micro‐CT imaging changes associated with IL‐10 therapy following LPS exposures, with an overview of the associated lung cellular and mediator events demonstrated in this study.

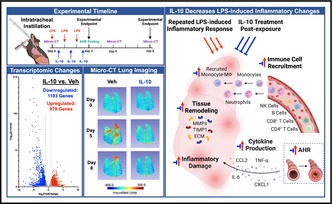

## 1 INTRODUCTION

Occupational exposure‐induced lung diseases are the primary cause of occupation‐associated illness in the United States, with 15% of all chronic obstructive lung disease in Western societies (~35 million persons) primarily attributed to occupational exposures in the mining, textile, and farming sectors, and 15%–20% of the overall adult asthma burden attributed to occupational exposures (De Matteis et al., 2017; Tarlo & Lemiere, 2014). Agricultural and textile work, as well as emerging sectors such as waste treatment, recycling, biotech food production, and processing industries, are characterized by high endotoxin burden environments (De Matteis et al., 2017). Whereas both acute and repeated endotoxin exposure(s) can increase exposed workers' risk of developing lung diseases such as chronic bronchitis, asthma, and pulmonary fibrosis, risk mitigation strategies are currently the only available means to decrease the burden posed by chronic lung disease developing in the wake of environmental exposures (Schwab & Poole, 2023). Moreover, other high‐consequence occupational and environmental exposures, such as those resulting from natural disasters, catastrophic incidents, or other occupational accidents that are highly concentrated over a definitive time period (Krefft et al., 2015; Morimoto et al., 2021; Reid & Maestas, 2019; Robinson et al., 2011), can also set the stage for progressive lung disease. Given the unpredictable nature of these events, risk mitigation strategies are insufficient to address the human health risks that can result from such high‐consequence exposures. Symptomatic management by means of oxygen, bronchodilators, and sometimes corticosteroids can improve patient quality of life; however, there are no interventions capable of hastening lung pathologic recovery and progression to chronic lung disease (Goldman & Schafer, 2012).

Interleukin (IL)‐10 represents a potentially efficacious post‐exposure intervention due to its ability to mitigate tissue damage, reduce inflammation, affect wound repair and fibrosis processes, and promote recovery (Steen et al., 2020). Following tissue injuries that lead to acute lung inflammation, circulating inflammatory CCR2^+^ monocytes are recruited to the lung, differentiate, and persist as monocyte‐derived macrophages (Misharin et al., 2017; Poole, Gaurav, et al., 2022). These recruited, transitioning monocyte/macrophages are critical drivers of lung disease pathology, capable of dysregulating wound repair processes leading to fibrosis (Long et al., 2023; Perrot et al., 2023). Notably, these cells also exhibit high surface expression of the IL‐10 receptor, emphasizing the potential to modulate monocyte/macrophage immune responses with IL‐10 treatment (Iyer & Cheng, 2012). Additionally, infiltrating neutrophils induced by inflammatory exposures can cause lung damage through the release of proteolytic enzymes, by perpetuating oxidative stress, and through the formation of neutrophil extracellular traps (Yang et al., 2021). Elevated levels of pro‐inflammatory cytokines (i.e., TNF‐α, IL‐6), chemoattractants (i.e., CXCL1, CCL2, CCL7), and wound repair mediators (i.e., matrix metalloproteinases and extracellular matrix proteins) characterize the resultant lung microenvironment, and subsequent lung functional deficits can be observed (Gerber et al., 2020; Schwab, Wyatt, Moravec, et al., 2024; Sundblad et al., 2009). Resultant reactive oxygen species‐induced lipid peroxidation and proinflammatory processes induce post‐translational protein modifications with malondialdehyde‐acetaldehyde (MAA) and citrulline (CIT), respectively, which contribute to chronic disease development (Duryee et al., 2021; England et al., 2019; Samara et al., 2017).

Although no IL‐10 therapies have been clinically approved to date, systemic administration of IL‐10 has been explored for the treatment of inflammatory diseases, such as inflammatory bowel disease, psoriasis, rheumatoid arthritis, and pancreatitis, and although generally well tolerated, clinical outcome goals were not met (Carlini et al., 2023; Saraiva et al., 2020). Systemic and long‐term delivery of IL‐10 has been associated with flu‐like symptoms, anemia, thrombocytopenia, elevated liver transaminase levels, and injection site reactions (Patilas et al., 2024), emphasizing the need for novel and directed IL‐10 administration strategies. Our prior studies demonstrated that short‐term, lung‐delivered IL‐10 reduced several inflammatory consequences, including a reduction of TNF‐⍺ and IL‐6, following a one‐time LPS and a swine confinement organic dust extract exposure (Poole, Gaurav, et al., 2022; Schwab, Wyatt, Nelson, et al., 2024). However, it is not known whether a more intense airborne hazard exposure over several days, characteristic of a high‐consequence event, might also benefit from lung‐delivered IL‐10 therapy. Therefore, the objective of this study was to investigate the application of short‐term, lung‐targeted IL‐10 as a therapeutic strategy using an animal model representative of repeated, inhaled LPS exposures coupled with comprehensive evaluations of lung inflammation and function.

## 2 MATERIALS AND METHODS

### 2.1 Mice

C57BL/6 mice (age 6–8 weeks) were purchased from The Jackson Laboratory (Bar Harbor, ME). Male mice were utilized for all studies because we and others have previously demonstrated that female mice were less susceptible to endotoxin‐induced airway and systemic inflammatory effects (Card et al., 2006; Nelson et al., 2018; Poole et al., 2024). The study was conducted and reported in accordance with ARRIVE guidelines (https://arriveguidelines.org). All animal procedures were approved by the University of Nebraska Medical Center (Omaha, Nebraska, USA) Institutional Animal Care and Use Committee and were in accordance with NIH guidelines for the use of rodents.

### 2.2 Airway inflammation model

Lipopolysaccharide (LPS) from gram‐negative *Escherichia coli* (O55:B5; Sigma (L2880), St. Louis, MO) dosed at high concentration served as the exaggerated inflammatory inhalant exposure. Under light sedation with isoflurane, mice received 10 μg of LPS in 50 μL of sterile saline or saline alone via intratracheal (i.t.) instillation daily for 3 days. Each of the 3 daily LPS/saline exposures was followed by treatment with 1 μg of recombinant IL‐10 (Sino Biological (50245‐MNAE), Wayne, PA) or saline (vehicle) (50 μL volume) 5 h post‐exposure. The 5‐h time point was chosen based upon previous time course studies demonstrating that recruitment of lung monocytes/macrophages was not detected at 5 h but was detected at 24 h and peaked at 48 h following LPS exposure (Poole, Gaurav, et al., 2022). Animals were euthanized 24 h or 5 days after the final LPS exposure.

### 2.3 Pulmonary function

Baseline airway resistance, compliance, and hyperresponsiveness (AHR) were assessed 24 h after the final LPS or saline exposure using a computerized small‐animal ventilator (FinePointe, Buxco Electronics, Wilmington, NC) as previously described (Poole et al., 2009). Dose responsiveness to aerosolized methacholine (0–48 mg/mL) (1,296,364, USP, Rockville, MD) was obtained and reported as total lung resistance (R_L_) and dynamic compliance (C_dyn_).

### 2.4 Blood and serum studies

Peripheral blood differential counts were performed on Diff‐Quick (Siemens, Newark, DE) stained blood smears. Serum was processed as previously described and analyzed via ELISA (R&D, Minneapolis, MN) (Poole et al., 2019). Specifically, Serum pentraxin‐2 (MPTX20), IL‐6 (M600B‐1), and CXCL1 (MKC00B‐1) levels were assessed using a Quantikine ELISA kit (R&D, Minneapolis, MN), according to manufacturer instructions (minimal detection difference [MDD] of 0.159 ng/mL, 1.60 pg/mL, and 2.0 pg/mL, respectively). Serum samples were also analyzed via the VetScan VS2 Chemistry Analyzer using the VetScan Critical Care Plus reagent rotors (500–0042, Zoetis, Parsippany, NJ).

### 2.5 Lavage fluid cells and lung homogenate analyses

Bronchoalveolar lavage fluid (BALF) was collected using 3 × 1 mL of phosphate buffered saline (PBS). Total BALF cell counts from pooled lavages were enumerated using a BioRad TC 20 cell counter. Differential cell counts were determined from cytospin‐prepared slides (Cytopro Cytocentrifuge, ELITech Group, Logan, UT) with Diff‐Quick (Siemens). Cell‐free BALF from the first lavage fraction was evaluated for cytokines and chemokines. After BALF isolation and removal of blood from the pulmonary vasculature, lung tissue homogenates were prepared (Poole et al., 2009). Levels of tumor necrosis factor (TNF)‐α (MTA00B‐1), transforming growth factor (TGF)‐β (DY1679), IL‐6 (M600B‐1), IL‐10 (M1000B‐1), the murine neutrophil chemoattractant CXCL1(MKC00B‐1), the murine leukocyte chemoattractants CCL2 (MJE00B) and CCL7 (ab205571), and the complement component C5a (DY2150) were quantitated by ELISA (R&D Systems) following manufacturer instructions. The kits had MDDs of 1.88, 31.3, 1.6, 31.3, 2.0, 0.3, 1.5, and 15.6 pg/mL, respectively. Lung tissue homogenates were assessed for matrix metalloproteinase (MMP)‐3 (DY548), MMP‐9 (DY6718) and tissue inhibitor of metalloproteinase (TIMP)‐1 (DY980) (ELISA; R&D Systems; MDD of 0.125, 0.078, and 0.031 ng/mL, respectively) and MMP‐8 (ab206982) and fibronectin (ab108849) (ELISA; Abcam, Boston, MA; MDD of 0.053 and 3.125 ng/mL, respectively).

### 2.6 Flow cytometry

Lung cell infiltrates were quantified following lung cell dissociation of the remaining 1/2 of each right lung lobe as previously described (Poole et al., 2019). For immune cell analysis, single‐cell suspension was stained with LIVE/DEAD Fixable Dead Cell stain (BD (L23105)) followed by antibodies against mouse cell surface markers as detailed in Table S1. Post‐acquisition, all flow cytometry data were exported and stored using the flow cytometry standard (FCS) 3.1 format and subsequently analyzed using FlowJo software version 10.10.0 (FlowJo, Ashland, OR). The gating strategies for lung Ly6G^+^ neutrophils, CD11c^+^CD11b^lo^ Alv Mɸ, CD11c^+^CD11b^+^ Act Mɸ, CD11c^int^CD11b^+^ Mono‐Mɸ, and CD11c^−^CD11b^+^ Mono, CD3^+^CD4^+^ T cells, CD3^+^CD8^+^ T cells, CD19^+^ B cells, CD19^+^CD11b^+^ B cells, and NK cells were performed as previously reported (Mikuls et al., 2021; Nelson et al., 2018; Poole et al., 2012, 2017; Poole, Gaurav, et al., 2022; Robbe et al., 2015) with details in Figure S1. Lung cell population numbers were determined by multiplying percent gated by total lung cell numbers.

### 2.7 Histopathology

Left lungs were excised and inflated to 15 cm H_2_O pressure with 10% formalin (Fisher Scientific, Fair Lawn, NJ) for 24 h to preserve pulmonary architecture as previously described (Poole et al., 2019). The fixed, paraffin‐embedded sections were cut (4–5 μm) at midpoint sections to include regions of large and small airways and blood vessels and stained with hematoxylin and eosin (H&E). Tissue sections were semi‐quantitatively assessed for the degree and distribution of lung inflammation using a previously published scoring system, with each lung given an inflammatory score value from 1 to 4 (higher score indicating greater inflammatory changes in lung) (Wyatt et al., 2020).

To quantify neutrophil and CCR2^+^ inflammatory monocyte‐derived macrophage recruitment, lung sections were stained with anti‐myeloperoxidase (MPO (ab9535); 1:100; Abcam, Cambridge, MA) and anti‐CCR2 (ab273050) 1:100; Abcam. Donkey anti‐rabbit (AlexaFluor488) (A21206) or goat anti‐rat (AF555) (A21434) antibodies (Thermo Fisher, Waltham, MA) or goat anti‐mouse IgG (Alexa Fluor Plus 555 (A32727); Invitrogen, Carlsbad, CA) with DAPI (4′6‐diamindino‐2‐phenylindole) (H‐1200) to identify nuclei (Vector Laboratories, Curlingame, CA) as previously described (Gaurav et al., 2021; Poole, Mikuls, et al., 2022). Photographs (10/lung/mouse) of lung parenchyma were taken under a Zeiss Observer.Z1 fluorescent microscope objective 40X (oil immersion) (Zeiss Observer.Z1 [Zeiss, White Plains, NY]) using a Zeiss AxioCam Mrm camera and ZenPro 2012 software. Images were analyzed using Image J software (RRID:SCR_003070) FIJI 2 (Mikuls et al., 2021; Poole, Mikuls, et al., 2022).

Prior work has demonstrated that repetitive inhaled environmental exposures induce post‐translational changes marked by the production of CIT and MAA‐modified lung proteins, which increase inflammatory lung disease susceptibility (Poole, Mikuls, et al., 2022). Citrullinated (CIT) and malondialdehyde acetaldehyde (MAA) modified proteins and vimentin were stained as previously described (Poole, Mikuls, et al., 2022). Briefly, the following labeling antibodies were utilized: Cy5 rabbit anti‐vimentin (BS‐8533R) (Bioss, Woburn, MA), Zenon AF 594 label (Invitrogen, Carlsbad, CA), rabbit polyclonal IgG antibody to MAA (Poole, Mikuls, et al., 2022), and mouse monoclonal anti‐peptidyl‐citrulline antibody (MABN328) (clone F95 IgMκ, Millipore Sigma, Burlington, MA) with detection of the F95 antibody by AF 488 AffiniPure donkey anti‐mouse IgM, μ chain specific (709–545‐073) (Jackson Immunoresearch, West Grove, PA). All immunohistochemical images were quantified by Image J FIJI plugin (version: 2.9.0/1.53 t), and colocalization was performed using Image J (RRID:SCR_003070) FIJI 2 (Mikuls et al., 2021; Poole, Mikuls, et al., 2022).

### 2.8 RNA preparation and analysis

RNA isolation, analysis, sequencing, pathway analysis, and subsequent data visualization were performed as previously described (Schwab, Nelson, Gleason, et al., 2024). Total RNA from homogenized mouse whole lung tissue was isolated with an RNeasy Mini Kit (74104) per manufacturer's instructions (Qiagen, Germantown, MD). All samples had a A260/280 of 1.8 or above and RNA quality number (RQN) scores >8.0 using Nanodrop (Thermo Scientific, Nanodrop Products, Wilmington, DE) and Advanced Analytical Technical Instrument Fragment Analyzer (AATI, Ames, IA). Libraries were generated from each sample with the NuGEN Universal Plus mRNA‐Seq library kit from TECAN (Redwood City, CA) and were then multiplexed and sequenced on the NextSeq550 Sequencer (Illumina) as previously described (Schwab, Nelson, Gleason, et al., 2024). Differentially expressed genes (DEG) were identified using the R/Bioconductor package DESeq2 (Love et al., 2014). Reads were mapped to the mm10 (GRCm38) mouse reference genome. The resulting p values from each comparison were adjusted for false discovery rate (FDR) using the Benjamini‐Hochberg (B‐H) method (Benjamini & Hochberg, 1995). The heatmap was plotted by pheatmap 1.0.12 package in R 4.0.3 based on the value of log_2_(TMP_value+0.0001) for all significant genes (adj *p* < 0.05). The heatmap then underwent symmetric normalization to improve comparative visualization. The volcano plot was created using the GraphPad Prism software. Gene enrichment analyses were performed using Ingenuity Pathway Analysis (IPA; Qiagen Inc., https://www.qiagenbioinformatics.com/products/ingenuity‐pathway‐analysis). The R package *GOPlot* was utilized to visualize the relationship between genes and enriched pathways. The datasets have been deposited to the Gene Expression Omnibus (GEO) database with access number GSE279990.

### 2.9 Micro‐CT imaging

Lung density was determined using a live animal Quantum GX‐2 micro‐CT scanner (Perkin Elmer, Waltham, MA) at baseline and 5 and 8 days post‐initial LPS/saline exposure. High‐speed, 4‐minute scans were performed utilizing an X‐ray tube voltage of 90 kV and a current of 88 μA as previously described (Bauer et al., 2024). 3D model construction and quantification were completed using 3D Slicer (5.6.2) software (Fedorov et al., 2012). Registration between baseline and subsequent scans was performed using the Elastix extension (Klein et al., 2010).

### 2.10 Statistical analysis

Sample‐size requirements were extrapolated from previous work assessing IL‐10 treatment after a one‐time LPS exposure (Poole, Gaurav, et al., 2022). With the mean (±SD) lung IL‐6 being 241 pg/mL (±130 pg/mL) (LPS + vehicle) and 12 pg/mL (±2 pg/mL) (LPS + IL‐10), a sample size of *n* = 5 in each group would achieve 80% power at the 0.05 level of significance. Here, the sample size per treatment group is: *n* = 5 (Saline‐exposed groups) and *n* = 10 (LPS‐exposed groups). Numbers less than the maximum number reflect limitations in the available sample quantity or quality. The Shapiro–Wilk test was utilized to test for normality among treatment groups. If the normality condition was satisfied, a one‐way ANOVA test was used, and if not satisfied, a Kruskal–Wallis test was used instead. Subsequent utilization of the two‐stage Benjamini, Krieger, & Yekutieli procedure for controlling the false discovery rate was used to assess differences between any two groups. All statistical analyses were performed using GraphPad Prism (version: 10.2.2) software, and statistical significance was accepted at a *p* value <0.05.

## 3 RESULTS

### 3.1 Lung‐targeted IL‐10 therapy reduces LPS‐induced indices of systemic inflammation

**FIGURE 1:**
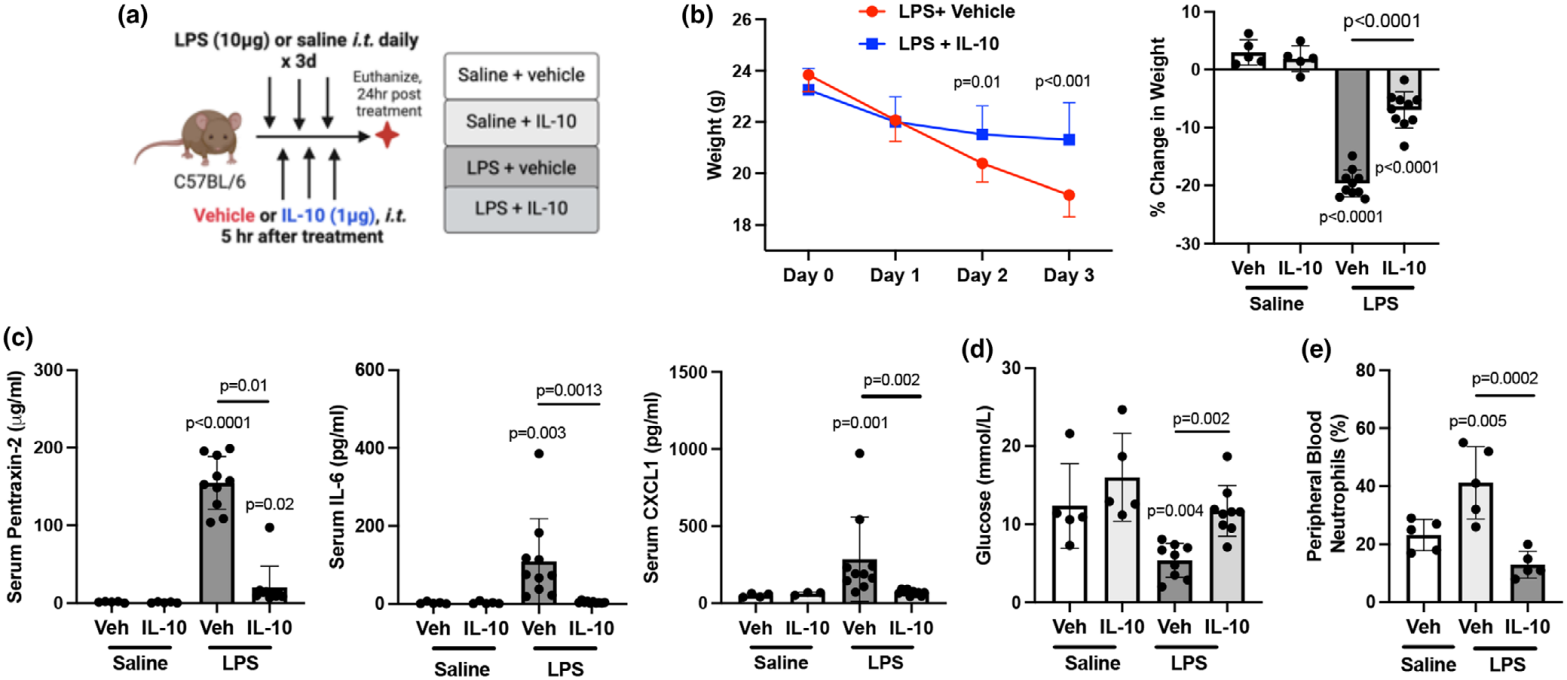
Lung‐delivered IL‐10 administered after LPS exposures reduces repeated LPS‐induced weight loss and systemic inflammatory responses. (a) Mice were treated with LPS or saline daily for 3 days and received either treatment with IL‐10 (10 μg) or vehicle (veh) daily for 3 days administered 5 h after LPS or saline and were euthanized at 24 h after the final treatment. (b) Line graph depicts weights over time with scatter dot plot demonstrating percent change in weight upon study completion across indicated groups. (c) Acute phase protein pentraxin‐2, IL‐6, and neutrophil chemoattractant CXCL were quantified in serum across indicated groups. (d) Blood glucose levels and (e) peripheral blood neutrophils by percentage were quantified across indicated groups. All data expressed as mean with SD bars of *n* = 5 mice/saline exposed groups; *n* ≥ 8 mice/LPS exposed groups. Statistical significance by *p*‐values versus Sal+Veh (no line) or denoted between groups by line.

Mice were challenged with LPS followed by direct lung‐delivered IL‐10 daily for 3 days and were sacrificed the following day (Figure 1a). Treatment with IL‐10 reduced LPS‐induced weight loss at days 2 and 3 relative to vehicle‐treated animals by 49% and 65%, respectively (Figure 1b).

Serum levels of pentraxin‐2 (acute phase reactant), IL‐6, and CXCL1 induced by LPS were reduced by 87%, 96%, and 76%, respectively, with IL‐10 treatment (Figure 1c). Additionally, IL‐10 blunted decreases in serum glucose induced by LPS (Figure 1d) with no effect on other blood chemistry indices (Table S2). IL‐10 also reversed LPS‐induced increases in percent blood neutrophils (Figure 1e) and decreases in percent lymphocytes, with no change in percent monocytes (Table S3).

### 3.2 IL‐10 treatment strikingly decreases several LPS‐induced airway and lung inflammatory, and wound repair mediators

**FIGURE 2:**
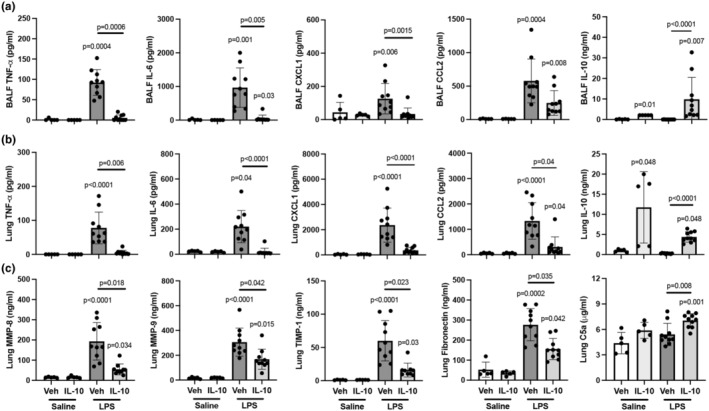
LPS‐induced airway and lung tissue inflammatory mediator release is reduced by treatment with localized, lung‐delivered IL‐10. (a) Bronchoalveolar lavage fluid (BALF) and (b) lung tissue homogenates utilized to quantify levels of inflammatory cytokines (TNF‐⍺, IL‐6), chemokines (CXCL1, CCL2), and IL‐10 across indicated groups. (c) Quantification of lung levels of extracellular matrix proteins (MMP‐8, MMP‐9, TIMP‐1), fibronectin, and complement component C5a across indicated groups. All data are expressed in scatter dot plots with mean and SD bars of *n* = 5 mice/saline exposed groups; *n* = 10 mice/LPS exposed groups. Statistical significance is denoted by *p*‐values versus Sal+Veh (no line) or denoted between groups by line.

To characterize the effect of IL‐10 therapy on modulating lung inflammatory and wound repair processes, BALF and lung tissues were assessed. IL‐10 treatment significantly decreased BALF levels of LPS‐induced TNF‐α (*p* = 0.0006), IL‐6 (*p* = 0.005), CXCL1 (*p* = 0.0015), but not CCL2 (*p* = 0.094) by 94%, 95%, 73%, and 57%, respectively (Figure 2a). Correspondingly, IL‐10 treatment also significantly reduced LPS‐induced lung tissue levels of TNF‐α (*p* = 0.006), IL‐6 (*p* < 0.0001), CXCL1 (<0.0001), and CCL2 (*p* = 0.04) by 93%, 92%, 85%, and 77%, respectively (Figure 2b). IL‐10‐treated animals exhibited increased IL‐10 levels in both BALF and lung homogenates (Figure 2a,b). LPS‐induced lung tissue levels of MMP‐8 (*p* = 0.018), MMP‐9 (*p* = 0.042), TIMP‐1 (*p* = 0.023), and fibronectin (*p* = 0.035) were significantly decreased by 72%, 45%, 73%, and 44%, respectively, with IL‐10 (Figure 2c). Lung C5a levels were elevated by 23% (*p* = 0.008) in the LPS + IL‐10 relative to LPS + vehicle (Figure 2c). IL‐10 treatment did not reduce LPS‐induced lung levels of CCL7, MMP‐3, or TGF‐β (Table S4).

### 3.3 IL‐10 treatment reduced LPS‐induced airway hyperresponsiveness and improved lung compliance

**FIGURE 3:**
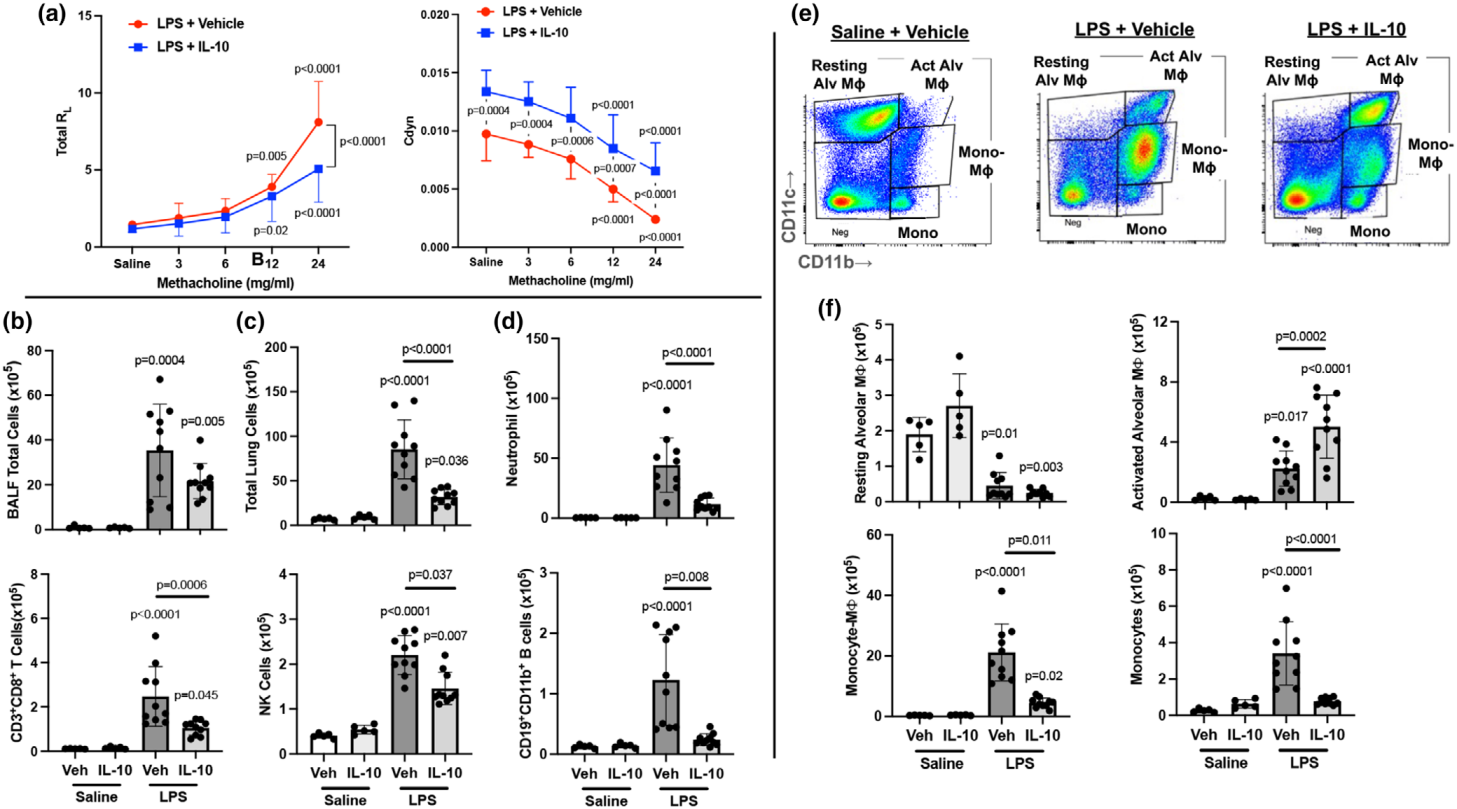
Localized, lung‐administered IL‐10 inhibits LPS‐induced lung dysfunction and lung cellular infiltrates. (a) Airway hyper‐responsiveness expressed as total lung resistance (R_L_) and dynamic compliance (Cdyn) were measured following escalating doses of aerosolized methacholine between LPS‐exposed mice treated with IL‐10 versus vehicle. (b) BALF total cell numbers across groups. (c) Total lung cells of mice exposed to LPS or saline and treated with IL‐10 versus vehicle control were enumerated. (d) Lung immune cell infiltrates were determined by flow cytometry on live CD45^+^ cells after exclusion of debris and doublets across indicated groups, with lung cell % population in respective gate multiplied by total lung cells. Cells were defined as CD11c^−^Ly6G^+^ neutrophils, CD3^+^CD8^+^ T cells, natural killer (NK) cells, and innate immune CD19^+^CD11b^+^ B cells. Gating strategy is depicted in *Figure E1/online repository*. (e) Gating strategy of monocyte–macrophage (Mɸ) subpopulations based upon CD11c and CD11b expression (f) Monocyte‐Mɸ infiltrates are depicted across treatment groups and defined as resting alveolar Mɸ (CD11c^+^CD11b^−^), activated alveolar Mɸ (CD11c^+^CD11b^+^), recruited/transitional monocyte/Mɸ (CD11c^int^CD11b^+^), and monocytes (CD11c^−^CD11b^+^). All data are expressed in scatter dot plots with mean and SD bars of *n* = 5 mice/saline‐exposed groups; *n* = 10 mice/LPS‐exposed groups. Statistical significance is denoted by *p*‐values versus Sal+Veh (no line) or denoted between groups by line.

LPS‐induced airway hyperresponsiveness as measured by lung resistance was decreased with IL‐10 (vs. vehicle) treatment at the 24 mg/mL methacholine dose (*p* < 0.0001) (Figure 3a). LPS‐associated lung dynamic compliance was increased with IL‐10 (vs. vehicle) at baseline (*p* = 0.0004) and at all methacholine doses (3 mg/mL: *p* = 0.0004; 6 mg/mL: 0.0006; 12 mg/mL: *p* = 0.0007, and 24 mg/mL: *p* < 0.0001) (Figure 3a).

### 3.4 IL‐10 treatment mitigates LPS‐induced lung cellular infiltrates

LPS‐induced airway influx of total cells, neutrophils, macrophages, and lymphocytes was not significantly reduced in the lavage fluid with IL‐10 treatment (Figure 3b and Table S4). Lung immune cell profiling was characterized by flow cytometry. IL‐10 treatment (vs. vehicle) decreased LPS‐induced total lung cell infiltrates by 63% (Figure 3c). Furthermore, IL‐10 treatment reduced LPS‐induced recruitment of neutrophils (74%), CD8^+^ T cells (57%), NK cells (34%), and CD19^+^CD11b^+^ innate immune B cells (80%) but not CD3^+^CD4^+^ T cells or CD19^+^CD11b^−^ B cells (Figure 3d and Table S4). Four monocyte/macrophage (Mɸ) subpopulations gated by CD11c and CD11b expression (Figure 3e) were defined as resting alveolar Mɸ (CD11c^+^CD11b^−^), activated alveolar Mɸ (CD11c^+^CD11b^+^), transitioning/recruited monocyte‐Mɸ (CD11c^int^CD11b^+^) and monocytes (CD11c^−^CD11b^+^). There was a significant increase in LPS‐induced activated alveolar Mɸ (+55%) with IL‐10 treatment with a corresponding decrease in resting alveolar Mɸ. IL‐10 robustly reduced LPS‐induced recruited monocyte‐Mɸ and monocyte subpopulations by 79% and 77%, respectively (Figure 3f).

### 3.5 LPS‐induced adverse lung histopathology marked by neutrophil and CCR2 ^+^ monocyte/Mɸ infiltrates is decreased with IL‐10 treatment

**FIGURE 4:**
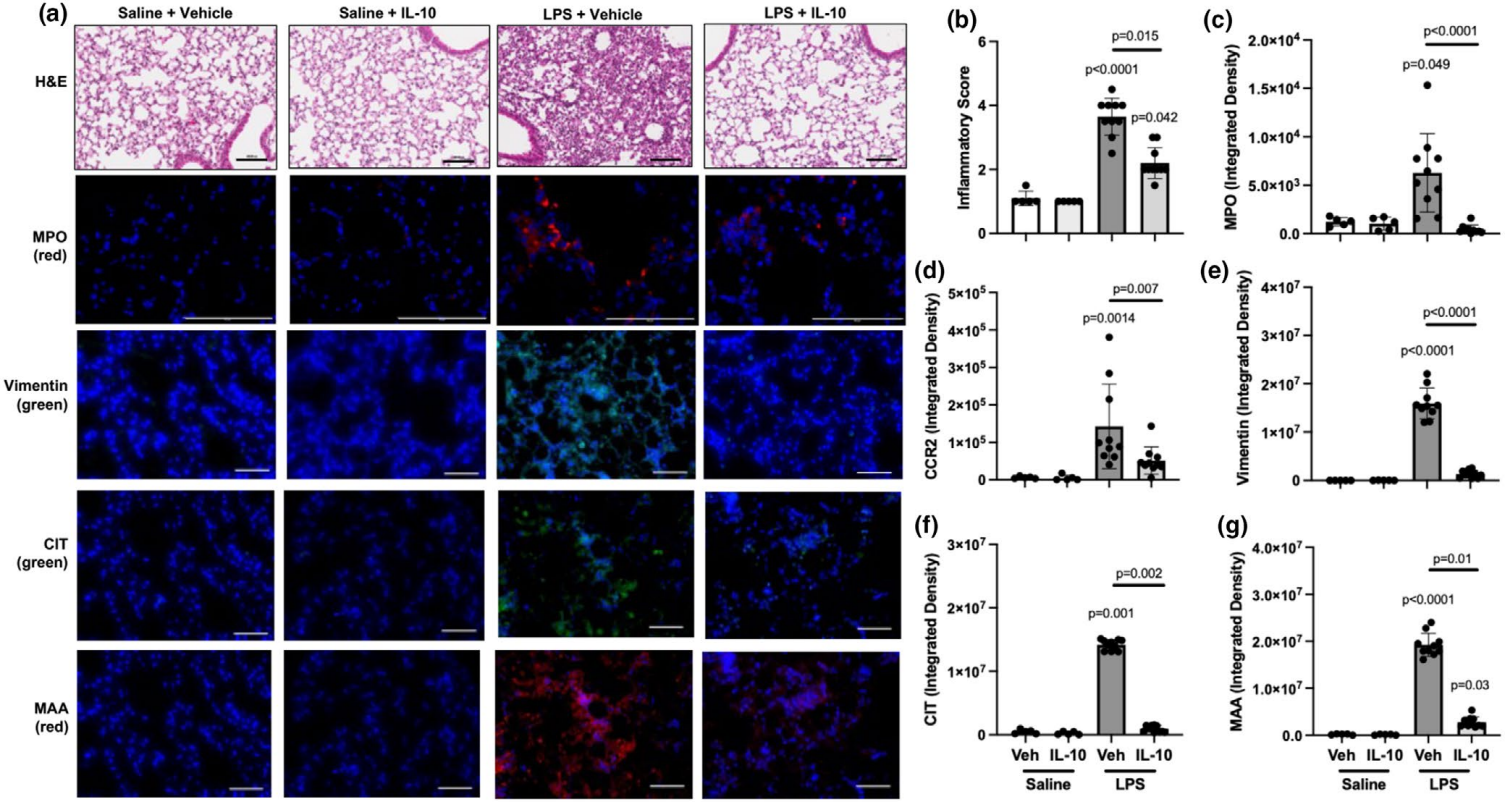
Repeated LPS‐exposure induced adverse lung histopathology, neutrophil and CCR2^+^ cell infiltrates, vimentin accumulation and expression of post‐translationally modified proteins are inhibited with lung‐administered IL‐10 therapy. (a) Representative lung sections for indicated groups stained for H&E, myeloperoxidase (MPO, red), CCR2 (red), vimentin (green), citrulline (CIT, green), and malondialdehyde‐acetaldehyde (MAA, red)‐adducted proteins with DAPI nuclei staining (blue) by confocal microscopy. Line scale denotes 100 μm. (b) Semi‐quantitative inflammatory score across indicated groups. (c–g) Integrated density of MPO, CCR2, vimentin, CIT‐ and MAA‐modified proteins quantified per each mouse. All data expressed in scatter dot plots with mean and SD bars of *n* = 5 mice/saline‐groups; *n* = 10 mice/LPS‐groups. Statistical significance denoted by *p*‐values versus Sal+Veh (no line) or denoted between groups by line.

IL‐10 treatment favorably reduced LPS‐induced adverse lung histopathological changes (Figure 4a–d). Semi‐quantitative assessment of H&E‐stained lung sections demonstrated a 40% reduction in LPS‐induced bronchiolar and alveolar inflammation with IL‐10 treatment (Figure 4a,b). Moreover, LPS‐induced MPO^+^ neutrophils and CCR2^+^ monocyte/Mɸ were reduced by 93% and 64%, respectively, with IL‐10 therapy (Figure 4a,c,d).

### 3.6 IL‐10 treatment strikingly reduced LPS‐induced vimentin accumulation and the expression of post‐translationally modified proteins

LPS‐induced vimentin expression, an extracellular matrix protein with expression attributable to mesenchymal cells and macrophages, was significantly reduced by 92% (*p* < 0.0001) with IL‐10 treatment (Figure 4a,e). Additionally, LPS‐induced high expression of CIT‐ and MAA‐modified proteins, and IL‐10 treatment strikingly reduced LPS‐induced lung CIT and MAA expression by 93% and 86%, respectively (Figure 4a,f,g).

### 3.7 Lung‐delivered IL‐10 modulates the LPS‐altered lung transcriptome

**FIGURE 5:**
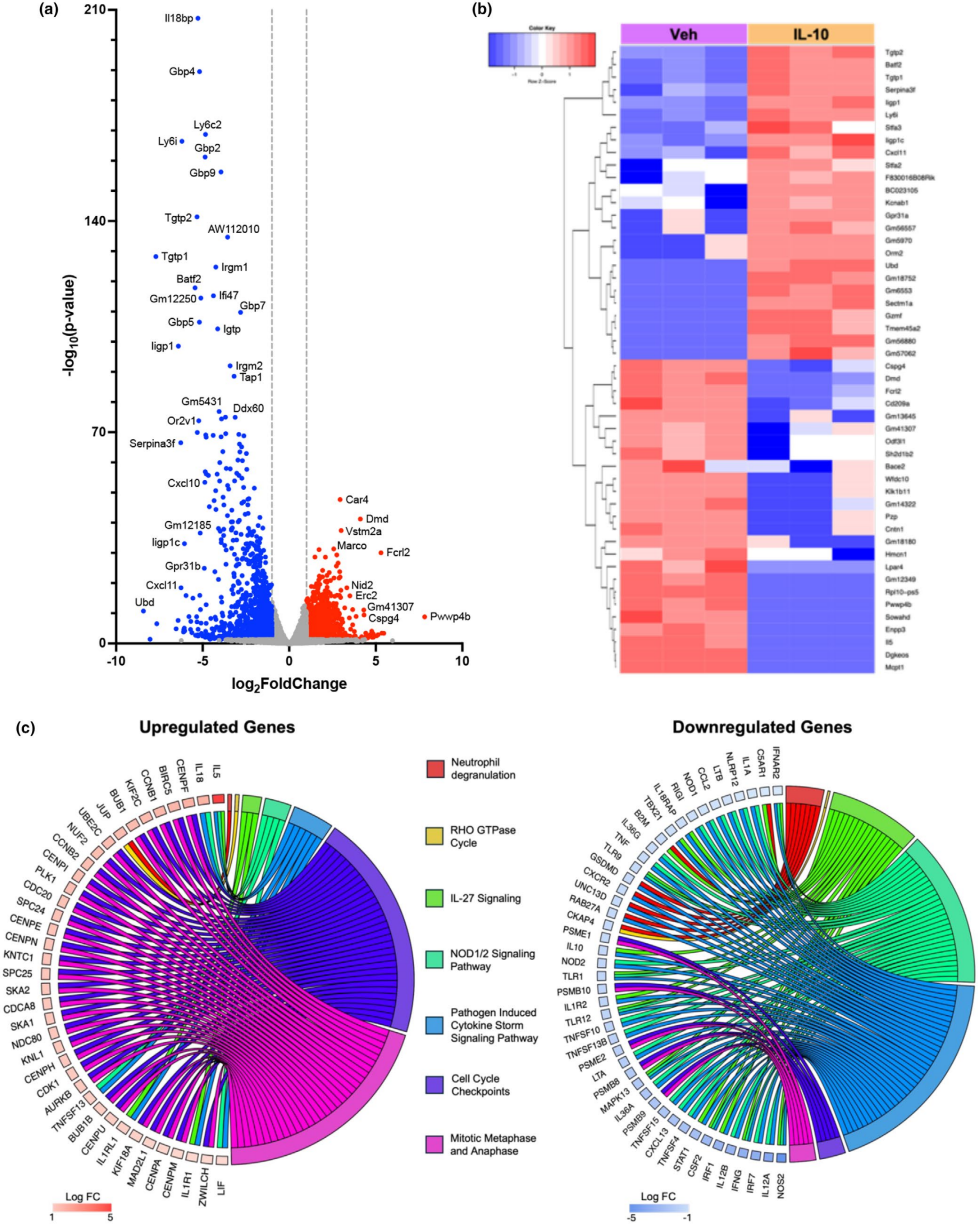
Localized IL‐10 therapy modulates transcriptome of lung tissue following repeated LPS exposure. (a) Volcano plot demonstrates statistical significance (−log10 (*p*‐value)) versus magnitude of change (log_2_ fold change) in expression of specified gene transcripts (−log10 (*p*‐value) >1.3) with red reflecting upregulated and blue reflecting downregulated genes. (b) Heatmap demonstrates hierarchical clustering of the 25 most upregulated and downregulated genes and relative frequencies of genes subjected to symmetric normalization of log2 (transcripts per million [TPM] + 0.0001) for all significant genes (adj *p* < 0.05) to avoid any nonsense values. The color scheme represents symmetric normalization of relative frequencies from high expression (red) to low expression (blue). (c) Chord plots demonstrate the top canonical pathways of the whole lung transcriptome based on Ingenuity Pathway Analysis (IPA) output and the corresponding fold change for the top upregulated and downregulated genes (*p* < 0.05) associated with each modulated pathway. *n* = 3 mice/treatment group.

Lung transcriptomic analysis identified differentially expressed genes and pathways modulated by IL‐10 (vs. vehicle) therapy in LPS‐treated mice. Of the 18,710 genes identified by bulk sequencing, 2082 genes were significantly (*p*_adj_ < 0.05) modulated, with 979 genes upregulated and 1103 genes downregulated (Appendix S1 and S2). High‐expressing genes by log_2_foldchange are highlighted by volcano plot (Figure 5a). Hierarchical clustering of the top 25 most upregulated and downregulated transcripts is shown in the heatmap (Figure 5b). The top canonical pathways organized by upregulated and downregulated gene transcripts are shown by chord plots (Figure 5c). The top 10 most upregulated and downregulated named genes and their associated functions are included in Table S5.

### 3.8 Therapeutic benefits of IL‐10 therapy with LPS exposure are sustained after 1 week

**FIGURE 6:**
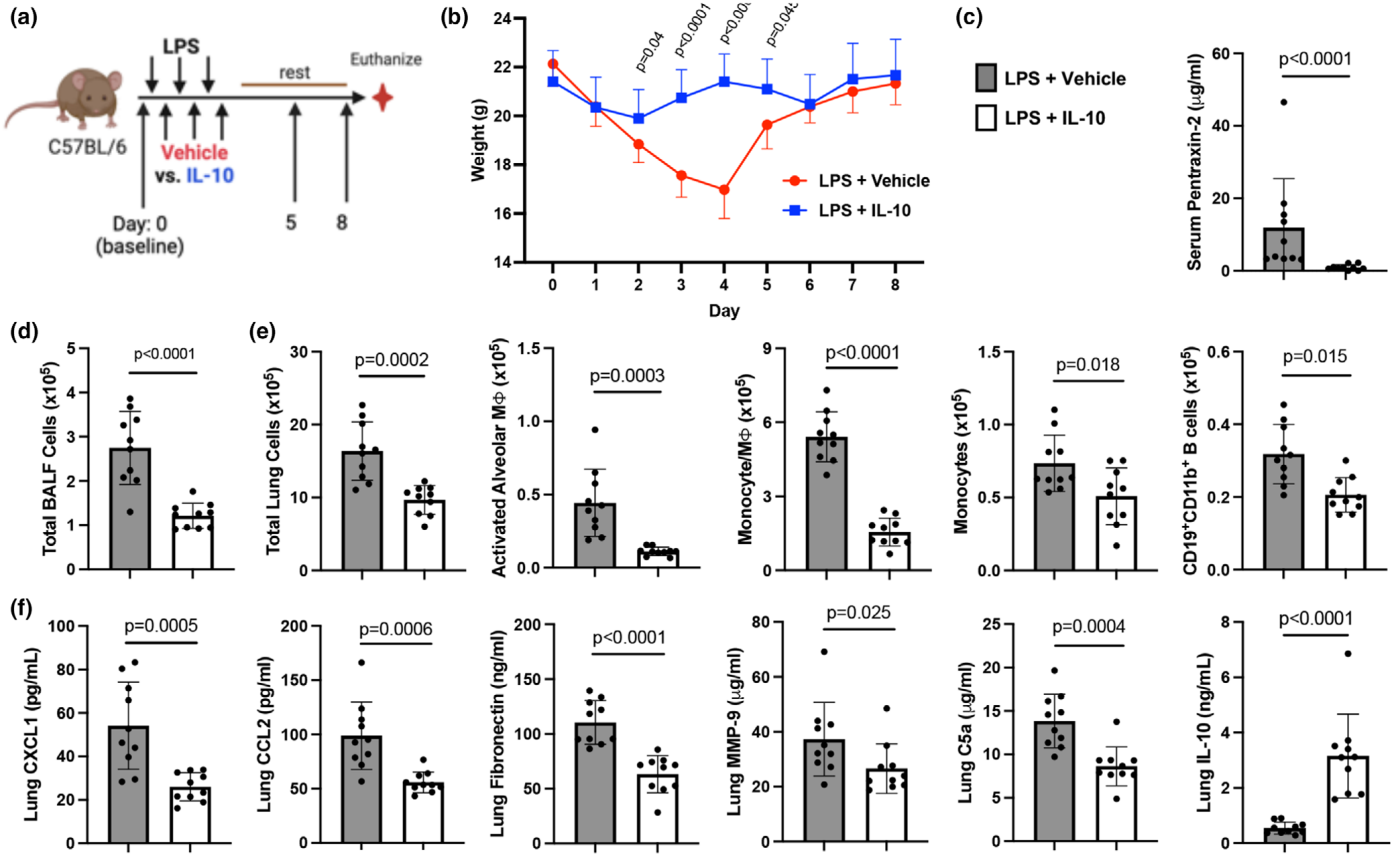
Localized lung IL‐10 therapy inhibition of adverse LPS‐induced experimental endpoints persists without rebound after 1 week. (a) Mice were treated with LPS for 3 days and received either treatment with IL‐10 (10 μg) or vehicle (veh) for 3 days and subsequently rested for 1 week prior to study completion. (b) Line graph depicts weights over time between IL‐10 and vehicle‐treated LPS‐exposed animals. (c) Serum levels of acute phase protein pentraxin‐2 between groups. (d) BALF total cell numbers are reduced with IL‐10 therapy. (e) LPS‐induced lung cell infiltrates including total lung cells, activated macrophages (Mɸ), recruited/transitioning monocytes/Mɸ, monocytes, and innate CD19 + CD11b + B cells remain reduced with IL‐10 versus vehicle treatment. (f) Lung levels of LPS‐induced chemokines (CXCL1, CCL2), fibronectin, matrix metalloproteinase (MMP)‐9, and complement component C5a are reduced with IL‐10 therapy. Lung levels of IL‐10 remain increased with IL‐10 therapy at 1 week. All data expressed as mean with SD bars of *n* = 10 mice/LPS‐exposed groups. Statistical significance denoted by *p*‐values between groups.

To determine whether the benefits of IL‐10 treatment are sustained without rebound or compensatory side effects, separate mice were allowed to recover for 5 days following LPS exposure (Figure 6a). No adverse rebound or compensatory effects were observed. Instead, IL‐10 treatment (vs. vehicle) demonstrated sustained protection against LPS‐induced weight changes, increased levels of serum pentraxin‐2 (*p* < 0.0001), increased total cells in BALF (*p* < 0.0001), and macrophage (*p* = 0.0002) and lymphocyte (*p* = 0.0011) influx after an additional week of observation (Figure 6b–d and Table S6). Furthermore, after an additional week, the protective effects of IL‐10 observed at earlier time points against LPS‐induced increases in total lung cells (*p* = 0.0002), activated Mɸ (*p* = 0.0003), transitioning monocyte/Mɸ (*p* < 0.0001), monocytes (*p* = 0.018), and CD19^+^CD11b^+^ innate immune B cells (*p* = 0.0015) were sustained (Figure 6e). LPS‐induced CXCL1 (*p* = 0.0005), CCL2 (*p* = 0.0006), fibronectin (*p* < 0.0001), MMP‐9 (*p* = 0.025), and complement C5a (*p* = 0.0004) were also all reduced with IL‐10 treatment relative to vehicle (Figure 2f). Lung IL‐10 levels remained increased in LPS + IL‐10 versus LPS + vehicle treated mice (*p* < 0.0001) (Figure 6f). There were no significant differences in resting alveolar Mɸ, B Cells, NK Cells, CD4^+^ T Cells, CD8^+^ T Cells, CCL7, MMP‐3, MMP‐8, TIMP‐1, or TGF‐β (Table S6).

### 3.9 IL‐10 therapy protects against LPS‐induced lung pathology longitudinally

**FIGURE 7:**
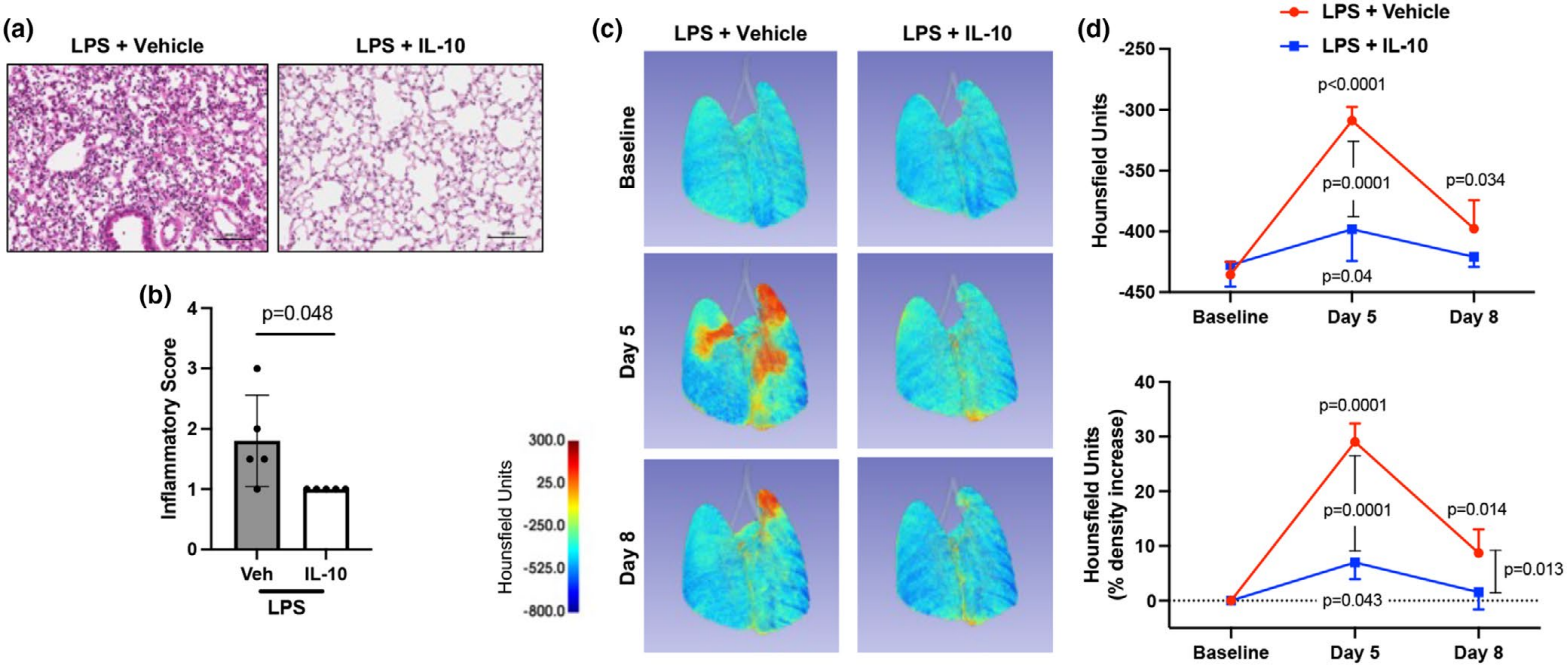
Localized lung IL‐10 therapy protects against LPS‐induced lung inflammatory pathology over time. Mice were treated with LPS for 3 days and received either treatment with IL‐10 (10 μg) or vehicle (veh) for 3 days and subsequently rested for 5 days. (a) Representative H&E images from each treatment group. (b) Scatter dot plot depicts semi‐quantitative lung inflammatory score between groups. (c) Representative micro‐CT image at baseline, day 5, and day 8 of the same mouse with the density of lung tissues demonstrated by a colored range of Hounsfield units. (d) Line graph of the mean with SD bar of all mice depicted by averaged Hounsfield units per mouse (top graph) and percent change to baseline per mouse (bottom graph). All data are expressed as mean with SD bars of *n* = 5 mice/group. Statistical significance is denoted by *p*‐values between groups.

To further confirm the favorable effect of IL‐10 treatment in reducing lung pathologic changes, lung tissues were analysed at 5 days (Day 8) post‐exposure with longitudinal live animal micro‐CT imaging. IL‐10‐treated, LPS‐exposed animals exhibited decreased lung inflammation compared to vehicle (*p* = 0.048) (Figure 7a,b). CT imaging demonstrated decreased lung inflammatory changes with IL‐10 treatment marked by decreases in LPS‐induced lung density represented by Hounsfield units, with significance noted at day 5 between groups by actual units (*p* = 0.0001) and percent density increase (*p* = 0.0001) and at day 8 by percent density increase (*p* = 0.013) (Figure 7c,d).

## 4 DISCUSSION

Responsible for more than 12 million deaths annually, environmental and occupational exposure‐induced lung diseases represent a major global health concern (Eckhardt & Wu, 2021). Beyond exposure mitigation and supportive care approaches applied post‐exposure, therapeutic approaches to reduce the development and progression of chronic environmental lung diseases are lacking (Goldman & Schafer, 2012). Previous studies demonstrated that lung‐targeted IL‐10 treatment reduced TNF‐⍺ and IL‐6 release as well as the recruitment of lung monocytes‐macrophages, and neutrophils following a one‐time exposure to LPS (Poole, Gaurav, et al., 2022). Here, preclinical animal studies comprehensively inform the potential translatability of lung‐delivered IL‐10 treatment following repeated exposures to occupational and/or environmental exposures enriched in endotoxin. Specifically, lung‐delivered IL‐10 treatment mitigated LPS‐induced weight loss and airway hyperresponsiveness and improved lung compliance. IL‐10 also reduced LPS‐induced lung and systemic pro‐inflammatory cytokines/chemokines, wound repair mediators, inflammatory lung cell recruitment, lung inflammatory pathology, lung vimentin deposition, and post‐translationally modified protein expression. These beneficial effects persisted without rebound or untoward compensatory effects following the discontinuation of therapy. These findings would also support future studies investigating the application of lung‐targeted IL‐10 after high‐consequence, inflammatory hazard exposures for potential therapeutic benefit for exposed persons.

Altered lung function is a hallmark of lung inflammation (Hakansson et al., 2012). Low dynamic compliance is characteristically associated with both chronic obstructive pulmonary disease and restrictive lung diseases, commonly the result of extensive lung tissue remodeling following lung injury/inflammation (Brandenberger & Muhlfeld, 2017; Papandrinopoulou et al., 2012; Plantier et al., 2018). Airway hyperresponsiveness (AHR), a canonical feature of allergic asthma, can also be induced by exposure to non‐allergic agents, including ozone, viruses, LPS, inorganic and organic dust extracts, and chemical toxicants (Albright et al., 2022; Brass et al., 2012; Hollingsworth 2nd et al., 2004; Marzec & Nadadur, 2024; Schwartz et al., 2001; Sundblad et al., 2009). Here, IL‐10 treatment improved both LPS‐associated AHR and lung compliance indices. Corroborating the functional effect on respiratory compliance, IL‐10 treatment reduced repeated LPS‐induced lung density, as characterized by longitudinal micro‐CT imaging studies. In general, therapies aimed at reversing non‐allergic respiratory dysfunction are limited, emphasizing the potential significance of lung‐directed IL‐10 therapy to mitigate LPS‐induced AHR and adverse lung compliance. Potential mechanisms explaining our findings are likely multifactorial. Inflammatory mediators (e.g., TNF‐⍺ and IL‐6), neutrophils, and lymphocytes (e.g., CD8^+^ effector T cells), and mediators of tissue remodeling and wound repair (e.g., MMPs, vimentin, fibronectin) as identified in this study have all been implicated by others to provoke airway symptoms that can contribute to bronchoconstriction and AHR as well as aberrant lung compliance (Hinks et al., 2019; Hough et al., 2020; Schwab & Poole, 2023; Varricchi et al., 2022). Lung‐directed IL‐10 treatment strikingly reduced most of these cellular and protein mediators induced by repeated LPS exposures.

A critical target explaining the mechanistic action of IL‐10 is in its ability to regulate the proinflammatory cytokine TNF‐α. Even though IL‐10 was administered after LPS exposure, IL‐10 significantly attenuated both TNF‐α expression in the lung tissue and airway compartments. The IL‐10/IL‐10R‐α signaling cascade is mediated by the Jak1/Tyk2/STAT3 pathway (Carlini et al., 2023), which leads to inhibition of TNF‐α release by inhibiting TNF‐α converting enzyme (ADAM‐17) activity via the induction of protein kinase C zeta (Wyatt et al., 2020). Among many activities, TNF‐α elicits positive, feedforward mechanisms that include upregulation of MMPs through the c‐Src, MAPKs, and NF‐kB pathways (Lee et al., 2014; Lehmann et al., 2005). Whereas TNF‐α‐overexpressing mice possess a lung phenotype with emphysematous and fibrotic features, therapies targeting only TNF‐α have not shown benefits due to both infectious and noninfectious complications resulting from TNF‐α inhibitor‐related pulmonary toxicity, especially in the context of pulmonary fibrosis (Khasnis & Calabrese, 2010; Lundblad et al., 2005).

Through its ability to inhibit the release of pro‐inflammatory mediators and chemokines, lung‐targeted IL‐10 therapy reduced LPS‐induced recruitment of monocyte/macrophage subpopulations and neutrophils. Recruited lung monocyte‐macrophages from the circulation are recognized as critical drivers of lung pathology, facilitating the transition of acute lung inflammation to wound repair and/or lung fibrosis (Misharin et al., 2017). Moreover, in the earlier stages of acute lung injury, depletion of neutrophils is therapeutically beneficial, given that neutrophils release cytotoxic molecules responsible for tissue damage (Blazquez‐Prieto et al., 2018). Additionally, decreases in the LPS‐induced recruitment of CD8^+^ T cells, NK cells, and innate‐like B1 B cells (CD19^+^CD11b^+^ B cells) were also observed with IL‐10 treatment, cells that regulate local immune responses. IL‐10 limits the intracellular production of chemokines but also inhibits their secretion into the extracellular space (Carlini et al., 2023; Ouyang & O'Garra, 2019). The resultant effect of IL‐10‐mitigated chemokine release is an observable decrease in leukocyte infiltration, as we have demonstrated in these current studies despite repeated exposure to LPS. Thus, limiting the infiltration of these immune cells to the lung, particularly monocyte/macrophage subpopulations that are long‐lasting, may explain the potential beneficial application of IL‐10. Moreover, this would suggest that IL‐10 translatable approaches would need to be applied soon after adverse inhalant exposures.

Transcriptomic studies were conducted to elucidate potential mechanisms of direct IL‐10 therapy in the context of LPS exposure, aiming to guide further research and support the development of novel precision medicine approaches. In these bulk RNA sequencing studies, IL‐10 treatment notably significantly (by −log_10_ *p*‐value) upregulated *Marco*, *Car4*, and *Dmd*. Scavenger receptor MARCO is expressed on macrophages and mediates binding and ingestion of unopsonized environmental particles, including nano‐sized materials and exosomes (Kanno et al., 2020). *Car4* has been implicated in the regeneration of pulmonary vasculature by helping prime endothelial cells to receive reparative signals (Niethamer et al., 2020). *Dmd* encodes dystrophin, and research exploring its potential role in lung injury and repair (beyond muscle weakness) indicates its crucial role in lung barrier and epithelial cell function (Morici et al., 2016; Tyagi et al., 2022). Additionally, IL‐10 downregulated several genes (i.e., *Tgtp1*, *Sectm1a*, *Serpina3f*, and *Cxcl11*) that perpetuate inflammatory events (Callahan et al., 2021; de Mezer et al., 2023; Kamata et al., 2016; Martens et al., 2004). However, it should be noted that the time point interrogated for gene expression was 2 days following the last LPS exposure and may have missed optimal gene expression times for other classic inflammatory and pro‐fibrotic pathways (e.g., IL‐1β, TGF‐β, PDGF, VEGF, granulin). Pathway analysis of differentially expressed genes demonstrated increases in genes associated with repair and regeneration pathways and reductions in injury/inflammatory genes, including neutrophil degranulation, IL‐27, NOD1/2, and cytokine storm signaling pathways. Given that increases in IL‐27 have been correlated with inflammatory reactivity and disease severity in acute lung injury/acute respiratory distress syndrome, the feedback regulation provided by IL‐10 likely contributes to its therapeutic effects (Xu et al., 2013). NOD2 is a known positive regulator of IL‐10 production, so feedback inhibition by exogenously supplied IL‐10, in part, explains the downregulation of genes associated with inflammasome (NOD) signaling (Chaput et al., 2013; Jostins et al., 2012; Trindade & Chen, 2020).

There are limitations to this study. Whereas high‐concentration LPS can be used to model high‐consequence environmental events in which IL‐10 therapy may be of therapeutic importance, other exposure models should be considered, including microbial infections, inorganic dusts, and industrial and chemical toxicants. In addition, the prototypical LPS molecule used in this current study was derived from *E. coli*, and because LPS derived from other gram‐negative bacteria may have variations in their sugar chains that could potentially impact biological responses, future studies could investigate other airborne endotoxins. Although IL‐10 therapy does not impact monocyte/macrophage and neutrophil phagocytic ability or the production of reactive oxygen species (suggesting the retention of key innate immune functions (Schwab, Wyatt, Nelson, et al., 2024)), future studies to understand its role in infection would inform whether antimicrobial prophylaxis would be required for translational application. Moreover, we did not investigate lung dendritic cells (DCs), and although lung DC numbers are limited in the lungs of mice (Liu et al., 2017), future studies should delineate the effect of IL‐10 therapy on the various murine lung DC subpopulations including conventional DCs, plasmacytoid DCs, and monocyte‐derived DCs (Liu et al., 2017). Although IL‐10 has a short half‐life (<4–6 h) (Minshawi et al., 2020), our studies demonstrated that IL‐10 levels remained increased after 5 days in the context of LPS + IL‐10, and thus, further time course studies may be warranted as inflammatory events may impact half‐life and/or enhance IL‐10 production. Notably, there were initial increases in activated alveolar macrophages (CD11c^+^CD11b^+^) and lung C5a levels that resolved by 1 week in the LPS + IL‐10 (vs. vehicle) treatment group. Both C5a and activated macrophages have beneficial effects in early lung injury by facilitating the clearance of tissue debris and promoting the resolution of local inflammation (Yan & Gao, 2012). Although circulating blood counts and serum chemistry studies were reassuring, suggesting that potential adverse effects may be negated by short‐term use applications, additional investigations may still be warranted.

## 5 CONCLUSION

Overall, these findings lay a strong foundation for the potential clinical translatability of utilizing lung‐targeted IL‐10 therapy as a countermeasure against extreme occupational and/or environmental inflammatory agents. In particular, these occupational environments include those enriched in endotoxin, including sectors of farming, textile, waste treatment, recycling, biotech food production, and processing industries.

## FUNDING INFORMATION

The main funding support is from a grant by the National Institute of Occupational Safety and Health (R01OH012045; JAP). Funding is also from the Department of Defense, PR200793 (JAP, TRM). Other funding support includes VA (BLR&D Merits I01 BX004660 and I01 BX003635, TRM), National Institute of Health (2U54GM115458, TRM), and Rheumatology Research Foundation (TRM). TAW is the recipient of a Research Career Scientist Award (IK6 BX005962). ADS was supported by NIH grant 1F30ES036063–01 through the National Institute of Environmental Health Sciences (NIEHS).

## CONFLICT OF INTEREST STATEMENT

JAP has received research reagents (anti‐IL‐33/ST2 blocking antibody reagent, no monies) from AstraZeneca. JAP is a site recruiter for clinical industry studies for asthma, sinus disease, and urticaria (GlaxoSmithKline, AstraZeneca, Regeneron Pharmaceuticals, CellDex Therapeutics). TRM has consulted for Horizon Therapeutics, Otaltech Therapeutics, Pfizer, and UCB and receives research support from Horizon.

## ETHICS STATEMENT

Neither human participants, data, nor tissues were used in these experiments. The study was conducted and reported in accordance with ARRIVE guidelines (https://arriveguidelines.org). All animal procedures were approved by the University of Nebraska Medical Center (UNMC) Institutional Animal Care and Use Committee and were in accordance with NIH guidelines for the use of rodents.

## Supporting information


Figure S1.

Appendix S1.

Appendix S2.

Table S1.

## Data Availability

Source data for this study are openly available at https://doi.org/10.5281/zenodo.14151997. The transcriptomic datasets have been deposited in the Gene Expression Omnibus (GEO) database with access number GSE279990.
