# Supplementary material for: Lung‐delivered IL‐10 mitigates Lung inflammation induced by repeated endotoxin exposures in male mice
Source: Physiol Rep. 2025 Feb 20;13(4):e70253. doi: 10.14814/phy2.70253 (PMC11842461; doi:10.14814/phy2.70253)
Supplement: Supplementary file 1 — Figure S1. [file PHY2-13-e70253-s003.zip › Figure S1 caption.docx]

Figure S1. Gating strategy for identification of non-debris, singlets, live CD45^+^ cells. A representative control (Sham) mouse is shown. For flow analysis, all panels were first gated as forward scatter-area (FSC-A) x side scatter area (SSC-A) to omit debris, dead or apoptotic cells. This was followed by two single cell gates to omit doublets (FSC-A x FSC-height (H) and SSC-A x SSC-H), followed by live/dead gate and then CD45 to assure removal of any additional dead or apoptotic cells and non-leukocytes. The CD45^+^ cells were placed on CD11c x Ly6G to select neutrophils. A non-neutrophil gate was utilized to select CD19^+^ B cells, and then a non-B cell gate was utilized to select monocyte–macrophage cells based upon CD11c and CD11b expression. A negative CD11c and CD11b gate was utilized to select lymphocyte subpopulations based upon CD3, CD4, CD8, and NK1.1 expression.
