# Supplementary figures and images for: Lung‐delivered IL‐10 mitigates Lung inflammation induced by repeated endotoxin exposures in male mice
Source: Physiol Rep. 2025 Feb 20;13(4):e70253. doi: 10.14814/phy2.70253 (PMC11842461; doi:10.14814/phy2.70253)

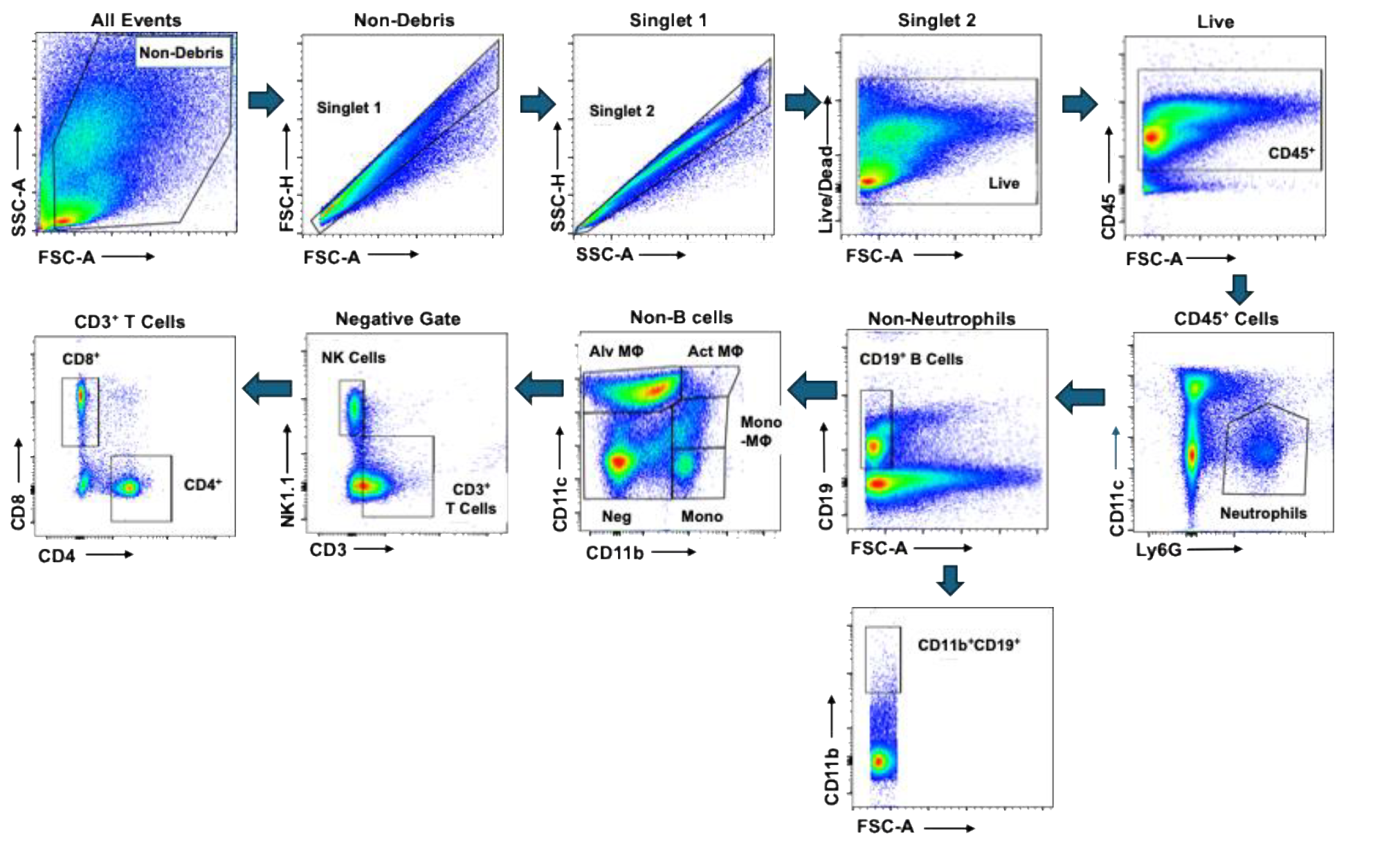

Supplement: Supplementary file 1 — Figure S1. [file PHY2-13-e70253-s003.zip › PHYSREP-2024-12-843-T-f08-z-.tif]
