# Supplementary material for: Lung‐delivered IL‐10 mitigates Lung inflammation induced by repeated endotoxin exposures in male mice
Source: Physiol Rep. 2025 Feb 20;13(4):e70253. doi: 10.14814/phy2.70253 (PMC11842461; doi:10.14814/phy2.70253)
Supplement: Supplementary file 2 — Appendix S1. [file PHY2-13-e70253-s001.docx]

| **IL-10 vs. Vehicle Treatment in LPS-Exposed Mice** | | |
| --- | --- | --- |
| **Upregulated, adjusted p-value <0.05** | | |
| **Gene name** | **log2FoldChange** | **p-value adj** |
| Pwwp4b | 7.822434786 | 4.23E-08 |
| Gm14322 | 5.462149759 | 0.003456655 |
| Rpl10-ps5 | 5.352584591 | 0.003153039 |
| Fcrl2 | 5.305792326 | 1.26E-28 |
| Il5 | 5.131434081 | 0.008192376 |
| Dgkeos | 5.049381865 | 0.017116583 |
| Lpar4 | 4.864419858 | 0.037299286 |
| Sh2d1b2 | 4.850324947 | 0.004813242 |
| Sowahd | 4.799607954 | 0.040244619 |
| Odf3l1 | 4.767261966 | 0.000816408 |
| Klk1b11 | 4.669912866 | 0.009093737 |
| Gm12349 | 4.548514034 | 0.044738642 |
| Wfdc10 | 4.49296761 | 0.012732586 |
| Mcpt1 | 4.451475581 | 0.048966674 |
| Gm13645 | 4.422891292 | 0.002995418 |
| Enpp3 | 4.360394324 | 0.045272259 |
| Cspg4 | 4.324934607 | 1.13E-08 |
| Gm41307 | 4.304785562 | 3.16E-10 |
| Cntn1 | 4.187018283 | 0.029047414 |
| Dmd | 4.10775541 | 1.41E-39 |
| Cd209a | 3.864182807 | 3.15E-08 |
| Gm18180 | 3.821816016 | 0.020159478 |
| Pzp | 3.783258503 | 0.018982415 |
| Hmcn1 | 3.767615937 | 0.000956609 |
| Bace2 | 3.76459461 | 0.040074382 |
| Gm11451 | 3.682897932 | 0.03942483 |
| Fgf11 | 3.66586639 | 1.06E-05 |
| Gask1b | 3.664546903 | 2.22E-10 |
| Mamdc2 | 3.550462167 | 0.000209676 |
| Kbtbd12 | 3.514263513 | 0.021076851 |
| Erc2 | 3.506109588 | 9.93E-15 |
| Krt19 | 3.474224087 | 8.84E-08 |
| Mybphl | 3.381163647 | 0.015272644 |
| Nid2 | 3.331790063 | 2.84E-17 |
| 4933417E11Rik | 3.29329326 | 0.013474331 |
| Epor | 3.219483367 | 0.002685064 |
| Pwwp4c | 3.209273459 | 0.004010952 |
| Tc2n | 3.205820022 | 6.84E-11 |
| Crtac1 | 3.167638783 | 0.027604821 |
| Pnpla5 | 3.093989556 | 1.70E-06 |
| Gm34280 | 3.087640606 | 0.036848803 |
| Matn2 | 3.079307762 | 0.000212369 |
| Pcsk1 | 3.076478561 | 0.024010036 |
| Aloxe3 | 3.052787525 | 1.90E-07 |
| Ahsp | 3.027249831 | 1.94E-07 |
| Vstm2a | 3.000330878 | 7.97E-36 |
| Kcne3 | 2.990053499 | 3.33E-07 |
| Pdgfc | 2.986266694 | 0.004286235 |
| Mrc1 | 2.982585528 | 1.81E-16 |
| St6galnac3 | 2.977120778 | 7.97E-06 |
| Mcoln3 | 2.96725583 | 3.22E-14 |
| Celf5 | 2.95551731 | 0.023483322 |
| Elapor1 | 2.954324411 | 0.001990894 |
| Pf4 | 2.942656695 | 9.06E-21 |
| Car4 | 2.936876504 | 6.34E-46 |
| Tcim | 2.936815951 | 6.81E-05 |
| Mcam | 2.914572943 | 1.86E-22 |
| Tmem144 | 2.899283849 | 6.11E-09 |
| Flvcr2 | 2.899199157 | 7.93E-15 |
| Cpne5 | 2.884529607 | 2.94E-09 |
| Kcnn3 | 2.88198002 | 2.07E-20 |
| Cracd | 2.865902097 | 1.99E-05 |
| Fsbp | 2.856162742 | 0.030755076 |
| Fam171a1 | 2.854386774 | 3.20E-08 |
| Gm7901 | 2.843493848 | 0.000876884 |
| Igkv5-48 | 2.830396521 | 0.03942483 |
| Flrt3 | 2.826980217 | 2.85E-25 |
| Prune2 | 2.819539945 | 4.75E-05 |
| Bok | 2.817259864 | 1.47E-08 |
| Zfp2 | 2.815701099 | 0.021385853 |
| Fabp1 | 2.795652136 | 1.23E-07 |
| Bfsp1 | 2.794411709 | 2.96E-10 |
| Lgi4 | 2.776123567 | 4.65E-06 |
| Cpne8 | 2.762499206 | 6.92E-15 |
| Sobp | 2.753509828 | 0.000228384 |
| Atf7ip2 | 2.750102 | 0.01455426 |
| Serpinb1a | 2.745819324 | 6.19E-28 |
| Slc6a4 | 2.737267052 | 0.000401099 |
| Gmpr | 2.726898686 | 2.46E-24 |
| Gcat | 2.717049307 | 0.000184833 |
| Pros1 | 2.715220723 | 3.25E-12 |
| Dclk3 | 2.712272155 | 4.94E-05 |
| Krt15 | 2.705089586 | 0.022150221 |
| Col14a1 | 2.704380265 | 0.000412255 |
| Chd5 | 2.704034357 | 5.59E-10 |
| Hspa12a | 2.68850381 | 1.98E-11 |
| Gcm2 | 2.687165754 | 0.017715795 |
| Klhl33 | 2.67396003 | 3.28E-05 |
| Cmbl | 2.671870254 | 6.53E-06 |
| Sema3e | 2.655719416 | 1.71E-11 |
| A730085K08Rik | 2.641877058 | 2.77E-09 |
| Cd109 | 2.638495503 | 4.23E-08 |
| Adam22 | 2.629928427 | 1.40E-14 |
| Entrep1 | 2.598518462 | 0.004996478 |
| Thbd | 2.586974517 | 1.80E-20 |
| Gm46339 | 2.580505741 | 0.032064087 |
| Agmo | 2.579490976 | 1.39E-08 |
| Kctd12b | 2.577555368 | 1.29E-10 |
| Marco | 2.573569952 | 7.11E-30 |
| Sdk1 | 2.570788939 | 4.80E-07 |
| Adgrl3 | 2.564909083 | 2.63E-12 |
| Klrg2 | 2.512038149 | 0.000702531 |
| Ocln | 2.507819715 | 6.35E-07 |
| Carmil3 | 2.474137229 | 0.005337083 |
| 2610203C22Rik | 2.470151103 | 2.23E-05 |
| Spef1 | 2.469113496 | 9.43E-12 |
| Tln2 | 2.466752893 | 0.000562786 |
| Lypd6b | 2.457653884 | 0.000151737 |
| Rem1 | 2.436278757 | 0.000413473 |
| Coro2b | 2.430419707 | 2.86E-09 |
| Adamtsl5 | 2.426449198 | 1.41E-09 |
| Perp | 2.420262994 | 9.50E-15 |
| Gm45844 | 2.419745946 | 0.012264629 |
| Amz1 | 2.418130266 | 0.000939607 |
| Frk | 2.411734398 | 2.61E-07 |
| Igf1 | 2.407511346 | 7.11E-05 |
| Kif15 | 2.405114643 | 7.31E-10 |
| Anln | 2.396729229 | 0.004999187 |
| Gm6116 | 2.395984961 | 3.33E-06 |
| Colec12 | 2.385727269 | 1.23E-19 |
| Sapcd2 | 2.383169607 | 5.46E-07 |
| Sh3bgrl2 | 2.382572296 | 1.43E-11 |
| Gm11223 | 2.378011317 | 6.24E-05 |
| Igsf3 | 2.374371495 | 2.52E-09 |
| Gm44950 | 2.371951972 | 0.000163076 |
| Bmal2 | 2.370307654 | 0.003674474 |
| Patj | 2.367508904 | 1.86E-15 |
| Plet1 | 2.365053563 | 0.000295719 |
| Zdhhc2 | 2.361299353 | 1.38E-08 |
| Coro6 | 2.360031644 | 8.49E-08 |
| Gm20479 | 2.359513758 | 0.041485692 |
| Sorbs3 | 2.348016167 | 1.25E-11 |
| Gm26588 | 2.34279883 | 0.003083364 |
| Kcnk5 | 2.330714377 | 0.031885768 |
| Tmem41a | 2.326035415 | 2.18E-07 |
| Gm14221 | 2.31995118 | 1.26E-05 |
| Clmn | 2.319158452 | 3.61E-10 |
| Adgrg1 | 2.314277194 | 3.39E-22 |
| Nxpe5 | 2.307965228 | 1.44E-09 |
| Cox6b2 | 2.301912867 | 9.85E-05 |
| Ctsk | 2.301714269 | 2.60E-12 |
| 8030453O22Rik | 2.301677658 | 1.28E-09 |
| Ccl8 | 2.301554073 | 7.73E-05 |
| Phgdh | 2.29990819 | 9.75E-12 |
| Crppa | 2.295279742 | 0.004031328 |
| Cidec | 2.294994667 | 1.08E-10 |
| Hacd1 | 2.289619606 | 3.81E-07 |
| D7Ertd128e | 2.289328699 | 0.000357117 |
| Clec4b1 | 2.286874818 | 0.000632946 |
| Pdlim1 | 2.271090541 | 0.003174983 |
| B3gnt7 | 2.268388489 | 5.49E-09 |
| Celf4 | 2.267644448 | 0.00207084 |
| Mgl2 | 2.266235433 | 0.0077579 |
| 2810025M15Rik | 2.265921632 | 2.32E-10 |
| Gsg1 | 2.264619027 | 0.004903091 |
| Serpinb6a | 2.256771713 | 5.22E-16 |
| Tacc2 | 2.256080572 | 4.32E-09 |
| A530058O07Rik | 2.249295301 | 0.009056911 |
| Slc39a12 | 2.244934324 | 0.007130911 |
| Scel | 2.240194014 | 2.07E-05 |
| Asb4 | 2.239758117 | 4.15E-10 |
| Mlph | 2.237554228 | 3.24E-10 |
| Il18 | 2.231625777 | 1.49E-12 |
| Ces2c | 2.23013855 | 0.003511432 |
| Stac | 2.229655189 | 3.34E-06 |
| Cenpf | 2.22925924 | 0.039917733 |
| Eid2 | 2.223017721 | 0.001395922 |
| Shcbp1l | 2.213736637 | 0.000660551 |
| Ntn4 | 2.206574684 | 0.00159082 |
| A730089K16Rik | 2.204204798 | 0.01015224 |
| Cx3cl1 | 2.203232052 | 1.24E-05 |
| Ucp3 | 2.201266742 | 8.29E-08 |
| Cyb561 | 2.196007713 | 5.99E-14 |
| Trf | 2.187798742 | 1.75E-17 |
| Slco2b1 | 2.186243244 | 2.91E-21 |
| Lrfn4 | 2.185552168 | 6.92E-06 |
| Mmp19 | 2.183678669 | 5.04E-19 |
| Ltc4s | 2.183497238 | 1.74E-19 |
| Dysf | 2.181273722 | 0.000298215 |
| Fzd4 | 2.180054801 | 1.59E-13 |
| Aspm | 2.178767691 | 0.009111217 |
| Gal | 2.178493246 | 3.24E-06 |
| Bcar3 | 2.177973046 | 1.28E-09 |
| Prox1 | 2.174382939 | 8.24E-08 |
| Maoa | 2.169563129 | 2.94E-14 |
| Cpeb1 | 2.165124247 | 2.77E-05 |
| Pgf | 2.163732545 | 0.002526842 |
| Zfp979 | 2.159098185 | 0.000324303 |
| Arhgef40 | 2.158016457 | 3.53E-14 |
| Fzd8 | 2.157735297 | 5.36E-06 |
| Gm41485 | 2.156577416 | 1.11E-10 |
| Ccdc80 | 2.155825426 | 0.001523696 |
| Gm13597 | 2.147297215 | 0.024969835 |
| Hmmr | 2.14560511 | 0.008874688 |
| 2810408I11Rik | 2.145595835 | 0.010603407 |
| Sh2d1b1 | 2.144483976 | 2.01E-16 |
| Prr15 | 2.142664847 | 8.34E-05 |
| Plxdc2 | 2.138023182 | 1.20E-07 |
| Pmp22 | 2.132694515 | 5.57E-15 |
| Fkbp9 | 2.130627514 | 1.89E-08 |
| Tmem273 | 2.123968167 | 2.12E-11 |
| Gm33251 | 2.12047904 | 0.027614516 |
| Pimreg | 2.120181453 | 0.000681441 |
| Cyp2ab1 | 2.119383011 | 6.75E-06 |
| Ccl6 | 2.113330246 | 2.45E-15 |
| Rbm44 | 2.100792965 | 0.005224356 |
| Lilra5 | 2.097139418 | 6.35E-07 |
| Enpp1 | 2.096328442 | 3.09E-12 |
| Zcchc14 | 2.096022323 | 7.43E-08 |
| Birc5 | 2.095785264 | 0.00873821 |
| Pls3 | 2.095095966 | 2.89E-10 |
| Stau2 | 2.09467431 | 1.93E-08 |
| 1700030C10Rik | 2.093488479 | 0.012763425 |
| Emp1 | 2.090635979 | 3.27E-27 |
| Cdc42bpa | 2.088522539 | 2.38E-19 |
| Phlda3 | 2.07725309 | 2.87E-06 |
| Hebp1 | 2.076995241 | 2.66E-16 |
| Ckap2 | 2.071909344 | 2.77E-08 |
| Ccnb1 | 2.068675616 | 0.013349329 |
| Prickle2 | 2.066530615 | 2.78E-09 |
| Mmp12 | 2.062920636 | 1.74E-07 |
| Lpin1 | 2.060919223 | 5.73E-08 |
| Fam187b | 2.057691867 | 0.007220273 |
| Afap1 | 2.056184698 | 1.14E-09 |
| Ffar4 | 2.050136415 | 3.18E-06 |
| Mkx | 2.049585689 | 0.013794354 |
| Ankrd55 | 2.042791699 | 0.034468617 |
| Adarb1 | 2.03937839 | 2.29E-10 |
| Acsbg3 | 2.030694182 | 0.003155066 |
| Dnah11 | 2.03013488 | 0.008223666 |
| Ttll7 | 2.027895902 | 0.040832106 |
| Gprc5c | 2.026423065 | 1.42E-06 |
| Rbms3 | 2.024167339 | 0.000367511 |
| Tpbgl | 2.020944874 | 1.18E-07 |
| Dip2c | 2.019432073 | 6.17E-15 |
| Plscr4 | 2.014592233 | 3.91E-10 |
| Tarsl2 | 2.009434649 | 1.74E-08 |
| Grb7 | 2.005348028 | 1.54E-08 |
| Syde2 | 2.00461619 | 3.33E-09 |
| Gm47815 | 2.00223404 | 5.83E-08 |
| Macrod2 | 2.002149921 | 0.007759568 |
| Dhcr24 | 2.000327998 | 1.08E-06 |
| Kif2c | 2.000130221 | 0.000120275 |
| Gm49254 | 1.999768738 | 3.18E-07 |
| Nptxr | 1.997807248 | 0.000433719 |
| Gm30605 | 1.993351437 | 2.06E-05 |
| Sbk2 | 1.985209416 | 4.63E-05 |
| Prickle1 | 1.984435289 | 0.01891741 |
| Cdkl2 | 1.98347423 | 3.00E-07 |
| Ccna2 | 1.983360033 | 0.009121717 |
| Myrf | 1.982244372 | 2.01E-06 |
| Pter | 1.977025217 | 1.45E-07 |
| Mical3 | 1.973935198 | 7.61E-11 |
| Lyplal1 | 1.969427992 | 5.79E-06 |
| Xlr | 1.967488196 | 7.45E-07 |
| AU022793 | 1.964078647 | 3.54E-06 |
| Bub1 | 1.961586646 | 1.06E-05 |
| Pparg | 1.961375221 | 8.04E-11 |
| Prr11 | 1.961230314 | 1.89E-05 |
| Slc9a2 | 1.961021778 | 7.04E-09 |
| Wtip | 1.957286099 | 0.002053001 |
| Cep55 | 1.956225848 | 1.57E-05 |
| Tle2 | 1.955548968 | 7.03E-06 |
| Bzw2 | 1.951526064 | 1.44E-13 |
| Trim47 | 1.946171362 | 6.75E-06 |
| Jup | 1.94350945 | 8.17E-10 |
| Tcaf1 | 1.94326158 | 5.63E-06 |
| Mfge8 | 1.942513794 | 6.37E-14 |
| Gm10479 | 1.94109717 | 0.003166571 |
| Plcl1 | 1.940154825 | 0.007160578 |
| Kif20a | 1.938507491 | 0.00846705 |
| Atp6v0d2 | 1.937111539 | 3.64E-15 |
| Cd163 | 1.936201134 | 0.000199482 |
| Fn1 | 1.933953698 | 6.92E-23 |
| Adk | 1.931158024 | 7.05E-13 |
| Fmo1 | 1.930928683 | 0.005700408 |
| Cdkn3 | 1.930122936 | 1.82E-05 |
| Mamld1 | 1.928059593 | 5.94E-13 |
| D930019O06Rik | 1.926264911 | 0.000505815 |
| Adgrl2 | 1.924458507 | 8.63E-21 |
| Mical2 | 1.922170017 | 3.30E-08 |
| Stac2 | 1.921889207 | 4.43E-09 |
| Kazn | 1.921011193 | 6.14E-06 |
| Gm2694 | 1.919934181 | 0.008848684 |
| Pwwp4a | 1.919858584 | 0.045411072 |
| Ncs1 | 1.918053545 | 0.002417315 |
| Cdca3 | 1.917147983 | 4.65E-06 |
| Lrig3 | 1.913192916 | 6.03E-05 |
| Gm32695 | 1.913123748 | 0.002260956 |
| Lepr | 1.912205643 | 1.80E-06 |
| Depdc1a | 1.9120184 | 0.000197638 |
| Fam89a | 1.908535692 | 3.76E-08 |
| Ube2c | 1.908162162 | 0.020019136 |
| Gm47507 | 1.907661637 | 4.94E-05 |
| Fbl-ps2 | 1.906092801 | 0.000908131 |
| Cd200r1 | 1.901043683 | 8.37E-11 |
| Fcgrt | 1.898871735 | 4.86E-10 |
| Mrgpre | 1.892852974 | 4.20E-10 |
| Ankrd13b | 1.891949164 | 1.06E-11 |
| Reps2 | 1.890939389 | 2.41E-14 |
| Ehbp1 | 1.889173064 | 3.37E-09 |
| ENSMUSG00000121727 | 1.88415963 | 0.006987459 |
| Kcnip4 | 1.883510579 | 0.029867987 |
| Zfp976 | 1.87972335 | 0.000333185 |
| Oip5 | 1.878194709 | 2.65E-05 |
| Fabp4 | 1.878188117 | 2.12E-13 |
| Prkn | 1.877892893 | 0.001911306 |
| Shcbp1 | 1.877438704 | 5.29E-05 |
| Tpx2 | 1.875931098 | 7.69E-06 |
| Adcy3 | 1.873807227 | 2.03E-09 |
| Slc9a4 | 1.866257297 | 8.05E-07 |
| Epm2a | 1.866073803 | 4.68E-08 |
| Siglecf | 1.864891142 | 9.32E-12 |
| Rasal2 | 1.864031396 | 9.90E-12 |
| Cd2 | 1.862700138 | 1.44E-06 |
| Ehhadh | 1.86183423 | 7.75E-06 |
| Bdh1 | 1.860525434 | 0.000396221 |
| Tfec | 1.858137037 | 1.83E-13 |
| Marveld1 | 1.857339431 | 7.12E-08 |
| Nuf2 | 1.857144797 | 2.24E-05 |
| Map7d3 | 1.853010222 | 5.28E-06 |
| Cenpv | 1.851635342 | 2.30E-07 |
| Ccnb2 | 1.850178658 | 1.61E-06 |
| Nusap1 | 1.849065491 | 9.24E-06 |
| Kif14 | 1.844487782 | 8.38E-05 |
| Camk1 | 1.843452629 | 1.74E-12 |
| Spire1 | 1.843263889 | 3.41E-10 |
| Gpr137b | 1.841096944 | 1.37E-13 |
| Esco2 | 1.840615923 | 3.24E-06 |
| Cenpi | 1.839289772 | 2.31E-05 |
| Pcdhgc3 | 1.837651655 | 2.34E-07 |
| Mxd3 | 1.835543338 | 0.002996361 |
| Net1 | 1.830127873 | 3.73E-09 |
| Cdca2 | 1.829241899 | 2.12E-05 |
| Plk1 | 1.829167977 | 9.13E-05 |
| Trim29 | 1.828584306 | 4.93E-10 |
| Jcad | 1.825699765 | 1.06E-11 |
| Ttyh2 | 1.823957774 | 6.53E-10 |
| Kif11 | 1.820231439 | 2.74E-06 |
| Ttc39aos1 | 1.816272273 | 0.001364802 |
| Lima1 | 1.814152813 | 5.04E-12 |
| Parpbp | 1.813021391 | 0.001104719 |
| Mybl1 | 1.809924173 | 5.72E-05 |
| Mtss1 | 1.807823803 | 2.04E-07 |
| Acy1 | 1.806996953 | 1.27E-08 |
| Uaca | 1.806656178 | 7.03E-06 |
| Ltbp4 | 1.806130275 | 0.027802115 |
| Smim5 | 1.805503201 | 0.000419784 |
| Gm26542 | 1.802006156 | 0.003845173 |
| Fkbp11 | 1.800543772 | 0.000656307 |
| Depdc1b | 1.800530119 | 9.01E-05 |
| Areg | 1.80052139 | 0.000137949 |
| Sec14l2 | 1.799931383 | 0.000135044 |
| Tle6 | 1.795047183 | 0.01385303 |
| Nr1d2 | 1.792888867 | 1.70E-11 |
| Lca5 | 1.792132364 | 4.88E-05 |
| Cdc20 | 1.791146298 | 0.000178555 |
| Epas1 | 1.790493894 | 1.83E-20 |
| Trim46 | 1.788216589 | 1.83E-05 |
| Prc1 | 1.786129962 | 1.41E-05 |
| Ak8 | 1.784140995 | 1.88E-05 |
| Aldoc | 1.783878919 | 1.23E-05 |
| Pbk | 1.783283289 | 2.93E-07 |
| Rad54b | 1.782471847 | 3.68E-08 |
| Spc24 | 1.782322712 | 0.00041969 |
| Diaph3 | 1.781864943 | 0.000258087 |
| Mccc2 | 1.781009047 | 8.96E-14 |
| Ccbe1 | 1.772769576 | 2.33E-07 |
| Spry1 | 1.772761736 | 3.63E-06 |
| Rufy4 | 1.769920943 | 2.12E-18 |
| Tk1 | 1.76746174 | 1.11E-06 |
| Sgo2a | 1.766794905 | 4.47E-05 |
| Rac3 | 1.76594209 | 3.47E-06 |
| Cenpe | 1.762440862 | 0.020699534 |
| Bcas1 | 1.760416908 | 0.017959114 |
| Sort1 | 1.757114481 | 5.05E-15 |
| Akap5 | 1.757069412 | 2.85E-05 |
| Stmn1 | 1.755021426 | 5.87E-07 |
| Tcf24 | 1.751963283 | 0.007427996 |
| Gm16096 | 1.750252329 | 6.00E-06 |
| Gm9530 | 1.749736182 | 0.002842969 |
| Podn | 1.747237411 | 0.047378406 |
| Tppp3 | 1.744737454 | 5.17E-05 |
| Kif7 | 1.744398988 | 0.00071452 |
| Kazald1 | 1.743895168 | 0.000257278 |
| Ncapg | 1.742565852 | 7.46E-05 |
| Knstrn | 1.741182806 | 8.46E-06 |
| Slc22a17 | 1.740601961 | 1.14E-06 |
| Ankle1 | 1.740452362 | 0.004741374 |
| Tchh | 1.74032895 | 0.000104781 |
| Angptl4 | 1.736299028 | 4.88E-21 |
| Pif1 | 1.734492325 | 0.00089293 |
| Vash1 | 1.730457855 | 5.17E-06 |
| Cd81 | 1.729151895 | 8.99E-12 |
| Syp | 1.727722145 | 0.012869874 |
| Plxna2 | 1.727459569 | 0.000154131 |
| Deptor | 1.727365178 | 3.63E-14 |
| Dab2 | 1.724260484 | 1.26E-29 |
| Sulf2 | 1.721292113 | 0.011029832 |
| C130074G19Rik | 1.719533795 | 0.017519665 |
| Syn1 | 1.718654416 | 7.58E-10 |
| Lipf | 1.715360338 | 1.93E-09 |
| Mastl | 1.712133355 | 8.46E-07 |
| Ccdc18 | 1.71156039 | 0.001180194 |
| Ttk | 1.710974033 | 2.87E-06 |
| Cd9 | 1.707363374 | 8.52E-20 |
| Mgll | 1.701303563 | 6.12E-11 |
| Cenpn | 1.699888816 | 0.000201975 |
| 5730507C01Rik | 1.696344793 | 3.61E-05 |
| Acaa1b | 1.696200687 | 2.74E-05 |
| Zbtb8a | 1.696072208 | 1.10E-05 |
| Kif18b | 1.693202807 | 0.000333365 |
| Kntc1 | 1.69182653 | 1.94E-05 |
| Gm11496 | 1.689081482 | 0.000313706 |
| Neurl1b | 1.688658076 | 0.000226709 |
| Pomgnt2 | 1.688458533 | 0.007249619 |
| Bend6 | 1.686193626 | 6.20E-06 |
| Olfm1 | 1.684172168 | 1.78E-10 |
| C77080 | 1.683337486 | 1.96E-06 |
| Rnf180 | 1.682607672 | 2.88E-11 |
| Gm44956 | 1.677979846 | 0.049776324 |
| Spp1 | 1.67790978 | 1.94E-17 |
| Dnajb13 | 1.6778614 | 1.96E-06 |
| Echdc1 | 1.673023977 | 5.43E-12 |
| Tek | 1.67293183 | 0.022552564 |
| Dsel | 1.672381726 | 4.19E-06 |
| Sqle | 1.670727843 | 7.89E-08 |
| Ccdc168 | 1.669792203 | 0.009121717 |
| Etv1 | 1.669606996 | 0.001233293 |
| Spc25 | 1.66868877 | 0.00084753 |
| Wwtr1 | 1.665924255 | 2.65E-08 |
| Arhgef39 | 1.66514737 | 6.70E-05 |
| Ska2 | 1.663427756 | 0.000406936 |
| Rgl3 | 1.66246578 | 2.05E-06 |
| Large1 | 1.655604925 | 6.83E-16 |
| Map6 | 1.655405406 | 9.55E-10 |
| Racgap1 | 1.652637589 | 1.94E-06 |
| Nt5e | 1.651939106 | 6.94E-17 |
| Akr1b8 | 1.645970003 | 2.70E-06 |
| Lims2 | 1.645076335 | 0.028901604 |
| Ctsf | 1.640544265 | 0.001660705 |
| 2210418O10Rik | 1.638695381 | 0.004206201 |
| Slc52a3 | 1.632463857 | 1.08E-10 |
| Kitl | 1.628145465 | 0.03833194 |
| Foxm1 | 1.626435055 | 2.73E-05 |
| Cip2a | 1.624841438 | 1.05E-05 |
| Cdca8 | 1.622191127 | 6.92E-06 |
| Cd22 | 1.622124022 | 2.48E-06 |
| Tmem64 | 1.622034862 | 3.09E-08 |
| Cdc25c | 1.618441482 | 0.000342812 |
| Ska1 | 1.618149195 | 0.001577566 |
| Cerkl | 1.616565247 | 0.002633622 |
| Marchf3 | 1.615964465 | 2.77E-05 |
| Ttc39a | 1.612906516 | 9.30E-09 |
| Nr3c2 | 1.612834692 | 0.010424584 |
| Osbp2 | 1.612601181 | 0.001910935 |
| Lmna | 1.611516095 | 6.62E-09 |
| Cnksr3 | 1.611449942 | 1.37E-08 |
| Septin8 | 1.610189634 | 5.01E-10 |
| Abca5 | 1.607638656 | 0.00369964 |
| Fancd2 | 1.607275636 | 1.59E-06 |
| Scn7a | 1.605163498 | 0.044474844 |
| Cldn34c1 | 1.602745338 | 0.007672227 |
| Gm4924 | 1.600886926 | 0.012650542 |
| Ndc80 | 1.600633723 | 8.09E-05 |
| Myo7a | 1.598608821 | 1.59E-08 |
| Cxcl14 | 1.597181321 | 0.000471122 |
| Kbtbd6 | 1.595323994 | 0.042321374 |
| Zfp704 | 1.595072669 | 1.58E-06 |
| Tmsb15l | 1.592777035 | 0.040905022 |
| Knl1 | 1.591535952 | 0.001244185 |
| Troap | 1.589076412 | 0.001882451 |
| Zfp1008 | 1.586466579 | 4.51E-06 |
| Adamts2 | 1.585483875 | 1.99E-09 |
| Sult4a1 | 1.581773992 | 0.02399889 |
| Nek2 | 1.580479326 | 0.000315579 |
| Kif23 | 1.579842195 | 1.32E-10 |
| Vwf | 1.578882796 | 1.35E-18 |
| Maged1 | 1.575820628 | 0.000333814 |
| Epcam | 1.575047731 | 0.00954383 |
| Ahnak2 | 1.575000807 | 5.20E-16 |
| Dbndd2 | 1.574308688 | 4.33E-06 |
| Cenph | 1.573997456 | 0.000511666 |
| Fignl1 | 1.570034719 | 4.45E-06 |
| Ednrb | 1.568731704 | 0.000599617 |
| Nynrin | 1.568387038 | 0.045876234 |
| Dusp9 | 1.567925117 | 0.002871515 |
| Rfx2 | 1.567731469 | 1.79E-06 |
| Pald1 | 1.56522314 | 8.53E-06 |
| Lrp12 | 1.564929258 | 6.64E-14 |
| Bpifb1 | 1.563959801 | 0.008569994 |
| Cdk1 | 1.563897079 | 1.30E-05 |
| Kif4 | 1.562269957 | 0.002909145 |
| Mt1 | 1.560919392 | 6.08E-18 |
| Cdon | 1.559363634 | 3.26E-07 |
| S100a1 | 1.557677231 | 7.42E-11 |
| ENSMUSG00000121730 | 1.557250566 | 0.001123659 |
| Slc12a7 | 1.555448782 | 5.23E-07 |
| Nfia | 1.553499134 | 2.86E-11 |
| Nherf2 | 1.550441384 | 6.34E-06 |
| Acy3 | 1.546234037 | 0.015475946 |
| Dlgap5 | 1.545349756 | 1.31E-05 |
| Poglut3 | 1.541672252 | 0.000296289 |
| Cubn | 1.540602593 | 0.006043611 |
| Crip1 | 1.53963501 | 3.50E-05 |
| Cideb | 1.538349641 | 1.37E-06 |
| Krt79 | 1.533425737 | 3.12E-08 |
| Zfp960 | 1.528898116 | 0.008715756 |
| Aurkb | 1.527736134 | 0.00011547 |
| Ppfibp1 | 1.525844223 | 5.96E-08 |
| Gm44250 | 1.525535006 | 2.48E-09 |
| Adam33 | 1.521608508 | 0.000445074 |
| Zfp532 | 1.52099044 | 0.00012558 |
| Neil3 | 1.520824699 | 0.000737903 |
| Arhgap6 | 1.520341594 | 0.000306149 |
| Asf1b | 1.519735622 | 3.92E-05 |
| Ncapd2 | 1.51900278 | 1.01E-05 |
| Bckdhb | 1.516110714 | 3.59E-13 |
| Nipa1 | 1.515132347 | 4.14E-05 |
| Tbc1d16 | 1.513055083 | 2.45E-08 |
| Slc18b1 | 1.508841846 | 0.003601669 |
| Psrc1 | 1.508369955 | 3.82E-07 |
| Cyp2f2 | 1.50406171 | 0.000216043 |
| Rad51ap1 | 1.503918067 | 0.002367601 |
| Celsr2 | 1.502208674 | 0.023159867 |
| Gas6 | 1.500211548 | 2.38E-05 |
| Ccne2 | 1.499879848 | 1.51E-05 |
| Dpt | 1.499830022 | 0.040337141 |
| Ucp1 | 1.49972436 | 0.002718446 |
| Sbf2 | 1.498642986 | 6.84E-10 |
| Marveld2 | 1.498020592 | 9.07E-06 |
| Melk | 1.497928872 | 0.002101447 |
| Synpo | 1.496216323 | 0.000358177 |
| Shroom3 | 1.495396553 | 0.003402743 |
| Cercam | 1.493414223 | 7.69E-06 |
| Pask | 1.4932799 | 3.00E-05 |
| Ect2 | 1.491485104 | 8.14E-05 |
| Abcd2 | 1.490627832 | 3.22E-27 |
| Bpifa1 | 1.490283884 | 0.016306627 |
| Zfp770 | 1.489946177 | 2.14E-06 |
| Adgra3 | 1.486994959 | 0.001517758 |
| Hlf | 1.486217835 | 0.019814711 |
| Tpk1 | 1.485334069 | 1.99E-05 |
| C2cd2l | 1.48353043 | 1.48E-05 |
| Xylb | 1.478649908 | 4.60E-07 |
| Mrps6 | 1.477864016 | 3.14E-05 |
| Penk | 1.476674017 | 0.013455215 |
| Acox1 | 1.476661862 | 4.05E-15 |
| E2f7 | 1.475804846 | 0.000252733 |
| Acvr2b | 1.472812798 | 0.000451386 |
| Slc29a1 | 1.469869884 | 8.82E-07 |
| Pir | 1.469623868 | 0.000356549 |
| Bhlhe41 | 1.465621076 | 1.24E-07 |
| Gpsm2 | 1.464508784 | 0.003405524 |
| ENSMUSG00000121820 | 1.464051906 | 0.018156407 |
| Dagla | 1.463821556 | 2.10E-06 |
| Iqgap3 | 1.463171534 | 0.000139263 |
| Kif20b | 1.46302822 | 2.00E-06 |
| Lzts2 | 1.460938909 | 0.000956609 |
| Mis18bp1 | 1.460862604 | 6.28E-05 |
| Kif22 | 1.459761607 | 5.71E-05 |
| Gm9920 | 1.457014528 | 0.030096118 |
| Myo6 | 1.456677784 | 1.31E-05 |
| Pcx | 1.456360857 | 1.16E-06 |
| Gen1 | 1.456146423 | 0.000466567 |
| Galnt3 | 1.455882409 | 2.13E-10 |
| Cep170b | 1.454828959 | 4.40E-08 |
| Rab3il1 | 1.454511815 | 1.91E-09 |
| Lyz2 | 1.45301063 | 2.36E-05 |
| Kif3a | 1.452853385 | 1.41E-17 |
| Marchf4 | 1.452648985 | 0.009917397 |
| Cstpp1 | 1.45213127 | 0.000100036 |
| Ank2 | 1.451172467 | 0.037870421 |
| Tmem238 | 1.450488976 | 0.010662409 |
| Lrr1 | 1.450386553 | 0.04282411 |
| Rhoc | 1.449471953 | 4.31E-08 |
| Tnfsf13 | 1.447275019 | 2.21E-06 |
| Septin11 | 1.442692109 | 4.45E-08 |
| Ppp1r9a | 1.442579688 | 0.000125103 |
| Fam181b | 1.442336805 | 0.021486169 |
| Fanci | 1.440866765 | 0.001848004 |
| Gtse1 | 1.438598273 | 0.001374807 |
| Trim16 | 1.438310548 | 1.55E-05 |
| Gstm5 | 1.435091761 | 0.011706313 |
| Dtna | 1.434300657 | 0.003064105 |
| Pcdh1 | 1.430549191 | 0.041230258 |
| Pltp | 1.429226874 | 2.12E-13 |
| Stil | 1.429156098 | 0.0049657 |
| Spag5 | 1.428610776 | 0.00039799 |
| Gpr176 | 1.427581706 | 0.005637827 |
| Arhgef12 | 1.425105024 | 6.60E-06 |
| Kifc1 | 1.424836795 | 2.59E-05 |
| Fgf1 | 1.42380011 | 0.039768494 |
| Cit | 1.423774196 | 0.001463923 |
| Tmcc3 | 1.423567874 | 0.000500005 |
| Cdh1 | 1.421772194 | 3.88E-07 |
| Pcyox1 | 1.420612495 | 4.44E-15 |
| Bub1b | 1.419062135 | 1.21E-06 |
| Apcdd1 | 1.419058792 | 0.002732042 |
| Htr2c | 1.418977172 | 5.43E-05 |
| Kif21a | 1.41850881 | 0.005506337 |
| Mgst3 | 1.417845456 | 0.000118648 |
| Top2a | 1.417057812 | 0.001242407 |
| Ccdc34 | 1.416523876 | 9.84E-06 |
| Msrb2 | 1.416510155 | 0.000356778 |
| 2610318N02Rik | 1.415896777 | 0.034996701 |
| Prom1 | 1.415168842 | 4.57E-14 |
| Bc1 | 1.414641005 | 0.004437278 |
| Adrb1 | 1.412839774 | 0.010964813 |
| Tacc3 | 1.410707357 | 2.60E-06 |
| Dapk1 | 1.41004084 | 1.08E-06 |
| Tyms | 1.408986746 | 0.001911306 |
| Cks1b | 1.408558569 | 5.96E-05 |
| Stap2 | 1.407995555 | 0.004984832 |
| Fzd1 | 1.406169436 | 4.00E-06 |
| Ticrr | 1.405562632 | 0.000640055 |
| Cenpu | 1.404175302 | 0.004933982 |
| Fsd1l | 1.399071626 | 0.016672203 |
| Cdc25b | 1.399051742 | 1.16E-06 |
| Caprin2 | 1.398892156 | 0.006998794 |
| Apbb2 | 1.398377394 | 0.021138854 |
| Tnfaip8l1 | 1.396382801 | 0.002093264 |
| Paqr4 | 1.394878684 | 0.000136431 |
| Uck2 | 1.393410227 | 7.43E-16 |
| Suclg2 | 1.39299757 | 7.67E-08 |
| Unc13b | 1.392948728 | 0.024564 |
| Lsr | 1.390374279 | 5.01E-05 |
| Spaca9 | 1.39024817 | 0.042364772 |
| Sox4 | 1.389977446 | 1.03E-09 |
| Acot4 | 1.388419725 | 0.015655202 |
| Ramp1 | 1.387748338 | 1.29E-07 |
| Comt | 1.384227507 | 5.81E-09 |
| Il1rl1 | 1.384068457 | 0.006767108 |
| Trim37 | 1.381324841 | 8.18E-07 |
| Lgals4 | 1.379314782 | 0.002066631 |
| Ccdc88a | 1.377703829 | 2.52E-10 |
| Aplp2 | 1.376300125 | 4.38E-13 |
| Igf2bp3 | 1.375121497 | 0.021830078 |
| Aurka | 1.373069421 | 1.45E-06 |
| Arpin | 1.371910359 | 0.00228214 |
| Enkur | 1.37144516 | 0.006120884 |
| 2210408I21Rik | 1.371164535 | 0.03744518 |
| Igf2bp2 | 1.368842776 | 0.000229002 |
| Mak | 1.368754507 | 6.05E-06 |
| Sord | 1.368352288 | 7.42E-10 |
| Nid1 | 1.365179972 | 0.007797842 |
| Ncaph | 1.3628149 | 7.56E-05 |
| Sspn | 1.362266179 | 0.012778349 |
| Raet1d | 1.360868107 | 0.00092102 |
| Efcab11 | 1.360867848 | 0.011657941 |
| Zfp970 | 1.36012416 | 0.006805563 |
| Kif24 | 1.359165091 | 0.00845557 |
| Erbb2 | 1.357813402 | 0.017533085 |
| Ska3 | 1.35777631 | 0.00271585 |
| Rrm2 | 1.357761262 | 0.000232058 |
| Irgc1 | 1.357224234 | 0.004859124 |
| Laptm4b | 1.356476059 | 2.48E-09 |
| Gins2 | 1.35619669 | 8.69E-05 |
| Cav1 | 1.356190363 | 2.74E-08 |
| Emp2 | 1.355175878 | 0.011940762 |
| Cdc14b | 1.354914026 | 1.60E-08 |
| F7 | 1.35486937 | 7.61E-11 |
| Fbxl2 | 1.351560789 | 0.000224449 |
| Chaf1a | 1.351371806 | 1.90E-06 |
| Anxa1 | 1.350901231 | 2.94E-14 |
| Car5b | 1.350314781 | 1.57E-06 |
| Rad54l | 1.348857401 | 0.000438862 |
| Cd200r4 | 1.348716239 | 0.00014703 |
| Aldh1l1 | 1.344977602 | 0.001461409 |
| Uhrf1 | 1.344809997 | 1.08E-05 |
| Itgax | 1.343201461 | 4.87E-05 |
| Mtss2 | 1.342027753 | 0.00515693 |
| Pla2g15 | 1.336586367 | 4.43E-09 |
| Tns1 | 1.335936826 | 4.47E-10 |
| Pcdhgc4 | 1.334047315 | 0.040657781 |
| Akap1 | 1.333741949 | 8.18E-06 |
| Meis3 | 1.333527941 | 8.07E-05 |
| Ift43 | 1.327788854 | 0.000284592 |
| Kif18a | 1.325094073 | 0.000632633 |
| Ly75 | 1.324094151 | 1.69E-06 |
| Acss1 | 1.318749388 | 3.24E-06 |
| Per3 | 1.314356543 | 8.64E-05 |
| Fancl | 1.310744574 | 1.88E-05 |
| Vat1 | 1.310372348 | 1.59E-08 |
| P2rx5 | 1.309394948 | 0.02459953 |
| Tanc2 | 1.308890869 | 0.007623457 |
| Idh1 | 1.307855697 | 5.99E-10 |
| G730013B05Rik | 1.307288789 | 0.017005931 |
| Cdca5 | 1.306920944 | 0.001422899 |
| Mt2 | 1.306335473 | 5.22E-07 |
| Tcf7l2 | 1.306176848 | 3.60E-11 |
| Cc2d2a | 1.302418231 | 0.004056107 |
| Vegfb | 1.300290649 | 1.15E-05 |
| Gmds | 1.299190826 | 0.000140042 |
| Fsd2 | 1.298672664 | 0.038981844 |
| Mfap3l | 1.298201871 | 1.54E-05 |
| Mcoln2 | 1.297185492 | 0.00446004 |
| Pdk4 | 1.292629108 | 0.00181145 |
| Cav2 | 1.291401461 | 1.09E-05 |
| Cdc42bpb | 1.29042472 | 2.31E-08 |
| Dcun1d4 | 1.290191532 | 0.000207371 |
| Mob3b | 1.286570972 | 7.36E-12 |
| Prdm16 | 1.285715951 | 0.016298603 |
| Cryzl2 | 1.283139883 | 3.63E-06 |
| Scamp5 | 1.282829466 | 0.020349129 |
| Pnpla8 | 1.281480507 | 9.85E-18 |
| Pld3 | 1.279797686 | 1.41E-06 |
| Ttc21b | 1.278246513 | 0.000101957 |
| Oxct1 | 1.276576591 | 2.21E-06 |
| Slc35g1 | 1.276458289 | 4.00E-06 |
| Rasgef1a | 1.273023071 | 0.011100726 |
| Cracr2b | 1.272720024 | 3.11E-05 |
| Soga1 | 1.271877806 | 0.000742414 |
| Dennd2a | 1.270828398 | 2.04E-07 |
| Zfp973 | 1.269586406 | 0.0451345 |
| Rdm1 | 1.268260915 | 0.000227667 |
| Cyp51 | 1.267538658 | 0.000908963 |
| Mad2l1 | 1.264845021 | 0.000465138 |
| Rrm1 | 1.264548303 | 2.44E-05 |
| Pola1 | 1.264518147 | 0.000367004 |
| Ehd2 | 1.262974776 | 1.27E-05 |
| Plekha8 | 1.262497847 | 3.96E-05 |
| Ankrd63 | 1.25830941 | 0.002053629 |
| Dock1 | 1.257560198 | 1.87E-06 |
| Opn3 | 1.257443051 | 0.0380061 |
| Rpusd3 | 1.25649601 | 0.003539407 |
| Gstk1 | 1.251125305 | 0.000629144 |
| Mettl15 | 1.249270411 | 0.017997404 |
| Sgcb | 1.247598656 | 1.50E-05 |
| Prxl2a | 1.24603571 | 5.88E-05 |
| H1f2 | 1.245107375 | 2.06E-06 |
| Ccsap | 1.244945894 | 0.011845248 |
| Ablim2 | 1.24384034 | 0.00845557 |
| Gpr55 | 1.243481706 | 1.99E-08 |
| Hmgb3 | 1.243117038 | 0.010126718 |
| Pkd2 | 1.243086745 | 7.09E-05 |
| Cenpa | 1.239725465 | 5.79E-05 |
| Ccl9 | 1.239082165 | 1.23E-11 |
| Ffar2 | 1.237382653 | 1.15E-11 |
| Pon3 | 1.233702338 | 6.39E-11 |
| Gpr137b-ps | 1.231982136 | 2.02E-06 |
| Rph3a | 1.231206891 | 0.008888075 |
| Phyhd1 | 1.230647199 | 1.56E-05 |
| Slc22a18 | 1.22984199 | 0.004866895 |
| Xrcc5 | 1.228451978 | 4.71E-09 |
| Fah | 1.227736888 | 0.012763425 |
| Steap3 | 1.226743012 | 2.63E-06 |
| Rilp | 1.224306252 | 0.000755462 |
| Clec1b | 1.223559829 | 0.039323273 |
| Atp10a | 1.222604933 | 4.82E-09 |
| Lrp4 | 1.222426882 | 6.72E-08 |
| Prkar2b | 1.221582624 | 7.33E-12 |
| Nln | 1.217962071 | 1.58E-10 |
| Tdrkh | 1.217878526 | 0.005938818 |
| 4930503L19Rik | 1.217768085 | 0.000250275 |
| Kifc3 | 1.217107452 | 6.52E-06 |
| Akr1b1 | 1.216641284 | 2.45E-07 |
| Hip1 | 1.212408185 | 2.66E-08 |
| Cyp2r1 | 1.211044433 | 0.017684134 |
| Mgmt | 1.210159797 | 0.007160197 |
| Gm14305 | 1.21010246 | 0.000901074 |
| Rgmb | 1.209629142 | 0.000226709 |
| Itga1 | 1.209416848 | 0.000628626 |
| Nol4l | 1.209245754 | 3.32E-05 |
| Kcnh4 | 1.208194671 | 0.041504076 |
| Mapre3 | 1.207367173 | 9.45E-07 |
| 2700099C18Rik | 1.206987728 | 0.006028352 |
| Hmgn5 | 1.206867748 | 9.66E-07 |
| Qdpr | 1.201406107 | 2.33E-05 |
| Zfp125 | 1.199855571 | 0.004361783 |
| Abhd14b | 1.199729709 | 6.85E-05 |
| Kpna2 | 1.198946973 | 8.81E-08 |
| Cenpm | 1.198751498 | 0.01494882 |
| Polq | 1.197691091 | 0.005816647 |
| Niban1 | 1.194851152 | 2.83E-07 |
| Garin4 | 1.193236771 | 0.010701484 |
| Arhgap26 | 1.192444327 | 4.80E-08 |
| Rpl21-ps1 | 1.19072571 | 0.045898162 |
| Atxn1 | 1.18966852 | 2.17E-08 |
| Asph | 1.189034837 | 7.05E-07 |
| Clspn | 1.188025274 | 0.00369964 |
| Pdia5 | 1.187566469 | 0.004031328 |
| Fdps | 1.186284244 | 0.00023202 |
| Neo1 | 1.1847756 | 0.000730992 |
| Nqo2 | 1.183446657 | 9.88E-08 |
| Idi1 | 1.180923138 | 8.35E-06 |
| Mtmr10 | 1.180687664 | 1.94E-06 |
| Chtf18 | 1.178754632 | 0.029255266 |
| Hpgd | 1.178674198 | 0.004332774 |
| Tcf19 | 1.176339613 | 0.004518744 |
| Sh3bgrl | 1.171471322 | 5.50E-09 |
| Alox5 | 1.171249818 | 2.07E-08 |
| Gm49331 | 1.170972832 | 0.029804629 |
| Gm4316 | 1.170847148 | 4.45E-06 |
| Zranb3 | 1.17074896 | 0.012827647 |
| Tmem138 | 1.170744828 | 1.92E-06 |
| Nxt2 | 1.167803836 | 5.43E-11 |
| Plcb1 | 1.166053206 | 7.47E-07 |
| 2410004P03Rik | 1.165594373 | 0.039261484 |
| Ttl | 1.164196422 | 2.15E-06 |
| Tmem245 | 1.163993073 | 4.63E-08 |
| Tmem120b | 1.163647852 | 5.22E-05 |
| Cmtm4 | 1.1627442 | 0.007346065 |
| Dipk1a | 1.1611459 | 9.20E-07 |
| Serpine1 | 1.159471679 | 1.46E-06 |
| Lix1l | 1.158443323 | 3.00E-05 |
| Ptpdc1 | 1.157877485 | 0.000391506 |
| Il1r1 | 1.157807036 | 0.001970393 |
| Bard1 | 1.15749862 | 0.008577361 |
| Gm26902 | 1.156382227 | 0.026210431 |
| Ulbp1 | 1.154440664 | 0.002244172 |
| Nceh1 | 1.15440119 | 2.83E-13 |
| Vav2 | 1.151636697 | 1.45E-05 |
| Uxs1 | 1.151632529 | 6.94E-08 |
| Lpl | 1.151479057 | 6.28E-08 |
| Pecr | 1.150513215 | 0.000433379 |
| Coprs | 1.150392599 | 0.009229169 |
| Kat6b | 1.149768597 | 5.86E-08 |
| Zwilch | 1.149479718 | 0.000137819 |
| Pkmyt1 | 1.149382415 | 0.009909059 |
| Fignl2 | 1.148249694 | 0.000983604 |
| Tbxas1 | 1.147068732 | 1.34E-06 |
| Tube1 | 1.144952663 | 0.040667185 |
| E230016K23Rik | 1.144177217 | 0.011397809 |
| Dut | 1.143990238 | 0.006102117 |
| Cat | 1.143090942 | 8.26E-07 |
| Recql4 | 1.1401482 | 0.000194837 |
| Arhgap39 | 1.139813621 | 2.70E-06 |
| Zfp667 | 1.139377721 | 0.006065216 |
| Prdx4 | 1.139002002 | 0.000389619 |
| Creb3l2 | 1.138334573 | 1.17E-06 |
| Mthfd1 | 1.138227675 | 1.05E-06 |
| Firre | 1.137527192 | 5.18E-06 |
| Bmx | 1.13628512 | 1.13E-05 |
| Diras2 | 1.135670331 | 0.000686717 |
| Mcm10 | 1.134417547 | 0.002438747 |
| Mrnip | 1.134049383 | 0.014367037 |
| Ung | 1.133645286 | 0.009418225 |
| Qsox2 | 1.132629791 | 6.77E-07 |
| Lif | 1.131485458 | 3.71E-06 |
| Tmem141 | 1.131068172 | 0.002132891 |
| Cdkn2c | 1.127676534 | 0.002842969 |
| Wee1 | 1.12686266 | 0.000308489 |
| Gm527 | 1.12646567 | 0.02242147 |
| Clstn1 | 1.125839075 | 0.009914725 |
| Rusc2 | 1.123752043 | 4.63E-07 |
| B3glct | 1.123282257 | 7.50E-07 |
| Capn2 | 1.123119403 | 2.13E-08 |
| Hsd17b7 | 1.12192821 | 0.003644399 |
| Arhgap33 | 1.12169594 | 0.032730441 |
| Tmem97 | 1.120719796 | 9.76E-07 |
| Smc2 | 1.120141551 | 0.000115354 |
| Prelid2 | 1.11986506 | 0.01291705 |
| Nrp1 | 1.118930232 | 0.000505721 |
| Cryl1 | 1.117302846 | 5.87E-07 |
| Cdk14 | 1.11614357 | 1.96E-07 |
| Dpagt1 | 1.115949025 | 3.46E-05 |
| Naaa | 1.115912021 | 3.04E-07 |
| Alg8 | 1.115583604 | 0.002718446 |
| Tle1 | 1.113380823 | 0.000154589 |
| Gkap1 | 1.113084967 | 9.00E-05 |
| Snx7 | 1.112754129 | 6.26E-06 |
| Nicn1 | 1.111755382 | 0.000888401 |
| Ptgr3 | 1.110997604 | 5.76E-06 |
| Eml6 | 1.109507465 | 0.000237986 |
| Tmem150a | 1.107568248 | 0.001855314 |
| Nr1d1 | 1.10699081 | 1.72E-05 |
| Hrob | 1.106418109 | 0.009949645 |
| Ube2t | 1.105918749 | 0.025345594 |
| Pon2 | 1.105020985 | 1.95E-10 |
| Rad51 | 1.104943426 | 0.000340645 |
| Fv1 | 1.10396982 | 0.028638044 |
| Lss | 1.103829607 | 3.56E-07 |
| Rab29 | 1.103573356 | 4.26E-05 |
| Cyb561a3 | 1.102926537 | 1.72E-11 |
| Nudt12 | 1.101815684 | 0.031056009 |
| Tusc3 | 1.101429963 | 0.00013157 |
| Gm17230 | 1.100942074 | 0.005431993 |
| Tmem151a | 1.10004233 | 0.016869727 |
| Dusp3 | 1.099797128 | 3.03E-09 |
| Socs2 | 1.099654228 | 8.06E-06 |
| Adipor2 | 1.099170052 | 4.76E-12 |
| Pxdc1 | 1.098176509 | 0.016204672 |
| Ddx11 | 1.09813322 | 0.005047805 |
| Ctnnd1 | 1.097241813 | 6.75E-05 |
| Cacna1b | 1.096839988 | 0.020508567 |
| Dusp18 | 1.095794269 | 0.000462126 |
| Calcrl | 1.095624083 | 0.000139582 |
| Gm5150 | 1.095356779 | 7.74E-08 |
| Prmt2 | 1.094581228 | 8.56E-05 |
| Dhrs7b | 1.092609508 | 7.96E-08 |
| 4933404O12Rik | 1.092415447 | 0.0368342 |
| Slc9a6 | 1.091503105 | 1.74E-07 |
| Meis1 | 1.091130033 | 0.003643409 |
| Stard4 | 1.089123491 | 3.44E-05 |
| Thbs3 | 1.089033873 | 0.001522453 |
| Oaf | 1.088815238 | 6.55E-05 |
| Mns1 | 1.088774678 | 0.003004592 |
| Pkib | 1.088374991 | 2.19E-07 |
| Atf5 | 1.087894341 | 0.000305506 |
| Cib2 | 1.085911865 | 1.03E-05 |
| Slc25a42 | 1.08223431 | 0.004179317 |
| Zfp563 | 1.081050718 | 0.01490504 |
| Arhgap21 | 1.080644683 | 0.000102337 |
| Rapgef5 | 1.080622333 | 0.026284262 |
| Lrrc8b | 1.079387528 | 1.28E-06 |
| Ptgfrn | 1.079254113 | 7.41E-09 |
| Ptgr1 | 1.079126652 | 1.76E-05 |
| Qpct | 1.078995912 | 0.029032336 |
| Dtl | 1.078927631 | 0.002609059 |
| Gm37795 | 1.076993587 | 8.01E-06 |
| Peli3 | 1.07679358 | 0.00094957 |
| Mmgt1 | 1.075913852 | 3.53E-08 |
| St6gal1 | 1.073642077 | 1.60E-07 |
| Pdpn | 1.072446688 | 0.000334312 |
| Sec14l3 | 1.071977961 | 0.046364221 |
| Spats2 | 1.070430168 | 0.014444528 |
| Slc39a14 | 1.069464282 | 2.73E-12 |
| Apex1 | 1.068427409 | 4.65E-06 |
| Slc26a6 | 1.067338211 | 0.006107073 |
| Etfbkmt | 1.064615239 | 0.013570188 |
| Plxdc1 | 1.064241222 | 0.009551678 |
| Tjp2 | 1.062648032 | 0.000266048 |
| Ap5s1 | 1.062388208 | 8.22E-09 |
| Bcam | 1.061892444 | 0.023716248 |
| Acvr1 | 1.060738338 | 2.84E-07 |
| Mettl13 | 1.060339514 | 3.61E-05 |
| Snx1 | 1.060000351 | 7.66E-14 |
| Slc39a2 | 1.058508655 | 0.003191913 |
| Aco1 | 1.055540641 | 4.36E-06 |
| Zfp651 | 1.05546342 | 0.00997524 |
| Ptpn3 | 1.055292958 | 0.000311415 |
| Nanos1 | 1.054704189 | 0.018689636 |
| Dync2i2 | 1.054373402 | 0.009986264 |
| Ppp2r3a | 1.053910012 | 0.001242407 |
| Cryz | 1.052728749 | 0.008757339 |
| Brca1 | 1.052178182 | 0.010162314 |
| Fbf1 | 1.05204182 | 1.83E-07 |
| Zfta | 1.051885656 | 0.049853894 |
| Phf19 | 1.050699665 | 0.018127942 |
| Creg2 | 1.049500117 | 1.43E-06 |
| Decr1 | 1.048487162 | 0.002144996 |
| Mtmr7 | 1.04813844 | 6.40E-07 |
| Ppip5k1 | 1.045512955 | 3.88E-07 |
| Hook1 | 1.044361893 | 0.003332565 |
| Parp16 | 1.044264378 | 0.002788823 |
| Tmem154 | 1.042270354 | 7.70E-08 |
| Simc1 | 1.041761714 | 2.77E-05 |
| Ajuba | 1.041519966 | 0.007622882 |
| Supt3 | 1.040207091 | 0.006558116 |
| Cdr2 | 1.039103873 | 0.000388259 |
| Raet1e | 1.033896991 | 0.014571885 |
| Septin10 | 1.033352682 | 0.000105996 |
| Mybl2 | 1.032839148 | 0.005733861 |
| Slc36a4 | 1.02897902 | 4.80E-06 |
| Blvra | 1.028555417 | 3.29E-09 |
| Tsen2 | 1.028531327 | 0.010333685 |
| Ankmy2 | 1.027964709 | 0.000256298 |
| Ear2 | 1.027672376 | 0.000929488 |
| Atrnl1 | 1.027547133 | 3.82E-13 |
| Plag1 | 1.021875158 | 0.014152321 |
| Btbd6 | 1.02173359 | 0.002002649 |
| Firrm | 1.021600629 | 0.048948569 |
| H1f0 | 1.018045343 | 3.29E-09 |
| Get1 | 1.016209684 | 7.25E-05 |
| Tipin | 1.012469311 | 0.00875717 |
| Arhgap19 | 1.012277862 | 5.99E-08 |
| Hells | 1.012012954 | 3.34E-06 |
| Bbs7 | 1.011509692 | 0.03347346 |
| Gpt2 | 1.010501076 | 2.52E-05 |
| Anxa2 | 1.008445011 | 3.08E-13 |
| Trip13 | 1.00808341 | 0.000764073 |
| Atad2 | 1.00616357 | 8.86E-06 |
| Psat1 | 1.004455152 | 0.000998688 |
| Prim1 | 1.004451445 | 0.009111217 |
| Card11 | 1.004158185 | 1.73E-06 |
| Fabp5 | 1.001049361 | 3.42E-06 |
| Frmd4a | 1.00070207 | 0.001202175 |
| Gins1 | 1.000504537 | 0.003109489 |
