# Supplementary material for: Lung‐delivered IL‐10 mitigates Lung inflammation induced by repeated endotoxin exposures in male mice
Source: Physiol Rep. 2025 Feb 20;13(4):e70253. doi: 10.14814/phy2.70253 (PMC11842461; doi:10.14814/phy2.70253)
Supplement: Supplementary file 3 — Appendix S2. [file PHY2-13-e70253-s004.docx]

| **IL-10 vs. Vehicle Treatment in LPS-Exposed Mice** | | |
| --- | --- | --- |
| **Downregulated, adjusted p-value <0.05** | | |
| **Gene name** | **log2FoldChange** | **p-value adj** |
| Ubd | -8.422773246 | 6.53E-10 |
| Tgtp1 | -7.699330313 | 1.31E-125 |
| Tmem45a2 | -7.650536124 | 5.21E-06 |
| Sectm1a | -6.535257286 | 0.000167952 |
| Gm5970 | -6.414606985 | 5.27E-07 |
| Iigp1 | -6.403269583 | 3.22E-96 |
| Serpina3f | -6.265540965 | 1.74E-64 |
| Cxcl11 | -6.247927424 | 2.83E-17 |
| Ly6i | -6.199045585 | 1.72E-163 |
| Gzmf | -6.10241779 | 0.001382794 |
| Gm18752 | -6.087426868 | 0.0003146 |
| Orm2 | -6.059196139 | 0.000104773 |
| Gm6553 | -6.058314457 | 0.000435779 |
| Iigp1c | -6.047790001 | 1.44E-31 |
| Gm56557 | -6.03562688 | 0.000704552 |
| BC023105 | -5.962983947 | 8.79E-15 |
| Kcnab1 | -5.793582021 | 3.37E-08 |
| Stfa2 | -5.696957102 | 0.000247839 |
| Stfa3 | -5.681671109 | 1.05E-06 |
| Gm56880 | -5.622215815 | 0.002069651 |
| F830016B08Rik | -5.562416772 | 6.16E-14 |
| Batf2 | -5.438255532 | 2.25E-115 |
| Gm57062 | -5.409037504 | 0.011372471 |
| Gpr31a | -5.346890491 | 3.95E-08 |
| Tgtp2 | -5.326033787 | 1.06E-138 |
| Gm34917 | -5.321248322 | 1.49E-06 |
| Nos2 | -5.308423072 | 8.67E-68 |
| Gm13183 | -5.281362279 | 0.009459886 |
| Il18bp | -5.269080633 | 1.15E-203 |
| Hif3a | -5.264726853 | 0.000231773 |
| Prm1 | -5.264111686 | 0.019690985 |
| Or2v1 | -5.22924238 | 1.20E-71 |
| Gbp5 | -5.189219056 | 3.98E-104 |
| Gbp4 | -5.176147435 | 2.75E-186 |
| Cxcl9 | -5.164174383 | 8.13E-15 |
| Gm12185 | -5.143142248 | 5.20E-35 |
| Gm15056 | -5.127440538 | 2.22E-10 |
| Gm12250 | -5.103596735 | 4.65E-112 |
| Clvs1 | -5.099098025 | 3.36E-06 |
| Gm38832 | -5.069509551 | 0.0036714 |
| Cyp2b9 | -5.00170791 | 0.019051176 |
| Sectm1b | -4.908129184 | 0.014457806 |
| Gpr31b | -4.906706343 | 1.32E-23 |
| Cxcl10 | -4.877215253 | 1.49E-51 |
| Gm32296 | -4.865686931 | 0.011435756 |
| Gbp2 | -4.855081289 | 2.17E-158 |
| Trim30c | -4.852984405 | 5.26E-55 |
| 2310043L19Rik | -4.849013546 | 0.039916943 |
| Ly6c2 | -4.839378561 | 1.15E-165 |
| Ptx3 | -4.82899187 | 0.000910723 |
| Slamf8 | -4.824034957 | 5.40E-67 |
| Gm4841 | -4.796463315 | 3.92E-06 |
| Clic5 | -4.795463947 | 2.67E-66 |
| Gm807 | -4.795392312 | 6.77E-12 |
| Calhm6 | -4.79335322 | 2.93E-54 |
| Gm44101 | -4.787649831 | 0.007499326 |
| Serpina3i | -4.738052692 | 0.028245103 |
| Fcnb | -4.712866215 | 0.008175417 |
| Ifit2 | -4.669823677 | 8.24E-54 |
| Unc5c | -4.669437285 | 0.009527872 |
| Gm42648 | -4.659231202 | 0.048612594 |
| Ly6a2 | -4.612979259 | 1.29E-43 |
| Stfa1 | -4.529738726 | 1.23E-07 |
| Foxr1 | -4.452782557 | 0.041917019 |
| Gp6 | -4.449672882 | 0.000147625 |
| Btnl4 | -4.427402579 | 0.012763425 |
| Gm12187 | -4.420341404 | 0.00413791 |
| Flacc1 | -4.400496823 | 0.027691046 |
| Gm6093 | -4.393672842 | 0.027672722 |
| Ly6a | -4.389046583 | 6.92E-67 |
| Ifi44 | -4.381131698 | 0.000245311 |
| Gm57332 | -4.378257809 | 0.028437853 |
| Gm49205 | -4.376172712 | 0.024532437 |
| Ifi47 | -4.374443454 | 9.47E-113 |
| Gm48857 | -4.3635473 | 0.031257615 |
| Inpp5j | -4.341365604 | 1.34E-45 |
| Crispld2 | -4.329701447 | 0.000426502 |
| Gvin-ps4 | -4.295525842 | 1.86E-22 |
| Gm9845 | -4.277516138 | 5.01E-08 |
| St3gal5 | -4.250201436 | 7.61E-49 |
| Irgm1 | -4.239092904 | 3.25E-122 |
| Stfa2l1 | -4.234942204 | 2.84E-07 |
| Ifit3 | -4.229160766 | 1.12E-42 |
| Majin | -4.201305238 | 6.55E-05 |
| Cstdc5 | -4.195757127 | 4.79E-05 |
| Isg15 | -4.160261486 | 3.12E-54 |
| Igtp | -4.131226239 | 6.32E-102 |
| ENSMUSG00000121828 | -4.088125738 | 1.61E-36 |
| Il12a | -4.083609165 | 5.34E-20 |
| Gm5431 | -4.044407171 | 1.30E-74 |
| Gm17034 | -4.042167391 | 0.018081744 |
| Serpina3h | -4.023576166 | 0.000498923 |
| Gm34316 | -4.022044326 | 7.45E-05 |
| Rtp4 | -4.017544211 | 5.00E-52 |
| Ifi205 | -4.009837731 | 5.35E-58 |
| Ifit3b | -3.989508799 | 1.33E-35 |
| Cstdc4 | -3.967948594 | 6.74E-05 |
| Saa2 | -3.960892032 | 0.03340865 |
| Gbp9 | -3.941102186 | 1.77E-153 |
| Xaf1 | -3.935291951 | 1.89E-66 |
| Gzmc | -3.929287349 | 3.05E-14 |
| Xcl1 | -3.926494225 | 7.05E-32 |
| Oasl1 | -3.923592438 | 6.60E-33 |
| Plac8 | -3.909611828 | 3.03E-72 |
| Gm45418 | -3.907756435 | 0.000109559 |
| Gm2629 | -3.906592987 | 1.55E-06 |
| Gm20662 | -3.902641721 | 2.93E-05 |
| Gbp2b | -3.893947988 | 1.09E-12 |
| Gm17334 | -3.885548102 | 0.021313793 |
| Hsd17b2 | -3.881337912 | 0.025437414 |
| Ly6g | -3.874927031 | 2.88E-09 |
| Gm28177 | -3.837484387 | 2.05E-08 |
| Serpina3g | -3.835922012 | 1.40E-61 |
| Pla2g7 | -3.821532635 | 1.08E-46 |
| Gm27252 | -3.798294748 | 0.000734239 |
| Ifit1 | -3.789833153 | 3.09E-34 |
| Il22 | -3.779316966 | 0.015204616 |
| Selenom | -3.777915835 | 2.90E-36 |
| Rsad2 | -3.775963221 | 3.48E-44 |
| Gm5535 | -3.773914558 | 0.011472645 |
| Cabp4 | -3.753752402 | 5.46E-05 |
| Apoc4 | -3.750350598 | 0.036432433 |
| Gm10030 | -3.747077738 | 0.008599072 |
| Irf7 | -3.726598236 | 5.43E-31 |
| Gm57308 | -3.711413843 | 0.003081091 |
| Gm48277 | -3.708325244 | 4.41E-06 |
| Zbp1 | -3.678936011 | 9.64E-73 |
| Gbp6 | -3.666864426 | 1.98E-67 |
| Btnl6 | -3.660259735 | 0.00892604 |
| Mrgpra2a | -3.650805725 | 7.16E-25 |
| Ifng | -3.6433371 | 1.56E-29 |
| Gbp3 | -3.637869929 | 4.52E-56 |
| Ms4a4c | -3.568425968 | 1.11E-40 |
| Gm35551 | -3.564028675 | 0.006516618 |
| Ifitm3 | -3.560224115 | 1.64E-50 |
| AW112010 | -3.559909723 | 5.13E-132 |
| Gm56595 | -3.536416299 | 0.012329142 |
| Oasl2 | -3.497557408 | 2.37E-49 |
| Csta3 | -3.4908192 | 1.66E-05 |
| Tmem178 | -3.467727358 | 0.00212199 |
| Sertad4 | -3.455293539 | 0.021091587 |
| F730016J06Rik | -3.436489664 | 3.64E-05 |
| Gm19684 | -3.434526141 | 3.57E-08 |
| Irgm2 | -3.412724411 | 1.02E-89 |
| Asb11 | -3.410208386 | 4.59E-06 |
| Il12b | -3.406895775 | 1.91E-09 |
| Irf1 | -3.402373771 | 6.13E-56 |
| Gm36723 | -3.401338433 | 0.000233765 |
| Usp18 | -3.342103089 | 1.28E-38 |
| Spon1 | -3.337954798 | 6.07E-36 |
| Gm55515 | -3.337871475 | 6.10E-05 |
| Isg20 | -3.334599906 | 2.74E-36 |
| 4933412E12Rik | -3.332446074 | 4.67E-30 |
| Gm31583 | -3.331004838 | 2.00E-05 |
| Ankrd1 | -3.32852004 | 0.015720963 |
| Itgb2l | -3.32272384 | 2.77E-11 |
| Aif1 | -3.316522848 | 1.70E-42 |
| Mrgpra2b | -3.315203937 | 1.13E-16 |
| Clec2g | -3.311442165 | 0.014538631 |
| U90926 | -3.298915828 | 3.76E-05 |
| Ifi206 | -3.279967321 | 1.21E-36 |
| Il12rb1 | -3.276509202 | 1.17E-38 |
| Notch4 | -3.268205976 | 1.06E-08 |
| Il27 | -3.238145577 | 2.36E-15 |
| Susd2 | -3.237424908 | 1.26E-32 |
| Chi3l1 | -3.220850065 | 6.95E-18 |
| Oas3 | -3.196529648 | 6.23E-22 |
| Tap1 | -3.185494877 | 2.71E-86 |
| Gbp10 | -3.183417002 | 9.54E-22 |
| Smim38 | -3.181668156 | 0.01046684 |
| Csf2 | -3.177954864 | 0.002894496 |
| Cd40 | -3.175876646 | 7.13E-21 |
| Cmpk2 | -3.164372628 | 4.39E-28 |
| Ccl5 | -3.155918426 | 5.00E-52 |
| Tuba8 | -3.152219329 | 3.15E-11 |
| Gm11827 | -3.151954376 | 0.000198537 |
| Gm35279 | -3.145790786 | 0.039957693 |
| Ddx60 | -3.123161059 | 9.72E-73 |
| Apol9b | -3.120453945 | 0.000440689 |
| Dnase1l3 | -3.11067562 | 1.19E-11 |
| Scrg1 | -3.10993648 | 0.021532785 |
| Tnc | -3.097828198 | 0.00034797 |
| Scarf1 | -3.078616957 | 4.46E-35 |
| Adgrb1 | -3.0773111 | 1.63E-09 |
| Ifi213 | -3.061358503 | 2.22E-24 |
| Prnp | -3.038155541 | 3.73E-06 |
| H2-T24 | -3.038114747 | 9.23E-33 |
| Ifi204 | -3.029165047 | 1.53E-54 |
| Gbp11 | -3.026567354 | 3.27E-11 |
| Wnk2 | -3.024345873 | 5.03E-05 |
| Pik3ip1 | -3.023991031 | 0.000525866 |
| Acod1 | -3.006819373 | 1.54E-26 |
| Klra6 | -3.002432855 | 0.00200475 |
| Slfn4 | -2.9977623 | 9.52E-25 |
| Ido1 | -2.990059269 | 0.002899194 |
| Ano7 | -2.988272838 | 0.012393918 |
| Fap | -2.982465267 | 9.06E-07 |
| 4933427D14Rik | -2.979610381 | 0.012266807 |
| Mybph | -2.971665566 | 1.23E-12 |
| Gm12758 | -2.962488497 | 0.038111358 |
| H2-Q6 | -2.96131009 | 8.62E-59 |
| Gm2808 | -2.956559998 | 4.73E-12 |
| Socs1 | -2.951913496 | 2.38E-19 |
| P2rx3 | -2.949651609 | 0.003595197 |
| Ifi211 | -2.946043664 | 1.17E-38 |
| Orm1 | -2.941869012 | 8.44E-12 |
| Sp140l1 | -2.939234825 | 6.68E-16 |
| Vwa3b | -2.93301237 | 0.005034003 |
| Gm8752 | -2.932893596 | 1.00E-18 |
| Lipg | -2.919164449 | 7.94E-13 |
| Plekhs1 | -2.911467214 | 0.019190773 |
| S100a9 | -2.907662919 | 0.000364555 |
| Stat1 | -2.907004625 | 3.60E-67 |
| Zfr2 | -2.906464703 | 0.013650326 |
| Ifi208 | -2.901359206 | 5.59E-25 |
| Il15ra | -2.898427773 | 8.24E-54 |
| 1700071M16Rik | -2.896914589 | 2.37E-08 |
| Oas1g | -2.884303362 | 8.74E-19 |
| Btbd16 | -2.87685941 | 6.37E-07 |
| Gm34680 | -2.875485822 | 1.73E-06 |
| Herc6 | -2.87471847 | 3.78E-39 |
| Ube2l6 | -2.873202003 | 4.70E-64 |
| Gm47727 | -2.863730441 | 0.024877534 |
| Retnlg | -2.854408096 | 1.30E-05 |
| Gm8000 | -2.853649952 | 0.000516142 |
| S100a8 | -2.84763883 | 0.001943388 |
| Parp10 | -2.845883146 | 1.72E-61 |
| Gm49751 | -2.837477071 | 3.88E-07 |
| Gm29721 | -2.828764405 | 0.021806818 |
| Gm12216 | -2.815622894 | 9.56E-07 |
| Gbp7 | -2.811252622 | 2.38E-107 |
| ENSMUSG00000121434 | -2.804112773 | 0.025882245 |
| Phf11a | -2.802959493 | 4.83E-08 |
| Pram1 | -2.788820831 | 0.00031278 |
| Tnfsf4 | -2.7858816 | 9.38E-06 |
| Trpm2 | -2.776395242 | 1.36E-34 |
| Gm43802 | -2.764640182 | 9.30E-17 |
| Cxcl13 | -2.762907458 | 1.89E-08 |
| Prr29 | -2.75410664 | 0.040832106 |
| Hmga1b | -2.749449351 | 0.030975981 |
| Nlrc5 | -2.748335429 | 3.63E-63 |
| Gm45193 | -2.747905299 | 7.65E-14 |
| Krt86 | -2.743672579 | 0.002125563 |
| Slamf7 | -2.732563415 | 6.62E-53 |
| H2-Q7 | -2.732465069 | 8.94E-45 |
| Mx1 | -2.717781532 | 0.001795889 |
| Gbp8 | -2.716185402 | 3.43E-21 |
| Lrrc4 | -2.715768295 | 1.09E-10 |
| Gvin-ps2 | -2.714777217 | 1.47E-26 |
| Gm57206 | -2.713105936 | 0.000630666 |
| Gm29719 | -2.711508924 | 0.000478729 |
| H2-Q4 | -2.70610021 | 1.17E-58 |
| Gm36634 | -2.701357795 | 0.042630733 |
| Gm49391 | -2.698864498 | 2.53E-11 |
| Pglyrp1 | -2.682715284 | 6.55E-05 |
| Slfn5 | -2.669114422 | 6.87E-27 |
| Lcn2 | -2.667958362 | 1.98E-05 |
| Phf11d | -2.667771275 | 6.78E-15 |
| H2-Q5 | -2.659026276 | 7.05E-30 |
| Hrh2 | -2.647699945 | 7.49E-15 |
| Tnfrsf14 | -2.63287642 | 6.64E-50 |
| Mx2 | -2.630591706 | 1.48E-08 |
| Gm266 | -2.629342605 | 0.000893886 |
| Ifi209 | -2.626203898 | 3.12E-45 |
| Tnfsf15 | -2.6260878 | 1.61E-05 |
| Psmb9 | -2.619486118 | 9.88E-67 |
| Gm57225 | -2.612896695 | 3.57E-08 |
| Gm56817 | -2.611139827 | 1.81E-09 |
| Klra1 | -2.610685464 | 1.09E-05 |
| Gm8091 | -2.604378911 | 0.034912984 |
| Saa1 | -2.597575098 | 0.011029832 |
| Ifitm6 | -2.585703848 | 1.29E-10 |
| Fcgr4 | -2.565567498 | 2.08E-28 |
| Ctla2b | -2.559987748 | 1.83E-11 |
| Ankrd66 | -2.557114047 | 2.75E-08 |
| Il36a | -2.551190007 | 0.000984287 |
| Gm19696 | -2.544623038 | 2.77E-05 |
| Obscn | -2.541913594 | 3.68E-06 |
| Parp14 | -2.541122385 | 4.12E-41 |
| 1700047M11Rik | -2.537530619 | 0.018127942 |
| Camp | -2.5356205 | 6.33E-07 |
| Phf11b | -2.526756062 | 1.63E-13 |
| Ntng2 | -2.524399192 | 3.75E-12 |
| Mirt2 | -2.512069666 | 2.78E-12 |
| Gm56663 | -2.510920862 | 2.76E-07 |
| Gm45014 | -2.506699748 | 0.043855285 |
| Gm57045 | -2.503329457 | 0.011899606 |
| Gpr18 | -2.496866049 | 1.69E-31 |
| Msantd3 | -2.491646734 | 1.13E-15 |
| Trim21 | -2.481360737 | 1.74E-26 |
| Ppm1n | -2.480229688 | 6.02E-12 |
| Tbx10 | -2.476231077 | 0.04719191 |
| Uba7 | -2.472689658 | 5.31E-61 |
| Ifi27l2a | -2.472116588 | 1.77E-14 |
| Trem3 | -2.461521959 | 2.47E-10 |
| Ms4a6b | -2.450805968 | 1.90E-29 |
| H2-T22 | -2.446807731 | 8.48E-56 |
| Gm56922 | -2.442544313 | 0.042755692 |
| Mapk13 | -2.434911577 | 2.55E-07 |
| F3 | -2.431115341 | 9.92E-19 |
| 9530082P21Rik | -2.427586207 | 4.20E-10 |
| Apoc2 | -2.421809781 | 1.51E-31 |
| 9830107B12Rik | -2.420756512 | 7.44E-06 |
| Trex1 | -2.420137457 | 5.44E-30 |
| Bcl2l14 | -2.420043333 | 0.000112008 |
| Slfn1 | -2.414923528 | 6.51E-15 |
| 4930438A08Rik | -2.399183319 | 0.013650326 |
| Gm36161 | -2.396780443 | 2.93E-34 |
| Ifit1bl1 | -2.394351691 | 6.80E-08 |
| Gm49510 | -2.393258324 | 8.93E-06 |
| Tap2 | -2.392192471 | 5.81E-46 |
| Psmb8 | -2.387065796 | 3.45E-44 |
| Gm21762 | -2.386925925 | 1.89E-07 |
| Gm56974 | -2.384040415 | 0.001556846 |
| Dtx3l | -2.373098291 | 2.28E-41 |
| Enpp5 | -2.367397997 | 4.40E-26 |
| Slc44a3 | -2.365511819 | 0.03883228 |
| Asprv1 | -2.363759438 | 0.001370779 |
| Ly6m | -2.358113963 | 0.003676267 |
| Gpr141 | -2.350024091 | 1.56E-36 |
| Prdx5 | -2.349566758 | 4.75E-25 |
| Arhgef37 | -2.346204175 | 1.64E-30 |
| Tmem171 | -2.343934413 | 0.000247184 |
| Spatc1 | -2.340331514 | 0.000860206 |
| Serpine2 | -2.335019059 | 8.95E-05 |
| B230208H11Rik | -2.332798342 | 0.004527865 |
| Slfn8 | -2.331715988 | 3.06E-46 |
| Gm14010 | -2.328592629 | 0.041234983 |
| Gm13822 | -2.321013168 | 0.000151446 |
| Mmp8 | -2.315314271 | 0.015235783 |
| 6530402F18Rik | -2.311785423 | 2.09E-11 |
| Icam4 | -2.304809274 | 0.000115219 |
| Gm16192 | -2.301837745 | 0.000727917 |
| Nlrp1c-ps | -2.299199741 | 3.31E-06 |
| Gvin2 | -2.298571255 | 2.48E-25 |
| Rnd1 | -2.298148547 | 3.77E-09 |
| H2-T23 | -2.296057404 | 1.40E-34 |
| Lta | -2.285416067 | 0.00020767 |
| BC147527 | -2.284965878 | 0.00191743 |
| Gvin1 | -2.283525879 | 4.84E-25 |
| Ripor3 | -2.277676024 | 0.046392863 |
| Klra17 | -2.269926339 | 1.91E-05 |
| Stat2 | -2.261217825 | 2.15E-35 |
| Csta2 | -2.259110559 | 0.000110447 |
| Gm2011 | -2.256369496 | 0.000499869 |
| Tmprss4 | -2.255366716 | 0.020575801 |
| Adgrg3 | -2.241162624 | 0.036879134 |
| Cst7 | -2.239765316 | 6.37E-15 |
| Oas1b | -2.23622338 | 2.48E-13 |
| ENSMUSG00000121501 | -2.234595012 | 1.15E-31 |
| H2-T10 | -2.229785539 | 6.39E-28 |
| Zfp811 | -2.22923307 | 1.88E-05 |
| Gm16675 | -2.222272553 | 1.28E-12 |
| Gm6545 | -2.213386551 | 5.43E-05 |
| Gm20234 | -2.205452962 | 0.00115076 |
| Layn | -2.197360485 | 4.44E-05 |
| Gm8750 | -2.193554654 | 0.024549482 |
| 4930599N23Rik | -2.1933707 | 0.000187914 |
| Gm10425 | -2.191549243 | 5.35E-05 |
| Upp1 | -2.189002124 | 3.74E-11 |
| Gm57169 | -2.177256923 | 0.028519674 |
| Tmem40 | -2.168389886 | 0.000171965 |
| Chrm3 | -2.165991331 | 1.19E-06 |
| Cd274 | -2.16442897 | 6.52E-26 |
| Cysltr2 | -2.162966444 | 2.17E-10 |
| Gm12107 | -2.162796025 | 0.007808465 |
| Gm15753 | -2.161585592 | 0.012212249 |
| E230001N04Rik | -2.158378559 | 0.019769836 |
| 9330175E14Rik | -2.155510786 | 5.91E-10 |
| Tapbpl | -2.152559027 | 2.20E-34 |
| Gpr84 | -2.149857144 | 3.26E-10 |
| Ms4a4a | -2.144713749 | 8.86E-11 |
| Lacc1 | -2.141641171 | 2.43E-12 |
| C1qtnf6 | -2.136737027 | 6.50E-06 |
| Clnk | -2.136330619 | 6.67E-06 |
| Schip1 | -2.131140153 | 1.49E-08 |
| Psme2 | -2.125720139 | 1.43E-31 |
| Rnf213 | -2.121125271 | 2.54E-12 |
| Ikbke | -2.119785611 | 1.33E-08 |
| Lst1 | -2.118449703 | 4.58E-20 |
| Adgb | -2.116725494 | 2.41E-28 |
| B430306N03Rik | -2.10210178 | 2.39E-12 |
| Gm37988 | -2.09582656 | 0.038576975 |
| D730005E14Rik | -2.087091035 | 0.038074896 |
| Lpar1 | -2.083904691 | 0.004136746 |
| Dhx58 | -2.067844431 | 3.24E-17 |
| Trim30a | -2.063948381 | 4.65E-24 |
| Wnt6 | -2.056876342 | 0.013780003 |
| Kcnj10 | -2.056486839 | 0.00011189 |
| Slc15a2 | -2.055924572 | 0.000312136 |
| Clec4a1 | -2.054102823 | 1.41E-35 |
| Tnfsf13b | -2.053840759 | 3.01E-21 |
| Gm9522 | -2.041614263 | 0.003868369 |
| Trafd1 | -2.04057618 | 1.97E-26 |
| Oas2 | -2.03944299 | 4.76E-21 |
| Abcc3 | -2.037875549 | 5.13E-16 |
| Gm57070 | -2.037736885 | 0.025503523 |
| Prss50 | -2.036217501 | 0.043615897 |
| Gm32184 | -2.031603098 | 0.04891862 |
| Olfml2b | -2.019498953 | 6.93E-10 |
| H2-K1 | -2.01476083 | 1.15E-49 |
| Ccnjl | -2.012876836 | 0.044152727 |
| Tnfsf10 | -2.009575533 | 3.97E-06 |
| Gatm | -2.008727452 | 5.94E-27 |
| Htr7 | -2.00615561 | 4.37E-20 |
| Ptges | -2.005626031 | 7.63E-09 |
| Nmi | -2.004169604 | 5.36E-31 |
| Slc28a2 | -2.002628381 | 1.65E-24 |
| ENSMUSG00000121359 | -2.001391222 | 0.005072243 |
| Ap1m2 | -1.998807669 | 0.000723969 |
| Adora2a | -1.997036765 | 3.53E-12 |
| Pcdh7 | -1.993981714 | 4.27E-08 |
| Cyfip2 | -1.993337266 | 1.49E-24 |
| Clec4a4 | -1.990992922 | 0.002958908 |
| Sp140l2 | -1.98683824 | 3.34E-13 |
| Arhgef10l | -1.986259979 | 1.06E-13 |
| Pde8b | -1.984950684 | 0.006555467 |
| Ctrl | -1.984229394 | 0.001680292 |
| Klra7 | -1.983817057 | 4.43E-08 |
| Adamts7 | -1.981583324 | 0.013301972 |
| Grina | -1.979851756 | 2.59E-25 |
| Tlr12 | -1.968459974 | 0.000501487 |
| Gm19705 | -1.96678235 | 3.47E-07 |
| Slc2a6 | -1.962100612 | 2.59E-25 |
| Gm56957 | -1.956735172 | 2.58E-05 |
| Il1r2 | -1.949831103 | 0.030297775 |
| Vcam1 | -1.940689452 | 7.10E-06 |
| Khdc3 | -1.938597382 | 0.007808465 |
| Ypel1 | -1.937285687 | 0.007691128 |
| Edn1 | -1.935790497 | 0.000432858 |
| Rap1gap2 | -1.935270993 | 3.54E-19 |
| Cpne2 | -1.933739957 | 7.76E-09 |
| Cp | -1.93348953 | 3.73E-15 |
| Gm41442 | -1.932750571 | 0.041230258 |
| Sptbn2 | -1.9288919 | 0.045947223 |
| Fgl2 | -1.928251511 | 2.08E-16 |
| Ccdc88b | -1.927549035 | 2.85E-17 |
| Slc24a3 | -1.926023374 | 0.000189343 |
| Adssl1 | -1.924589004 | 2.05E-10 |
| Fcgr1 | -1.924421662 | 1.26E-21 |
| 1600010M07Rik | -1.923728369 | 5.79E-05 |
| Tmem119 | -1.922896522 | 6.57E-10 |
| Parp12 | -1.915178738 | 9.02E-31 |
| Cd180 | -1.913715061 | 2.54E-19 |
| Psmb10 | -1.912846579 | 4.00E-27 |
| Tagap | -1.909501874 | 6.56E-24 |
| 1700011B04Rik | -1.907199718 | 0.016654331 |
| Ifitm1 | -1.907008903 | 1.12E-06 |
| Gvin3 | -1.906907442 | 2.39E-30 |
| Tmeff1 | -1.905773373 | 8.40E-15 |
| Marcksl1 | -1.903103902 | 8.34E-09 |
| Cmklr1 | -1.898009737 | 8.73E-30 |
| Vnn3 | -1.895299407 | 8.05E-08 |
| G0s2 | -1.893223944 | 3.86E-06 |
| Gm56872 | -1.887871508 | 0.000953585 |
| Slc6a13 | -1.886672974 | 0.000396867 |
| Gm15964 | -1.886027477 | 3.21E-07 |
| Fpr2 | -1.883106424 | 1.86E-10 |
| Draxin | -1.881738646 | 0.020285286 |
| Gm21188 | -1.880990063 | 6.55E-34 |
| Hpse | -1.879894089 | 5.46E-18 |
| Atp2b4 | -1.874068637 | 1.85E-20 |
| Nyap1 | -1.87332907 | 4.12E-05 |
| Tlr1 | -1.872759273 | 1.43E-27 |
| Krt83 | -1.872245652 | 0.008364073 |
| Oas1a | -1.872195538 | 8.45E-15 |
| Foxl3 | -1.871405401 | 0.007920868 |
| Tifa | -1.867849743 | 5.46E-18 |
| Nod2 | -1.866483207 | 1.04E-14 |
| Kalrn | -1.864777177 | 9.44E-06 |
| Mmp9 | -1.86105414 | 7.53E-07 |
| Limd2 | -1.860606626 | 9.85E-18 |
| Serpinb9b | -1.85666062 | 1.11E-07 |
| Ebi3 | -1.853428879 | 1.01E-06 |
| Tapbp | -1.851915787 | 2.64E-26 |
| Samhd1 | -1.848559901 | 2.60E-32 |
| Slc6a19 | -1.848323623 | 0.003757789 |
| Sfmbt2 | -1.846216964 | 1.72E-07 |
| Ifi203-ps | -1.843334257 | 0.03464662 |
| Ninj1 | -1.842956478 | 3.31E-10 |
| Irf9 | -1.83422709 | 8.01E-22 |
| Dntt | -1.83383275 | 0.004838689 |
| Gm11131 | -1.833779964 | 4.18E-13 |
| Gm28068 | -1.833712059 | 0.003982967 |
| Gm46828 | -1.832428466 | 0.032130426 |
| Lzts1 | -1.831594935 | 0.010825375 |
| C1s1 | -1.826989397 | 3.46E-05 |
| H2-Ab1 | -1.822233874 | 0.027104468 |
| Slc11a1 | -1.822212098 | 6.91E-10 |
| Vill | -1.818956759 | 1.24E-10 |
| Entpd1 | -1.81742993 | 1.13E-22 |
| Treml2 | -1.816473489 | 1.81E-15 |
| Cnn3 | -1.80966086 | 5.38E-08 |
| Ildr1 | -1.809475423 | 2.14E-05 |
| Il10 | -1.807206261 | 0.002928463 |
| Gm7609 | -1.800466064 | 0.001759603 |
| Klk1 | -1.800333662 | 0.000110447 |
| Gm16094 | -1.79572025 | 0.033500346 |
| Lair1 | -1.793605322 | 1.12E-27 |
| Gpr65 | -1.793266045 | 2.00E-37 |
| Gm12840 | -1.791589111 | 4.41E-06 |
| Ly6e | -1.790280111 | 1.30E-30 |
| Cd72 | -1.788727748 | 8.04E-11 |
| Psme2b | -1.787819987 | 1.60E-12 |
| Qrfp | -1.787171203 | 0.006171208 |
| Trim30b | -1.787050307 | 9.76E-16 |
| Slco3a1 | -1.785295594 | 1.74E-15 |
| Treml4 | -1.784530267 | 7.35E-09 |
| Gm33699 | -1.781543015 | 0.040482304 |
| Gm44187 | -1.779684627 | 0.010685446 |
| Bst2 | -1.777210857 | 9.33E-08 |
| Cd38 | -1.775270298 | 1.07E-12 |
| Gm20236 | -1.769211394 | 0.021964846 |
| Gm37094 | -1.769211394 | 0.021964846 |
| Gm13012 | -1.76794751 | 1.44E-05 |
| Htra1 | -1.767732262 | 6.83E-13 |
| Ocstamp | -1.76760547 | 0.020467854 |
| Saa3 | -1.766928493 | 2.04E-07 |
| Ldhb | -1.766242089 | 1.40E-08 |
| Gm16464 | -1.76569389 | 4.15E-06 |
| Sell | -1.75712182 | 2.18E-11 |
| Prf1 | -1.753341745 | 1.01E-08 |
| Gm56551 | -1.750957512 | 0.00803398 |
| Lipe | -1.750179471 | 3.67E-11 |
| Dapk2 | -1.750111405 | 2.18E-06 |
| Cpq | -1.74972775 | 5.46E-06 |
| Gvin-ps7 | -1.749375702 | 1.16E-26 |
| Sytl3 | -1.74820436 | 6.74E-07 |
| Adam19 | -1.740062753 | 5.62E-13 |
| Psme1 | -1.730279593 | 1.60E-29 |
| Rassf2 | -1.729104457 | 5.79E-10 |
| Rcsd1 | -1.728091594 | 2.12E-08 |
| Adgrg5 | -1.727746479 | 3.61E-11 |
| Igf2bp1 | -1.723785351 | 0.011919867 |
| Cldn15 | -1.723590051 | 0.00061496 |
| Mefv | -1.723091331 | 6.11E-07 |
| Ckap4 | -1.722555537 | 1.41E-06 |
| Gm21011 | -1.719663382 | 0.040832106 |
| Ms4a6c | -1.714561526 | 7.71E-32 |
| Gpr141b | -1.714503178 | 2.60E-06 |
| Parp9 | -1.71028795 | 6.84E-25 |
| C1qb | -1.706956433 | 1.23E-19 |
| Gp1bb | -1.706902125 | 0.012535943 |
| Gp1ba | -1.704323247 | 0.03989494 |
| Sp100 | -1.704248587 | 2.55E-24 |
| Carmil1 | -1.704092022 | 8.08E-11 |
| Rab27a | -1.700071614 | 1.88E-11 |
| Gm17455 | -1.696995364 | 0.007907101 |
| Atp1a3 | -1.692675531 | 4.27E-40 |
| AU040972 | -1.688445831 | 0.000106907 |
| Stag3 | -1.684391558 | 0.016204672 |
| Mov10 | -1.682667321 | 3.43E-12 |
| Gm45700 | -1.68202935 | 0.000121554 |
| Il2ra | -1.681031754 | 4.14E-09 |
| Cd160 | -1.677933036 | 0.000871841 |
| Gramd2a | -1.675186816 | 0.00734626 |
| Ptpn18 | -1.67511298 | 5.48E-12 |
| Unc13d | -1.674696423 | 1.37E-25 |
| Ifi35 | -1.673663471 | 5.41E-14 |
| Itgb7 | -1.673090579 | 3.21E-33 |
| Slc39a4 | -1.671894188 | 3.07E-08 |
| Ogfr | -1.671707452 | 4.39E-16 |
| Tigit | -1.668287736 | 4.02E-06 |
| 1600022D10Rik | -1.665387067 | 0.000238999 |
| Cd52 | -1.66381895 | 4.03E-29 |
| Serpinb6b | -1.66253833 | 7.08E-11 |
| Cxcr2 | -1.661771842 | 0.014097003 |
| Stab1 | -1.661241336 | 1.48E-16 |
| Trim12a | -1.66107845 | 7.06E-17 |
| Ssc4d | -1.660304394 | 0.00039589 |
| Trim30d | -1.658440403 | 2.34E-15 |
| Osgin1 | -1.658225477 | 0.022013949 |
| Gsdmd | -1.656959767 | 8.91E-22 |
| Aoah | -1.652496767 | 1.80E-11 |
| Mmp25 | -1.651215761 | 4.31E-09 |
| Tlr9 | -1.65116814 | 1.54E-10 |
| Jaml | -1.646843584 | 1.65E-17 |
| A530040E14Rik | -1.640474199 | 0.016434276 |
| Spata13 | -1.638725339 | 3.51E-08 |
| Ifih1 | -1.632520506 | 5.40E-27 |
| Il2rb | -1.632480907 | 4.23E-12 |
| AA467197 | -1.63150556 | 1.68E-28 |
| Mlkl | -1.628551618 | 5.10E-10 |
| Epsti1 | -1.624778877 | 4.13E-21 |
| Gm19412 | -1.622439084 | 0.003831653 |
| Enpp4 | -1.621829088 | 3.05E-14 |
| Palm | -1.620955373 | 3.51E-12 |
| Hdc | -1.617895808 | 9.89E-08 |
| Hoxb3 | -1.613893202 | 0.001342634 |
| Kcnip3 | -1.613087019 | 0.013609759 |
| Cavin4 | -1.612039934 | 0.006549908 |
| Ccl12 | -1.608349526 | 0.026999799 |
| Adra2b | -1.60779522 | 0.006851518 |
| Tmem67 | -1.607160386 | 3.33E-07 |
| Oit3 | -1.604290685 | 0.000272041 |
| Gm20257 | -1.602959026 | 0.003591009 |
| Rdh12 | -1.601237951 | 0.000626354 |
| F830208F22Rik | -1.598153821 | 3.62E-05 |
| Prss46 | -1.597132901 | 7.42E-05 |
| Ifnlr1 | -1.595662708 | 0.00028138 |
| Trim56 | -1.593293818 | 6.08E-17 |
| Znfx1 | -1.590481404 | 2.31E-14 |
| Muc4 | -1.58724299 | 0.011571303 |
| Tnf | -1.587192142 | 1.80E-06 |
| Gm15417 | -1.586943594 | 0.004961704 |
| Gm8459 | -1.580924842 | 0.005572301 |
| AW011738 | -1.578811269 | 4.63E-11 |
| Ggt1 | -1.575072213 | 0.005337083 |
| Hk3 | -1.57303742 | 2.86E-19 |
| Tubb3 | -1.571902661 | 0.015856313 |
| Cd74 | -1.571901538 | 0.046443717 |
| Ncr1 | -1.571109445 | 4.15E-05 |
| Klhl6 | -1.568542862 | 4.66E-05 |
| Gm11714 | -1.567541814 | 0.027977351 |
| Txk | -1.566900103 | 6.35E-07 |
| Scnn1a | -1.564394356 | 9.87E-15 |
| Icam1 | -1.563398934 | 3.04E-13 |
| Arl5c | -1.56250726 | 1.04E-13 |
| H2-M3 | -1.560180494 | 7.63E-16 |
| Daxx | -1.557033876 | 7.95E-09 |
| Gm57204 | -1.554392252 | 3.72E-05 |
| AB124611 | -1.543270916 | 8.39E-16 |
| Gja1 | -1.541832773 | 0.005574635 |
| Crtam | -1.540249279 | 1.19E-07 |
| Olfml3 | -1.53883389 | 9.63E-07 |
| Inka1 | -1.537538671 | 0.000757309 |
| Susd3 | -1.530941791 | 6.53E-09 |
| Ctse | -1.524819501 | 0.00030929 |
| Exoc3l4 | -1.524383605 | 6.86E-06 |
| Rasa4 | -1.524072204 | 9.29E-23 |
| Gm56993 | -1.522742485 | 0.033224575 |
| Il36g | -1.522410294 | 0.000127564 |
| B2m | -1.522244855 | 1.21E-36 |
| Serpinb9 | -1.518283849 | 2.81E-13 |
| Rasgrp1 | -1.514196901 | 6.21E-17 |
| 9930111J21Rik1 | -1.512404862 | 1.99E-19 |
| Sp110 | -1.512014521 | 2.06E-14 |
| Gng12 | -1.51075059 | 9.08E-09 |
| Slc31a1 | -1.510622946 | 5.22E-22 |
| Sh3bp4 | -1.510549817 | 5.63E-14 |
| Zbtb16 | -1.50697638 | 0.000917216 |
| Dlg4 | -1.506248387 | 2.36E-11 |
| Ifi207 | -1.506131043 | 1.66E-12 |
| Spink2 | -1.505243726 | 0.0022167 |
| Gm12657 | -1.50411617 | 0.015778986 |
| Sod2 | -1.50316405 | 2.40E-10 |
| C1qc | -1.502035474 | 6.81E-11 |
| Fyb | -1.501848547 | 8.37E-09 |
| Gm20658 | -1.501587272 | 0.00919426 |
| F630028O10Rik | -1.500562688 | 0.001164008 |
| Trgc4 | -1.499914805 | 0.006135445 |
| Helz2 | -1.49948534 | 1.99E-09 |
| Tbx21 | -1.493728385 | 1.65E-09 |
| Pstpip1 | -1.493027105 | 5.64E-07 |
| Usb1 | -1.491334542 | 2.27E-18 |
| Capns2 | -1.489822895 | 0.009061272 |
| Snhg11 | -1.484963055 | 0.005460858 |
| Klrc1 | -1.481781009 | 3.60E-08 |
| Padi4 | -1.481768776 | 6.66E-06 |
| Mcf2l | -1.480828541 | 0.000162177 |
| Idnk | -1.480664203 | 1.46E-21 |
| Apobec3 | -1.479417252 | 2.21E-29 |
| Noxred1 | -1.479335403 | 0.001323957 |
| Rhov | -1.478892812 | 0.000352706 |
| Arap3 | -1.47801135 | 0.000168689 |
| Arid3a | -1.47495715 | 2.91E-05 |
| Tmem140 | -1.474512304 | 5.76E-18 |
| Lin28a | -1.472941837 | 0.007126646 |
| Zfp467 | -1.472614207 | 4.48E-11 |
| 1700020L24Rik | -1.471502576 | 0.010924655 |
| Gsdme | -1.470356706 | 0.000889153 |
| Il18rap | -1.469206935 | 4.78E-09 |
| Trim34a | -1.468211104 | 3.23E-18 |
| A530013C23Rik | -1.466781969 | 1.89E-05 |
| Gm19585 | -1.451689372 | 0.006811511 |
| Cx3cr1 | -1.451227954 | 3.21E-05 |
| Bmp1 | -1.449291562 | 0.019384069 |
| Gm10851 | -1.448174678 | 0.031740619 |
| Plk2 | -1.445613309 | 1.36E-14 |
| Sema4d | -1.445443878 | 8.31E-10 |
| H2-D1 | -1.444800027 | 6.30E-22 |
| Gm4285 | -1.444638021 | 0.000322432 |
| Alpk1 | -1.443262635 | 1.36E-08 |
| Fndc7 | -1.440795369 | 0.019362475 |
| Ccr9 | -1.439077614 | 6.46E-08 |
| Rab36 | -1.43852382 | 0.023524986 |
| Serf1 | -1.438116325 | 8.43E-09 |
| Sdhaf1 | -1.437324624 | 0.000130607 |
| 9930012K11Rik | -1.437177457 | 0.003746907 |
| Mir155hg | -1.436629664 | 4.72E-05 |
| Ip6k3 | -1.435202628 | 0.007728045 |
| Gzma | -1.433859931 | 9.69E-08 |
| Adam8 | -1.433803151 | 9.35E-07 |
| Fabp7 | -1.433194696 | 0.007143824 |
| Rnf19b | -1.43281198 | 1.41E-13 |
| Gm57366 | -1.428927413 | 0.001824938 |
| Flnb | -1.428068889 | 1.32E-11 |
| Fhl3 | -1.427824082 | 5.45E-05 |
| Trim12c | -1.426016577 | 1.90E-20 |
| Smad6 | -1.425258623 | 8.32E-08 |
| Cdh22 | -1.425064789 | 0.042250593 |
| Stx11 | -1.424705634 | 5.21E-08 |
| Snai1 | -1.424643309 | 0.006897112 |
| Dusp16 | -1.422631967 | 0.000489715 |
| Gm28809 | -1.417938755 | 0.00035753 |
| Rigi | -1.417388644 | 3.36E-13 |
| Akt3 | -1.416878732 | 2.19E-12 |
| Cflar | -1.414709785 | 2.33E-08 |
| Palld | -1.41149469 | 7.95E-09 |
| Esr1 | -1.409973436 | 0.000628626 |
| Nkg7 | -1.405575924 | 2.77E-05 |
| Hp | -1.402718532 | 0.000206266 |
| Lpcat2 | -1.402713478 | 9.50E-15 |
| Rubcnl | -1.40256443 | 6.76E-07 |
| Inpp1 | -1.397037977 | 8.40E-05 |
| Rnf144a | -1.394941123 | 1.58E-06 |
| Cd69 | -1.394273539 | 4.33E-09 |
| Sdc1 | -1.390535431 | 6.44E-10 |
| Ly6c1 | -1.387856328 | 0.00220415 |
| Stxbp5 | -1.387727165 | 6.77E-05 |
| 1500004A13Rik | -1.385531787 | 0.000353816 |
| Sfxn5 | -1.383647269 | 0.000388932 |
| Nfkbib | -1.383391886 | 1.93E-08 |
| Bace1 | -1.3829435 | 0.043892269 |
| Dpy19l3 | -1.381213714 | 0.000826164 |
| Sp140 | -1.381184508 | 5.00E-17 |
| Nfe2 | -1.380090371 | 0.000550979 |
| Dab2ip | -1.377245279 | 6.17E-05 |
| Ctsw | -1.376216195 | 5.27E-07 |
| Card19 | -1.376015778 | 3.26E-07 |
| Fcho1 | -1.375067939 | 3.13E-14 |
| Gm43814 | -1.373127916 | 9.61E-06 |
| Rgs16 | -1.37271494 | 5.94E-05 |
| Nbeal2 | -1.372007285 | 1.46E-13 |
| Ccdc180 | -1.371266754 | 0.015052615 |
| Map3k15 | -1.370909476 | 2.41E-08 |
| Ifi214 | -1.370705781 | 0.002761213 |
| Rapgef2 | -1.369778729 | 3.05E-27 |
| Ttc39c | -1.369643426 | 4.39E-16 |
| Sytl2 | -1.367481046 | 0.000376183 |
| Slfn2 | -1.367416321 | 4.27E-07 |
| Cebpe | -1.366597386 | 0.020508567 |
| Traf1 | -1.365378271 | 2.59E-13 |
| Adar | -1.364131518 | 1.85E-25 |
| Hsh2d | -1.364107517 | 6.64E-05 |
| Dach1 | -1.364051291 | 0.02773312 |
| Prdx6 | -1.363835104 | 1.08E-10 |
| Nrgn | -1.362845037 | 0.006342018 |
| Ermap | -1.36244369 | 0.012465717 |
| Ccrl2 | -1.361350621 | 1.58E-08 |
| Ehd1 | -1.358581993 | 6.00E-06 |
| Nampt | -1.354997897 | 1.16E-20 |
| Lyl1 | -1.353093922 | 1.06E-05 |
| Slamf1 | -1.352929236 | 0.00028138 |
| Pou3f1 | -1.352805954 | 0.035667337 |
| Hopx | -1.350036324 | 2.60E-13 |
| Klrg1 | -1.349785295 | 0.004603161 |
| Camk2b | -1.349009975 | 0.023531704 |
| ENSMUSG00000121780 | -1.34548186 | 0.000354996 |
| Klra4 | -1.345064855 | 0.000266071 |
| Smpd5 | -1.344789426 | 0.000153868 |
| Ptpn22 | -1.342929744 | 2.04E-17 |
| Ecm1 | -1.341230671 | 1.30E-05 |
| Met | -1.339756188 | 1.69E-11 |
| ENSMUSG00000121482 | -1.339447176 | 0.003229592 |
| Mpzl3 | -1.338348173 | 3.37E-08 |
| Siglece | -1.338054134 | 9.91E-05 |
| Chst15 | -1.337290741 | 0.000297766 |
| Acsl1 | -1.336659334 | 4.10E-19 |
| 5031434O11Rik | -1.336174447 | 0.002481931 |
| Hoxb4 | -1.335752911 | 8.69E-05 |
| Ltb4r1 | -1.334470661 | 8.13E-06 |
| Gm14636 | -1.333224521 | 0.00182203 |
| BC028528 | -1.327556211 | 1.08E-06 |
| Antxr2 | -1.327015355 | 0.000137819 |
| Pou2f2 | -1.32689603 | 0.000157843 |
| Capn5 | -1.326593652 | 2.02E-06 |
| Gm26910 | -1.325044749 | 0.038981844 |
| Gm31814 | -1.324478374 | 0.00035488 |
| Setbp1 | -1.324270221 | 0.000239245 |
| Tpst1 | -1.323748418 | 2.34E-09 |
| Mycl | -1.323664412 | 0.015352913 |
| Htra4 | -1.322793676 | 5.41E-05 |
| Msi2 | -1.319253228 | 2.78E-07 |
| Krt7 | -1.31899406 | 0.009848029 |
| Gm14321 | -1.318822432 | 0.037821583 |
| Nod1 | -1.317777617 | 2.59E-12 |
| 5830444B04Rik | -1.316106239 | 0.035675716 |
| Procr | -1.315431765 | 0.000172379 |
| Tnip1 | -1.314940304 | 0.000226709 |
| C1qa | -1.31476063 | 1.83E-09 |
| Pilrb1 | -1.312731055 | 5.74E-07 |
| Fmnl2 | -1.310930227 | 8.11E-06 |
| Gm56621 | -1.310716577 | 0.014089659 |
| Ccdc102a | -1.310122573 | 0.009681708 |
| 1600014C10Rik | -1.309547541 | 8.08E-12 |
| Pacsin1 | -1.309056097 | 0.003001393 |
| Rhou | -1.308736539 | 0.000193649 |
| Gypc | -1.30670548 | 1.51E-08 |
| Lrrc25 | -1.304861717 | 0.000140103 |
| 9930111J21Rik2 | -1.297444012 | 3.03E-17 |
| Zmynd15 | -1.296181512 | 1.17E-07 |
| Cd86 | -1.294847208 | 4.24E-07 |
| Adora2b | -1.293984279 | 0.000346024 |
| Stx2 | -1.29346639 | 4.71E-13 |
| Rhoh | -1.289765154 | 7.00E-09 |
| Fbxw17 | -1.289511456 | 2.75E-05 |
| Klrk1 | -1.288607391 | 1.08E-05 |
| Kdr | -1.286044423 | 0.008637176 |
| Sh2d3c | -1.28410388 | 1.55E-06 |
| Arhgef5 | -1.282860336 | 0.013492031 |
| Samd9l | -1.276891188 | 1.65E-10 |
| Il21r | -1.275933777 | 9.17E-06 |
| Satb1 | -1.273920984 | 1.24E-08 |
| Slc36a3 | -1.272929176 | 0.040270299 |
| Dennd3 | -1.272600137 | 9.91E-16 |
| Prkcb | -1.271949707 | 1.19E-11 |
| Slc40a1 | -1.270585088 | 2.18E-05 |
| Gm43197 | -1.27046896 | 5.20E-06 |
| Spacdr | -1.270456861 | 8.72E-07 |
| Sdc4 | -1.269089735 | 1.27E-06 |
| Hmcn2 | -1.268370293 | 0.007619669 |
| Lbp | -1.267910201 | 0.000939607 |
| Rnf114 | -1.266749224 | 1.02E-12 |
| Zfp516 | -1.266408838 | 2.87E-06 |
| Mgam | -1.265727193 | 0.004464652 |
| Ctla2a | -1.265386457 | 1.03E-06 |
| C3ar1 | -1.265280032 | 4.25E-16 |
| Serpinb8 | -1.264997845 | 6.34E-07 |
| Trappc14 | -1.264586571 | 3.83E-10 |
| Lgals3bp | -1.264435215 | 1.63E-13 |
| Ccl2 | -1.263871571 | 2.78E-07 |
| Tiam2 | -1.26332306 | 0.000132253 |
| Gm45837 | -1.262963675 | 4.20E-08 |
| Rnf145 | -1.259591464 | 3.52E-10 |
| B230303A05Rik | -1.259430045 | 0.000525085 |
| Smpdl3b | -1.259106415 | 2.09E-15 |
| Gm15265 | -1.25846686 | 0.047173876 |
| Gm28043 | -1.258346598 | 5.07E-05 |
| Acsbg1 | -1.258015375 | 0.000889153 |
| Bach2os | -1.257651969 | 0.00023255 |
| Gm28192 | -1.256734255 | 0.041230258 |
| Tbc1d8 | -1.255817332 | 8.49E-08 |
| Itga2 | -1.254961498 | 0.001866511 |
| Gpc2 | -1.254670113 | 0.007284125 |
| Ptprf | -1.252937429 | 0.002759564 |
| Ltb | -1.251820452 | 1.65E-07 |
| Tg | -1.251727756 | 0.040200127 |
| Fbxl9 | -1.250691796 | 0.00266054 |
| Ptafr | -1.250552019 | 3.46E-07 |
| Abtb1 | -1.249748957 | 5.97E-06 |
| Acp3 | -1.247804891 | 0.000275523 |
| Cyp4f18 | -1.244407895 | 0.00015961 |
| S100a6 | -1.242694683 | 4.87E-05 |
| Dok1 | -1.242421361 | 1.33E-12 |
| Dnaaf3 | -1.239219339 | 0.010701536 |
| Rflnb | -1.23715797 | 0.003038237 |
| Smox | -1.236626182 | 2.29E-06 |
| A530064D06Rik | -1.234109497 | 8.74E-07 |
| E130102H24Rik | -1.233663133 | 0.014092933 |
| Uqcc5 | -1.232830116 | 0.003755089 |
| Bcl9 | -1.232673524 | 4.35E-07 |
| Klrd1 | -1.232143704 | 1.24E-05 |
| Pigr | -1.23147458 | 0.006429533 |
| Pilrb2 | -1.230593512 | 6.36E-06 |
| Adap2 | -1.229815063 | 2.17E-12 |
| Pvrig | -1.229720536 | 0.021964846 |
| Klra8 | -1.22954532 | 0.000237248 |
| Tent5c | -1.228122647 | 1.31E-08 |
| Plekhn1 | -1.227665306 | 1.56E-11 |
| Siglech | -1.226780494 | 0.00013513 |
| Whamm | -1.224650939 | 4.90E-10 |
| B230217C12Rik | -1.220331247 | 0.008363967 |
| Neurl3 | -1.218755362 | 1.06E-07 |
| Syt11 | -1.21810433 | 1.65E-12 |
| Nrarp | -1.216013587 | 0.000893886 |
| Cabyr | -1.213764497 | 0.003572891 |
| Slc25a37 | -1.210997495 | 6.86E-08 |
| Itgam | -1.205480401 | 3.90E-05 |
| Ccr5 | -1.204096422 | 8.94E-12 |
| Rac2 | -1.202903353 | 2.65E-05 |
| Mtus1 | -1.201939539 | 9.77E-08 |
| Loxl3 | -1.19923695 | 0.016209007 |
| Gm38378 | -1.197766124 | 0.043025682 |
| Gm37513 | -1.197766124 | 0.043025682 |
| Wdfy1 | -1.197124214 | 1.73E-05 |
| Mxd1 | -1.196892798 | 0.000390397 |
| F2rl2 | -1.192535565 | 0.010890701 |
| Eng | -1.190806419 | 0.000829032 |
| Gm26797 | -1.190781482 | 0.017903804 |
| Unc93b1 | -1.190727825 | 8.80E-15 |
| Slc43a2 | -1.189105027 | 1.48E-06 |
| Irak3 | -1.188404125 | 0.002233097 |
| Rasgrp4 | -1.188189875 | 0.000986307 |
| Lpar6 | -1.18789812 | 2.86E-06 |
| Maf | -1.186158912 | 6.14E-11 |
| Ankrd37 | -1.186011695 | 0.002419795 |
| Gm5831 | -1.185885101 | 0.041776009 |
| Haghl | -1.184800195 | 0.00012168 |
| C4b | -1.18116678 | 0.001260519 |
| Gm49368 | -1.178753712 | 0.006416194 |
| Clec4e | -1.178107929 | 3.24E-06 |
| Flt3l | -1.177721344 | 1.96E-08 |
| Sorl1 | -1.175842973 | 1.26E-05 |
| Gm16181 | -1.174067189 | 0.038925538 |
| Gdpd5 | -1.172082294 | 2.20E-05 |
| Cdc42ep2 | -1.171713667 | 6.28E-05 |
| Tbc1d2b | -1.171615762 | 2.49E-07 |
| Irf5 | -1.171216963 | 5.41E-15 |
| Tinagl1 | -1.171200507 | 0.030203096 |
| 2310015A10Rik | -1.170584642 | 0.000742987 |
| Ogfrl1 | -1.17055637 | 0.000312326 |
| Gnb4 | -1.170249474 | 0.001889266 |
| Fanca | -1.169500325 | 0.000206998 |
| H2-K2 | -1.1684007 | 2.26E-06 |
| Padi2 | -1.166732171 | 3.32E-05 |
| Lyst | -1.166208031 | 0.000420749 |
| Sestd1 | -1.165984491 | 2.13E-12 |
| Zfp691 | -1.165883133 | 1.60E-06 |
| Mylip | -1.165597836 | 1.75E-10 |
| Tarm1 | -1.165324178 | 0.00864636 |
| Dusp6 | -1.16323082 | 6.72E-08 |
| Pdlim2 | -1.161687756 | 0.000136032 |
| Id2 | -1.161163737 | 3.80E-05 |
| Abi3 | -1.160786425 | 5.43E-11 |
| Kctd17 | -1.160539432 | 4.19E-07 |
| Pdxp | -1.159734935 | 0.0009214 |
| Apobec1 | -1.159651992 | 3.73E-15 |
| Nfkbie | -1.158933544 | 4.32E-09 |
| ENSMUSG00000121481 | -1.158213372 | 0.049709241 |
| Gm39556 | -1.158058393 | 8.35E-05 |
| Pkp3 | -1.155036169 | 0.000137211 |
| Krt8 | -1.154994974 | 0.007759804 |
| Gzmb | -1.154096083 | 0.00014497 |
| Prkce | -1.153828764 | 9.51E-08 |
| Tmem243 | -1.150401554 | 1.86E-09 |
| Xdh | -1.149263749 | 4.21E-18 |
| Ralgps1 | -1.147698427 | 1.42E-05 |
| Igsf6 | -1.146631142 | 6.10E-05 |
| Dusp4 | -1.145741481 | 1.31E-06 |
| Gas7 | -1.143877497 | 7.13E-05 |
| D730003I15Rik | -1.143421317 | 0.043224767 |
| H2-Oa | -1.14338535 | 2.88E-05 |
| Ttc39b | -1.143099016 | 2.98E-17 |
| Tnfaip3 | -1.141187355 | 3.66E-06 |
| Rab20 | -1.137830826 | 2.69E-05 |
| Zfp119b | -1.136651838 | 0.008783367 |
| F730311O21Rik | -1.13611719 | 0.009737099 |
| Adam11 | -1.135339809 | 0.001007051 |
| Dusp2 | -1.134952115 | 0.001123529 |
| Cry1 | -1.1347765 | 0.000279215 |
| Ethe1 | -1.134756539 | 2.03E-07 |
| Ncf4 | -1.133639068 | 7.74E-05 |
| Id3 | -1.130824296 | 8.07E-05 |
| Mndal | -1.130656947 | 0.000157991 |
| Cfap410 | -1.129223515 | 5.68E-08 |
| Lrig1 | -1.126727451 | 0.046872828 |
| Zfp281 | -1.126000372 | 2.37E-08 |
| Slc16a13 | -1.124086415 | 0.009557875 |
| F10 | -1.122456386 | 0.00011337 |
| Pld4 | -1.121478637 | 3.93E-08 |
| 1700109H08Rik | -1.120084879 | 0.013067018 |
| Ggt5 | -1.119979167 | 0.009909059 |
| Rbm38 | -1.119918788 | 0.000878689 |
| Gvin-ps6 | -1.118593545 | 0.01112029 |
| Runx3 | -1.118572724 | 1.94E-07 |
| Hk1 | -1.117126808 | 1.35E-17 |
| Tbkbp1 | -1.117118856 | 4.66E-11 |
| Lynx1 | -1.116816423 | 0.004593825 |
| Zup1 | -1.115298741 | 7.91E-06 |
| Nlrp12 | -1.114714207 | 0.004174224 |
| Myl9 | -1.114531498 | 0.017015186 |
| Gm20559 | -1.113142911 | 3.16E-08 |
| Tnfrsf18 | -1.11122232 | 1.40E-05 |
| F2r | -1.109547014 | 0.001826437 |
| Il1a | -1.10895056 | 0.002833976 |
| Il18r1 | -1.107752294 | 2.01E-06 |
| Lifr | -1.106958485 | 9.20E-07 |
| Pira12 | -1.10612517 | 0.006998794 |
| H2ac19 | -1.103079206 | 0.001220427 |
| Nuak1 | -1.100201229 | 3.35E-06 |
| Gm7160 | -1.099944509 | 0.00240132 |
| Col4a2 | -1.099246205 | 8.50E-05 |
| Irf8 | -1.098405353 | 1.11E-05 |
| Cblb | -1.097833749 | 4.10E-11 |
| Zap70 | -1.096110139 | 0.001027448 |
| Plbd1 | -1.095741354 | 3.79E-05 |
| Jag2 | -1.094796489 | 0.009512627 |
| Fgd3 | -1.093245025 | 0.000651933 |
| Glrx | -1.091664801 | 2.71E-11 |
| Ccl4 | -1.090952736 | 0.005868053 |
| Sh3bp1 | -1.090139061 | 1.36E-11 |
| 4833419F23Rik | -1.089062344 | 0.035177068 |
| Zeb1 | -1.088786783 | 0.000314227 |
| Ifitm2 | -1.088468737 | 0.002383482 |
| Phospho1 | -1.088264523 | 0.001789313 |
| Kctd1 | -1.084796532 | 0.020285286 |
| Clcn7 | -1.083128384 | 1.34E-17 |
| Bid | -1.082878293 | 7.28E-07 |
| Tsc22d3 | -1.082673382 | 0.010825375 |
| 1700025G04Rik | -1.082627882 | 0.000627278 |
| Rnaset2a | -1.081445041 | 6.86E-09 |
| Rasip1 | -1.081250128 | 0.000910332 |
| Themis2 | -1.08037069 | 6.94E-09 |
| Pim2 | -1.080148298 | 0.000223349 |
| Timd4 | -1.08007245 | 0.044283571 |
| A630001O12Rik | -1.079350968 | 0.004996478 |
| B230303A05Rik | -1.078432502 | 0.001943388 |
| Sh2d2a | -1.078188939 | 0.000112406 |
| Pdcd1lg2 | -1.077854807 | 0.002048321 |
| Ssbp2 | -1.076588785 | 0.006880921 |
| Gm43305 | -1.075824021 | 0.000313555 |
| Cfb | -1.075130231 | 2.51E-08 |
| Gm34643 | -1.073778819 | 0.008018324 |
| Klre1 | -1.072109468 | 0.022615693 |
| Hcst | -1.071096515 | 0.000294279 |
| Hip1r | -1.070791694 | 0.001989869 |
| Klra3 | -1.068680689 | 0.03652863 |
| Pml | -1.066773725 | 1.13E-07 |
| Vasp | -1.066697111 | 4.92E-05 |
| Sema4a | -1.065490565 | 6.50E-11 |
| Lox | -1.06498177 | 0.002301312 |
| Klrb1b | -1.06402382 | 1.00E-05 |
| Pnp2 | -1.063795933 | 0.003072159 |
| Alkbh4 | -1.063750581 | 1.80E-05 |
| Hpn | -1.063037239 | 0.04765071 |
| Gm45472 | -1.062225682 | 0.000983781 |
| Myo10 | -1.061656736 | 1.17E-05 |
| Castor2 | -1.061112596 | 2.31E-06 |
| Thy1 | -1.058852906 | 0.000689593 |
| Dtx2 | -1.057514319 | 2.98E-05 |
| Armc7 | -1.056173471 | 0.001114184 |
| Nqo1 | -1.055930126 | 0.041526182 |
| Eomes | -1.053617112 | 0.001187081 |
| Cxcr6 | -1.052536767 | 0.028780702 |
| Traf3ip2 | -1.051856658 | 0.000547691 |
| Mllt6 | -1.049250124 | 7.81E-06 |
| Mfsd6l | -1.049225798 | 0.016118977 |
| Ggct | -1.047415371 | 4.52E-06 |
| E230029C05Rik | -1.04737789 | 0.007902673 |
| Plau | -1.047006869 | 0.000181946 |
| Epha2 | -1.046586559 | 0.011203611 |
| Card6 | -1.045035127 | 2.96E-05 |
| Ifi203 | -1.043954182 | 0.005213137 |
| Gm44502 | -1.043536002 | 0.034402253 |
| C5ar1 | -1.041197194 | 0.002464055 |
| Abcb1a | -1.041150377 | 0.011888885 |
| Kcnn4 | -1.038619694 | 5.32E-05 |
| Slc7a8 | -1.037575477 | 2.41E-11 |
| Foxred2 | -1.036625243 | 0.003533428 |
| Eif2ak2 | -1.036369998 | 1.29E-06 |
| Tcea2 | -1.034990111 | 0.002436916 |
| Mafb | -1.033022342 | 6.10E-08 |
| Klrb1f | -1.032424385 | 0.009897096 |
| Map3k9 | -1.030769314 | 0.005269133 |
| Zfp719 | -1.028468153 | 0.004169693 |
| 1500011B03Rik | -1.027698299 | 0.000513404 |
| Gm9923 | -1.027335203 | 0.0336415 |
| Irf2 | -1.027175042 | 6.64E-10 |
| Il2rg | -1.026997749 | 3.84E-10 |
| Birc3 | -1.025311204 | 1.12E-05 |
| Fpr1 | -1.024839572 | 0.017889632 |
| Osbpl3 | -1.023958418 | 3.18E-18 |
| Gm20100 | -1.023866145 | 0.031482852 |
| Reck | -1.022871019 | 0.012878606 |
| Nlrp1a | -1.022702881 | 5.21E-06 |
| Rin3 | -1.022394213 | 0.006353349 |
| Tmem38a | -1.02219792 | 0.039034622 |
| Gm5617 | -1.021971513 | 0.004179317 |
| Hcls1 | -1.021880251 | 3.45E-09 |
| Erap1 | -1.021224278 | 1.58E-08 |
| 4930581F22Rik | -1.020145633 | 0.025521305 |
| A330023F24Rik | -1.019781239 | 0.009864392 |
| Tnfaip8l3 | -1.019395249 | 1.58E-05 |
| Casp4 | -1.018938148 | 6.51E-06 |
| Rrad | -1.017933633 | 3.03E-05 |
| Taf7 | -1.017850645 | 0.000439843 |
| Celsr1 | -1.017729911 | 0.023902039 |
| Ifnar2 | -1.016619983 | 2.33E-11 |
| Gm56909 | -1.016351448 | 0.021138854 |
| Arhgap27 | -1.015334888 | 1.65E-09 |
| Hspa1b | -1.013121007 | 6.97E-06 |
| Ada | -1.012739568 | 0.006402101 |
| Xkr8 | -1.012260912 | 0.004092047 |
| Rnf135 | -1.011692467 | 5.58E-05 |
| Papss2 | -1.011416972 | 1.86E-07 |
| Nfkbia | -1.011341995 | 0.000492604 |
| Batf | -1.01031623 | 0.004691046 |
| Zfp36l1 | -1.009317972 | 0.001718652 |
| 2310043P16Rik | -1.008054942 | 0.009124152 |
| Tor3a | -1.008026937 | 1.20E-05 |
| Gm33887 | -1.007996466 | 0.005740025 |
| Nxn | -1.006344482 | 6.24E-05 |
| Mdk | -1.005946648 | 0.002208474 |
| Klf2 | -1.005394732 | 0.000314392 |
| Igfbp4 | -1.004957911 | 0.008161061 |
| Cxcl16 | -1.004678175 | 3.15E-06 |
| Rnf34 | -1.003734206 | 6.03E-10 |
| Cacnb1 | -1.003190525 | 0.006801324 |
| Lat2 | -1.002862807 | 4.74E-10 |
| Ppp1r16b | -1.001522216 | 0.000114565 |
| Slc15a3 | -1.001280675 | 2.83E-06 |
| Ndst1 | -1.0010451 | 0.000755462 |
