# Supplementary material for: Lung‐delivered IL‐10 mitigates Lung inflammation induced by repeated endotoxin exposures in male mice
Source: Physiol Rep. 2025 Feb 20;13(4):e70253. doi: 10.14814/phy2.70253 (PMC11842461; doi:10.14814/phy2.70253)
Supplement: Supplementary file 4 — Table S1. [file PHY2-13-e70253-s002.docx]

**Tables**

| **Supplemental Table S1.** Table of flow cytometry antibodies. | | | |  |
| --- | --- | --- | --- | --- |
| **Antigen** | **Species** | **Clone** | **Vendor** | **Fluorophore** |
| CD45 | Rat anti-mouse | 30-F11 | BD Biosciences (563053) | BV605 |
| CD11b | Rat anti-mouse | M1/70 | BD Biosciences (550993) | PerCP-Cy5.5 |
| Ly6G | Rat anti-mouse | 1A8 | BD Biosciences (561236) | AF700 |
| CD11c | Rat anti-mouse | N418 | Invitrogen  (61-0114-82) | PE-eFluor610 |
| CD4 | Rat anti-mouse | RM4-5 | BD Biosciences (553047) | FITC |
| CD8a | Rat anti-mouse | 53-6.7 | BD Biosciences (553033) | PE |
| CD19 | Rat anti-mouse | 1D3 | Invitrogen  (47-0193-82) | APC-eFluor780 |
| CD3e | Hamster anti-mouse | 145-2C11 | BD Biosciences (552774) | PE-Cy7 |
| NK1.1 | Mouse anti-mouse | PK136 | BD Biosciences (550627) | APC |

| **Supplemental Table S2.** Blood chemistry panel of mice exposed to LPS followed by IL-10 treatment daily for 3 days. | | | |  |
| --- | --- | --- | --- | --- |
|  | **Saline** | | **LPS** | |
|  | **Vehicle** | **IL-10** | **Vehicle** | **IL-10** |
| **Serum** |  |  |  |  |
| ALT (U/L) | 34.4 ± 13.4 | 29.6 ± 6.11 | **16.2 ± 2.11^###^** | 21.9 ± 3.10 |
| BUN (mmol/L) | 8.10 ± 1.03 | 8.50 ± 0.735 | 6.90 ± 1.31 | 7.82 ± 0.721 |
| Creatinine (μmol/L) | 22.2 ± 6.94 | 18.8 ± 1.79 | 18.2 ± 0.667 | 18.8 ± 1.72 |
| Sodium (mmol/L) | 153.0 ± 3.78 | 153.0 ± 6.38 | 154.0 ± 3.04 | 153.0 ± 4.33 |
| Potassium (mmol/L) | 6.12 ± 1.32 | 6.66 ± 1.44 | 6.37 ± 0.274 | 6.17 ± 0.215 |
| Chloride (mmol/L) | 108.0 ± 2.39 | 106.0 ± 1.87 | 105.0 ± 1.39 | 108.0 ± 2.55 |
| CO_2_ (mmol/L) | 20.6 ± 1.95 | 18.8 ± 1.79 | 22.2 ± 2.39 | 20.6 ± 3.17 |
| Mean ± SD. Statistical difference vs. Saline + Vehicle (^#^p<0.05, ^###^p<0.001) and statistical significance between Vehicle and IL-10 treatments (^*^p<0.05) and **bold.**  n=5-9 mice/group | | | | |

| **Supplemental Table S3.** Peripheral blood monocytes and lymphocytes of mice exposed to LPS and IL-10 treatment daily for 3 days. | | | |
| --- | --- | --- | --- |
|  | **Saline** | **LPS** | |
|  | **Vehicle** | **Vehicle** | **IL-10** |
| **Peripheral Blood**, % |  |  |  |
| Monocytes | 5.00 ± 2.12 | 7.20 ± 5.63 | 3.40 ± 1.52 |
| Lymphocytes | 70.6 ± 6.39 | **51.6 ± 16.7^#^** | **83.6 ± 5.94^#,*^** |
| Mean ± SD. Statistical difference vs. Saline + Vehicle (^#^p<0.05, ^##^p<0.01) and statistical significance between Vehicle and IL-10 treatments (^**^p<0.01, ^***^p<0.001) and **bold**.  n=5 mice/group | | | |

| **Supplemental Table S4.** Repetitive LPS-exposure induced lung and BALF responses in repeated IL-10 and vehicle treated mice. | | | | |
| --- | --- | --- | --- | --- |
|  | **Saline** | | **LPS** | |
|  | **Vehicle** | **IL-10** | **Vehicle** | **IL-10** |
| **BALF cells**, x10^5^ |  |  |  |  |
| Neutrophils | 0.58 ± 0.093 | 0.037 ± 0.041 | **32.0 ± 19.3^###^** | **18.8 ± 7.69^#^** |
| Macrophages | 0.71 ± 0.5 | 0.75 ± 0.26 | **2.92 ± 1.69^#^** | **2.31 ± 0.88^#^** |
| Lymphocytes | 0.0016 ± 0.0023 | 0.0016 ± 0.0026 | **0.43 ± 0.35^#^** | **0.42 ± 0.33^#^** |
| **BALF mediators** |  |  |  |  |
| CCL7 (pg/ml) | 0.00 ± 0.00 | 0.00 ± 0.00 | 778.8 ± 396.9 | 718.6 ± 406.8 |
| C5a (μg/ml) | 6.35 ± 1.79 | 5.57 ± 1.33 | 7.01 ± 1.86 | 6.19 ± 1.69 |
| Fibronectin (ng/ml) | 31.47 ± 41.56 | 79.4 ± 11.42 | **933.7 ± 426.0^####^** | **610.1 ± 206.0^##^** |
| TGF-β (pg/ml) | 0.044 ± 0.032 | 0.055 ± 0.051 | 2.88 ± 7.43 | 16.89 ± 34.47 |
| **Lung cells**, x10^5^ |  |  |  |  |
| CD3^+^CD4^+^ T cells | 0.045 ± 0.05 | 0.61 ± 0.23 | **1.00 ± 0.44^##^** | **0.88 ± 0.21^#^** |
| CD19^+^ B cells | 2.04 ± 0.35 | 2.43 ± 0.96 | **4.54 ± 2.57^#^** | 3.81 ± 0.92 |
| **Lung mediators** |  |  |  |  |
| CCL7 (pg/ml) | 83.58 ± 47.58 | 204.0 ± 57.69 | **1728 ± 815.2^####^** | **934.6 ± 635.3^##^** |
| MMP-3 (ng/ml) | 8.15 ± 3.11 | 9.87 ± 2.4 | **46.2 ± 23.98^#^** | **59.97 ± 30.38^##^** |
| TGF-β (pg/ml) | 0.28 ± 0.16 | 0.48 ± 0.16 | **265.8 ± 215^##^** | **237.4 ± 181.6^##^** |
| Mean ± SD. Statistical difference vs. Saline + Vehicle (^#^p<0.05, ^##^p<0.01, ^###^p<0.001, ^####^p<0.001) and **bold.** n=5-10 mice/group | | | |  |

**Supplemental Table S5.** Top differentially expressed genes by fold change with repetitive IL-10 treatment post-repeated LPS exposure.

| **Gene** | **Name and Biological Process** |
| --- | --- |
| ***Upregulated Genes*** | |
| *Pwwp4b* | PWWP domain containing 4B; protein coding gene of unknown function |
| *Rpl10-ps5* | Ribosomal protein L10, pseudogene 5; nonfunctional DNA segment |
| *Fcrl2* | Fc receptor-like 2; regulatory role in normal and neoplastic B cell development |
| *Il5* | Interleukin 5; Maturation, activation, and migration of eosinophils and basophils |
| *Dgkeos* | Diacylglycerol kinase, epsilon, opposite strand; non-coding RNA |
| *Lpar4* | Lysophosphatidic acid receptor 4; role in monocyte differentiation |
| *Sh2d1b2* | SH2 domain containing 1B2; regulation of natural killer cell mediated cytotoxicity |
| *Sowahd* | Sosondowah ankyrin repeat domain family member D; coding gene of unknown function |
| *Odf3l1* | Outer dense fiber protein 3-like protein 1; cytoskeleton assembly and cilia organization |
| *Klk1b11* | Kallikrein 1-related peptidase b11; hydrolase activity, acting on carbon-nitrogen bonds |
| ***Downregulated Genes*** | |
| *Ubd* | Ubiquitin D; positive regulation of I-κB kinase/NF-κB signaling and apoptotic processes |
| *Tgtp1* | T cell specific GTPase 1; involved in cellular response to interferons (i.e., α, β, γ) |
| *Tmem45a2* | Transmembrane protein 45A2; orthologous to human *Tmem45a* highly expressed in skin |
| *Sectm1a* | Secreted and transmembrane 1A; epithelial product that sustains neutrophil inflammation |
| *Iigp1* | Interferon inducible GTPase 1; cellular response to interferon-β and Gram-neg bacteria |
| *Serpina3f* | Serine protease inhibitor A3F; response to bacteria, cytokines, and peptide hormones |
| *Cxcl11* | Chemokine (C-X-C motif) ligand 11; cellular response to LPS and neutrophil chemotaxis |
| *Ly6i* | Lymphocyte antigen 6 family member I; involved in acetylcholine receptor signaling |
| *Gzmf* | Granzyme F; target cell lysis in cell-mediated immune responses |
| *Orm2* | Orosomucoid 2; transport protein in blood, modulates acute-phase response |

Genes were ranked according to the fold-change of significantly (p<0.05) up- or downregulated genes (IL-10 vs. PBS). Only named genes were included.

n=3 mice/group

| **Supplemental Table S6.** Repeated LPS-exposure induced lung and BALF responses with IL-10 vs. vehicle treated mice 1 week after final LPS exposure. | | |
| --- | --- | --- |
|  | **LPS** | |
|  | **Vehicle** | **IL-10** |
| **Serum,** pg/ml |  |  |
| IL-6 | 1.44 ± 0.88 | 2.66 ± 2.59 |
| CXCL1 | 42.78 ± 5.01 | 52.99 ± 18.11 |
| **BALF cells**, x10^5^ |  |  |
| Neutrophils | 0.091 ± 0.099 | 0.023 ± 0.033 |
| Macrophages | 2.44 ± 0.74 | **1.13 ± 0.27^***^** |
| Lymphocytes | 0.22 ± 0.16 | **0.055 ± 0.031^**^** |
| **Lung tissue cells**, x10^5^ |  |  |
| Alveolar Mφ | 3.93 ± 1.55 | 3.38 ± 1.07 |
| B Cells | 1.59 ± 0.82 | 1.26 ± 0.59 |
| NK Cells | 0.59 ± 0.19 | 0.58 ± 0.21 |
| CD4^+^ T Cells | 0.60 ± 0.19 | 0.47 ± 0.064 |
| CD8^+^ T Cells | 0.24 ± 0.17 | 0.17 ± 0.081 |
| **BALF mediators** |  |  |
| C5a (μg/ml) | 14.70 ± 3.51 | **8.77 ± 1.39^****^** |
| CCL2 (pg/ml) | 14.54 ± 10.27 | 11.37 ± 2.94 |
| CCL7 (pg/ml) | 0.00 ± 0.00 | 0.00 ± 0.00 |
| CXCL1 (pg/ml) | 11.71 ± 1.62 | 9.62 ± 4.21 |
| Fibronectin (ng/ml) | 783.3 ± 280.5 | **283.0 ± 72.63^****^** |
| IL-6 (pg/ml) | 0.078 ± 0.17 | 0.00 ± 0.00 |
| IL-10 (pg/ml) | 2.53 ± 5.72 | **20.26 ± 19.27^**^** |
| TGF-β (pg/ml) | 70.1 ± 52.88 | 72.07 ± 46.27 |
| TNF-α (pg/ml) | 0.00 ± 0.00 | 0.00 ± 0.00 |
| **Lung mediators** |  |  |
| CCL7 (pg/ml) | 164.2 ± 82.07 | 122.8 ± 94.94 |
| IL-6 (pg/ml) | 19.02 ± 2.42 | **23.02 ± 2.35^*^** |
| MMP-3 (μg/ml) | 40.91 ± 12.25 | 59.43 ± 36.09 |
| MMP-8 (ng/ml) | 27714 ± 5015 | 30500 ± 17301 |
| TIMP-1 (μg/ml) | 6.45 ± 0.984 | 6.29 ± 2.62 |
| TGF-β (pg/ml) | 990.7 ± 704.0 | 491.7 ± 261.9 |
| TNF-α (pg/ml) | 0.97 ± 0.96 | 0.00 ± 0.00 |
| Mean ± SD. Statistical difference vs. LPS + Vehicle (^*^p<0.05, ^**^p<0.01, ^***^p<0.001, ^****^p<0.001) and **bold**.  n=5-10 mice/group | | |
